# Serum and urinary metabolomics and outcomes in cirrhosis

**DOI:** 10.1371/journal.pone.0223061

**Published:** 2019-09-27

**Authors:** Jasmohan S. Bajaj, Sili Fan, Leroy R. Thacker, Andrew Fagan, Edith Gavis, Melanie B. White, Douglas M. Heuman, Michael Fuchs, Oliver Fiehn

**Affiliations:** 1 Division of Gastroenterology, Hepatology and Nutrition, Virginia Commonwealth University and McGuire VA Medical Center, Richmond, Virginia, United States of America; 2 West Coast Metabolomics Center, University of California, Davis, California, United States of America; 3 Department of Biostatistics, Virginia Commonwealth University, Richmond, Virginia, United States of America; Medizinische Fakultat der RWTH Aachen, GERMANY

## Abstract

**Background:**

Cirrhosis can alter several metabolic pathways. Metabolomics could prognosticate outcomes like hepatic encephalopathy (HE), transplant, hospitalization and death.

**Aim:**

Determine changes in serum and urine metabolomics in cirrhotics who develop outcomes.

**Methods:**

Cirrhotic outpatients underwent data, serum/urine collection and were followed for 90 days. Demographics, cirrhosis details and medications were collected. Metabolomics was performed on urine/serum using GC/MS with subsequent bioinformatics analyses (ChemRICH, MetaMAPP and PLS-DA). Logistic regression adjusting for covariates (demographics, alcohol etiology, prior HE, PPI, SBP prophylaxis, rifaximin/lactulose) were performed and ROC curves comparing MELD to adjusted serum & urine metabolites were created.

**Results:**

211 patients gave serum, of which 64 were hospitalized, 19 developed HE, 13 were transplanted and 11 died. 164 patients gave urine of which 56 were hospitalized, 18 developed HE, 12 were transplanted and 11 died. Metabolomics: Saturated fatty acids, amino acids and bioenergetics-related metabolites differentiated patients with/without outcomes. After regression, 232, 228, 284 and 229 serum metabolites were significant for hospitalization, HE, death and transplant. In urine 290, 284, 227 & 285 metabolites were significant for hospitalization, HE, death and transplant respectively. AUC was higher for serum metabolites vs MELD for HE (0.85 vs.0.76), death (0.99 vs.0.88), transplant (0.975 vs.0.94) and hospitalizations (0.84 vs.0.83). Similarly, urinary metabolite AUC was also higher than MELD for HE (0.87 vs.0.72), death (0.92 vs 0.86), transplant (0.99 vs.0.90) and hospitalizations (0.89 vs.0.84).

**Conclusions:**

In this exploratory study, serum and metabolites focused on lipid, bioenergetics and amino acid metabolism are altered in cirrhotics who develop negative outcomes.

## Introduction

The development of complications in cirrhosis can result in hospitalizations, death and need for liver transplant[1]. These complications such as hepatic encephalopathy (HE) are a major burden[2]. Currently, the prediction of outcomes is focused on clinical severity scores and the presence of complications[3]. Other biomarkers related to inflammation and microbiota have been reported but have not been studied across a breadth of outcomes[4–7]. In prior studies, metabolomics is helpful in predicting survival in decompensated cirrhosis, could differentiate between patients with without HE, and could be used to follow therapy withdrawal[8–11]. They are also able to provide pathophysiological insight into the development of these complications, including HE. However, the impact of blood and urine metabolites in those who experience a broader range of outcomes and their potential role in the prediction of these outcomes needs to be studied.

In this exploratory study, our aim was to determine the alterations in serum and urine metabolomics at baseline in patients who developed outcomes, and to study the additional impact of serum and urine metabolomics in the prediction of clinically relevant outcomes such as HE, hospitalizations, transplant and death over 90 days in outpatients with cirrhosis adjusted for clinical biomarkers.

## Materials and methods

We recruited cirrhotic outpatients prospectively from GI and Hepatology Clinics at Virginia Commonwealth University and McGuire VA Medical Centers after informed consent. Cirrhosis was diagnosed using either liver biopsy, cirrhotic liver on imaging, frank decompensation (ascites, HE, prior variceal bleeding) or evidence of varices in patients with chronic liver disease. We excluded patients with an unclear diagnosis of cirrhosis, prior organ transplant, HIV infection, hepatocellular cancer (HCC), those unable to consent, unable to provide any sample or those who were not willing to allow follow-up reviews. We obtained written informed consent from all participants after IRB approval at Virginia Commonwealth University and McGuire VA Medical centers (approval numbers BAJAJ004, BAJAJ015, HM13191 and HM13466). The population was recruited between October 2015 and November 2016 and is representative of patients with cirrhosis found in this region of the world.

Patients were then followed for 90 days for evaluation of (a) hospitalizations (b) overt HE (c) transplant and (d) death. Only non-elective hospitalizations were considered. Overt HE was defined as grade≥ 2 on West-Haven criteria[2]. These follow-ups were performed as part of a scheduled chart review at days 30 and 90 post-enrollment. If no follow-up was noted in the chart review and the patient was still alive and without transplant, they were called to inquire about hospitalizations and other outcomes that may have required interventions at other facilities.

Data collected were demographics, diabetes, etiology of cirrhosis (alcohol/not), MELD score, prior HE, use of PPI, lactulose, rifaximin and SBP prophylaxis. Fasting morning serum and urine samples were collected. These were adjusted for in the final analysis of the serum and urine metabolites with respect to individual outcomes apart from MELD score, which was used as the clinical comparator. The biological MELD score was calculated without exception points apart from if the patient was on dialysis.

### Metabolomics analysis methods

Serum and urine were analyzed for multi-variate metabolomics at the NIH West Coast Metabolomics Center using published GC-TOF MS techniques[12] (Supplementary methods).

### Statistical analysis methods

The data was first transformed using the generalized log 10 transformation and then auto-scaled[13]. Using the statistical analysis website Metabox, we performed multivariate logistic regressions based on each single metabolite against each outcome, having age, gender, diabetes, alcoholic etiology, prior HE, PPI, SBP prophylaxis, rifaximin use as covariates[14]. We concluded statistical significance with p<0.05 and regression coefficients were used as a measure of the effect size. Benjamini-Hochberg procedure was used to control the false discovery rate (FDR).

Two analyses were performed to determine the changes in metabolomics and their effect on outcomes.

*Analysis 1*: was to determine the inter-relationship and pathophysiology of the metabolites that were significantly associated with individual outcomes. We performed chemical similarity enrichment analysis (ChemRICH) to provide chemical classes significantly altered in patients who developed a particular outcome compared to the rest[15]. ChemRICH performed enrichment analysis based on the chemical structures that are not defined by the pathways, which can be inherently flawed and depended on the background databases. The p-values of the clusters were obtained by employing the Kolmogorov-Smirnov test. A p-value less than 0.05 indicates a statistically significant enriched compound cluster. In addition, the compound significance, the effect sizes and the altering directions were visualized by MetaMapp[16]. Individual metabolite VIP scores were calculated for each outcome and the top 20 metabolites by the VIP score for each outcome were further analyzed.
*Analysis 2*: was the comparison of the predictability of using urine and serum metabolites and using MELD respectively. We first adjusted the confounder effects on the urine and serum data. Then the adjusted urine and serum metabolites and the MELD score was used to build the partial least square–discriminant analysis (PLS-DA) for each of the outcomes. The predictions were made based on leave-one-out cross-validation procedure, and the area under the ROC curve (AUC) was used to access the predicting power of each PLS-DA models. These were compared statistically.

## Results

We recruited 211 cirrhotic outpatients (Table 1) who gave serum, of which 164 patients provided urine samples. Of the 211 patients, at 90 days, 64 were hospitalized, 19 developed an HE episode, 13 were transplanted and 11 died. In those who gave urine (Table 1), 56 were hospitalized, 18 developed an HE episode, 12 were transplanted and 11 died. Of the people who did not provide urine, the majority were on dialysis (n = 35), while the rest were not willing to provide it. Patients with an outcome (death, overt HE, transplant or hospitalization), largely had a higher MELD score, lactulose, SBP prophylaxis and prior HE compared to the rest. Age, gender, diabetes, rifaximin use and PPI were largely non-significant. Prior HE did not affect transplant but there was a difference in gender between those did or did not get transplanted. Hospitalizations were due to liver-related causes in the majority (n = 43, HE, n = 19, renal/metabolic causes, n = 13, ascites/anasarca n = 9, others n = 2) followed by infection (n = 13, SBP n = 4, Urinary tract infections n = 4, pneumonia n = 3 and *C*.*difficile* n = 2) and liver-unrelated in the rest (n = 6, cardiovascular n = 3, pulmonary n = 2, other n = 1). Of the 13 renal/metabolic causes, 7 patients had acute kidney injury (AKI) without hepato-renal syndrome, 3 had hyponatremia, 1 had hypernatremia and 2 had hepato-renal syndrome. None of the patients developed HCC or variceal bleeding during the follow-up.

**Table 1:** Clinical characteristics of patients who gave serum and urine and developed outcomes within 90 days.

**Patients who gave serum**	**All patients** **(n = 211)**	**Hospitalization**		**Overt HE**		**Death**		**Transplant**	
		**No** **(n = 147)**	**Yes** **(n = 62)**	**No** **(n = 192)**	**Yes** **(n = 19)**	**No** **(n = 200)**	**Yes** **(n = 11)**	**No** **(n = 198)**	**Yes** **(n = 13)**
Age (median, IQR)	58.0 (7.5)	58.0 (6.0)	58.0 (10.25)	58.0 (7.0)	58.0 (9.0)	58.0 (16.0)	58.0 (6.75)	58.0 (7.0)	61.0 (7.0)
Male Gender	163 (77%)	107(73%)	56 (90%) p = 0.005	147 (77%)	16 (84%)	152 (67%)	11 (100%)	148 (67%)	13 (100%) p = 0.04
Diabetes	63 (30%)	47 (32%)	16 (25%)	61 (32%)	2 (11%) p<0.001	62 (31%)	1 (9%)	58 (30%)	5 (39%)
Alcohol etiology	52 (25%)	33 (22%)	19 (31%)	49 (25%)	3 (16%)	51 (25%)	1 (9%)	49 (25%	3 (23%)
MELD score (median, IQR)	11.0 (7.0)	9.0 (5.0)	17.0 (9.25) p<0.0001	10.0 (7.0)	17.0 (8.0) p<0.0001	10.0 (7.0)	22.0 (10.0) p<0.0001	10.0 (7.25)	22.0 (8.0) p<0.0001
Ascites	113 (54%)	73 (49%)	40 (65%) p = 0.05	98 (51%)	15 (79%) p = 0.03	102 (51%)	11 (100%) p = 0.001	101 (52%)	12 (92%)
Prior variceal bleeding	23 (11%)	15 (10%)	8 (13%)	19 (10%)	4 (21%)	20 (10%)	3 (27%)	17 (9%)	6 (48%)
Prior HE	121 (57%)	77 (52%)	44 (71%) p = 0.01	105 (53%)	16 (84%) p = 0.01	112 (66%)	9 (81%)	113 (57%)	8 (62%)
PPI use	97 (46%)	59 (40%)	38 (61%) p = 0.005	87 (45%)	10 (51%)	90 (45%)	7 (64%)	89 (46%)	8 (62%)
Lactulose use	70 (33%)	28 (19%)	42 (72%) p<0.001	55 (30%)	15 (79%) p<0.001	61 (30%)	9 (81%) p = 0.001	60 (31%)	10 (77%) p = 0.001
Rifaximin use	88 (42%)	60 (41%)	28 (45%)	77 (40%)	11 (56%)	84 (42%)	4 (36%)	84 (43%)	4 (31%)
SBP prophylaxis	18 (9%)	7 (5%)	11 (18%) p = 0.002	13 (7%)	5 (27%) p = 0.01	14 (7%)	4 (36%) p = 0.009	16 (9%)	2 (15%)
**Patients who gave urine**	**All patients** **(n = 164)**	**Hospitalization**		**Overt HE**		**Death**		**Transplant**	
		**No** **(n = 108)**	**Yes** **(n = 56)**	**No** **(n = 146)**	**Yes** **(n = 18)**	**No** **(n = 153)**	**Yes** **(n = 11)**	**No** **(n = 152)**	**Yes** **(n = 12)**
Age (median, IQR)	59.0 (7.0)	60.0 (7.0)	58.0 (10.25)	59.0 (7.0)	58.0 (10.5)	59.0 (7.0)	58.0 (16.0)	58.0 (7.25)	61.0 (3.75)
Male Gender	123 (75%)	76 (70%)	47 (84%)	107 (73%)	16 (89%)	112 (73%)	11 (100%)	111 (73%)	12 (100%) p = 0.03
Diabetes	54 (33%)	37 (34%)	17 (25%)	52 (36%)	2 (11%)	51 (33%)	3 (27%)	50 (35%)	4 (33%)
Alcohol etiology	42 (26%)	27 (25%)	17 (25%)	39 (27%)	3 (17%)	41 (27%)	1 (10%)	38 (25%)	3 (25%)
MELD score (median, IQR)	11.0 (9.0)	9.0 (5.0)	17.0 (9.25) p<0.0001	10.0 (9.0)	21.0 (5.5) p<0.0001	11.0 (8.0)	22.0 (10.0) p<0.0001	10.0 (8.0)	21.0 (5.5) p<0.0001
Ascites	78 (47%)	42 (43%)	36 (64%) p = 0.01	68 (47%)	10 (56%)	68 (44%)	10 (91%) p = 0.003	68 (45%)	10 (89%) p = 0.01
Prior variceal bleeding	20 (12%)	14(13%)	6 (10%)	17 (12%)	3 (17%)	17 (11%)	3 (27%)	15 (10%)	5 (41%)
Prior HE	84 (51%)	45 (42%)	39 (70%) p = 0.001	68 (47%)	16(89%) p = 0.03	75 (49%)	9 (89%) p = 0.05	77 (53%)	7 (60%)
PPI use	87 (53%)	56 (52%)	31 (55%)	78 (53%)	9 (50%)	80 (52%)	7 (64%)	79 (52%	8 (67%)
Lactulose use	66 (40%)	28 (26%)	38 (69%) p<0.001	51 (35%)	15(83%) p = 0.006	57 (37%)	9 (89%)	57 (38%)	9 (75%)
Rifaximin use	54 (33%)	31 (29%)	22 (39%)	43 (30%)	11(62%) p = 0.006	50 (32%)	4 (37%)	50 (33%))	4 (33%)
SBP prophylaxis	19 (12%)	6 (6%)	13 (23%) p = 0.001	14 (10%)	5 (28%) p = 0.03	15 (10%)	4 (37%) p = 0.02	16 (11%)	3 (25%)

Data in median (IQR) or in raw numbers (%); Significant p-values on Mann-Whitney test, Fisher exact or Chi-square test as appropriate are shown in each box in those who developed that outcome compared to those who did not.

### Metabolomics ChemRICH analysis (S1, S2, S3 and S4 Tables)

#### Hospitalizations (Fig 1)

**Fig 1:**
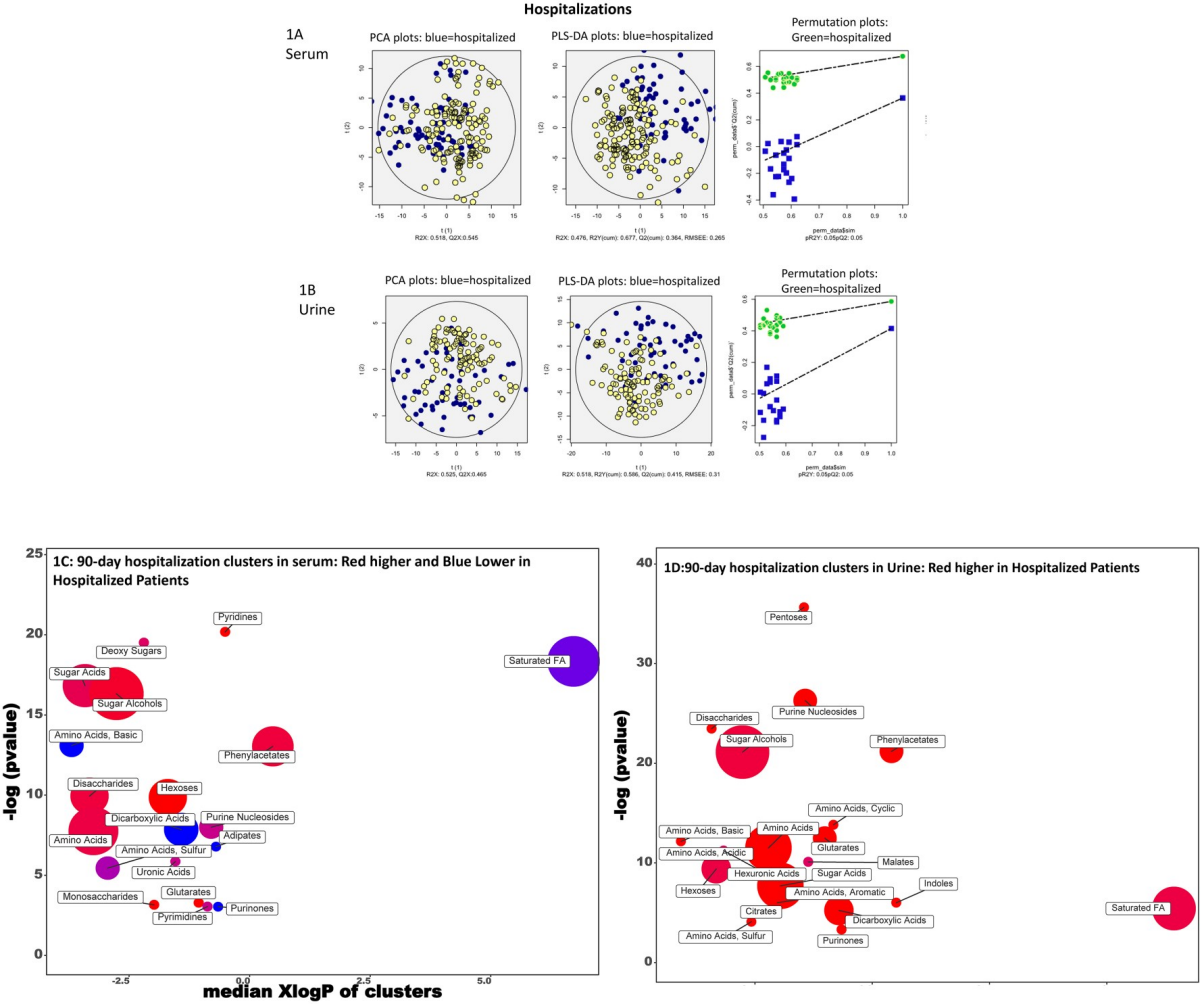
90-day hospitalizations. A: Serum PCA/PLSDA and permutation plots PCA showing visual separation between those who were hospitalized (blue) vs the rest (yellow dots), PLS-DA showing visual separation between those who were hospitalized (blue) vs those who were not (yellow dots) and Permutation test plots indicating the validation of the PLS-DA models with visual separation between hospitalized (green) versus not hospitalized (blue). B: Urine PCA/PLSDA and permutation plots PCA showing visual separation between those who were hospitalized (blue) vs the rest (yellow dots), PLS-DA showing visual separation between those who were hospitalized (blue) vs those who were not (yellow dots) and Permutation test plots indicating the validation of the PLS-DA models with visual separation between hospitalized (green) versus not hospitalized (blue). C: ChemRICH analysis of serum. Red clusters associated with higher and blue one associated with lower outcomes. D. ChemRICH analysis of urine. Red clusters associated with higher outcomes.

Serum metabolite clusters increased in patients who were hospitalized were pyridines and pyrimidines, sugar acids, sugar alcohols, amino acids and sulfur amino acids, phenylacetates, disaccharides, monosaccharides, hexoses, uronic acid and glutarates. The following were decreased in those who were hospitalized; basic amino acids, saturated fatty acids, dicarboxylic acids, purinones and adipates. In the urine, clusters related to pentoses, purines, disaccharides, phenylacetates, sugar alcohols, amino acids (cyclic, basic, acidic, sulfur and aromatic), glutarates, hexuronates, malates, hexoses, sugar acids, citrates, indoles, saturated FA, dicarboxylates, purinones were associated with hospitalizations.

#### HE (Fig 2)

**Fig 2:**
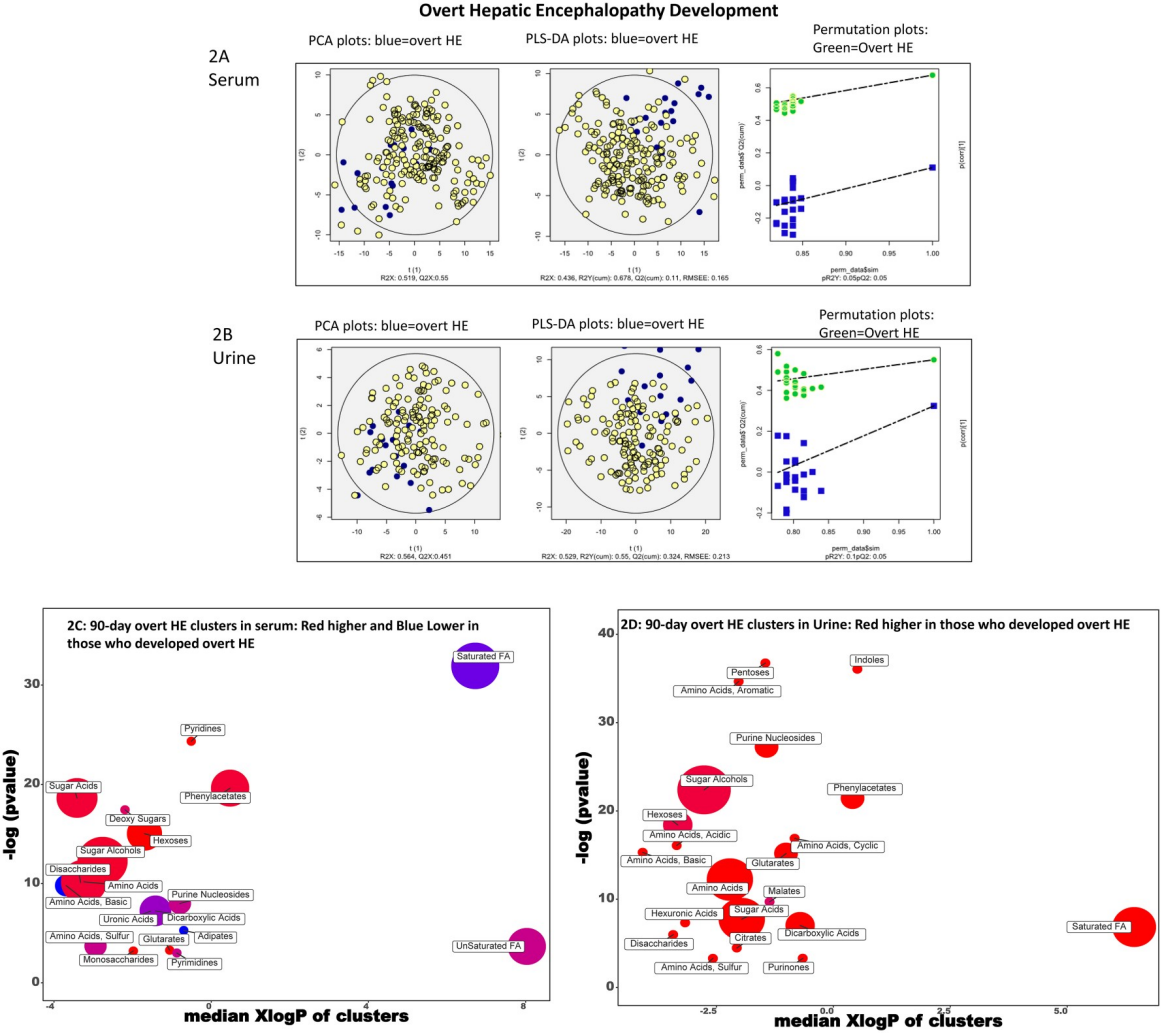
90-day hepatic encephalopathy (HE) development. A: Serum PCA/PLSDA and permutation plots PCA showing visual separation between those who developed HE (blue) vs the rest (yellow dots), PLS-DA showing visual separation between those who developed HE (blue) vs those who did not (yellow dots) and Permutation test plots indicating the validation of the PLS-DA models with visual separation between HE (green) versus no HE (blue). B: Urine PCA/PLSDA and permutation plots PCA showing visual separation between those who developed HE (blue) vs the rest (yellow dots), PLS-DA showing visual separation between those who developed HE (blue) vs those who did not (yellow dots) and Permutation test plots indicating the validation of the PLS-DA models with visual separation between HE (green) versus no HE (blue). C: ChemRICH analysis of serum. Red clusters associated with higher and blue one associated with lower outcomes D. ChemRICH analysis of urine. Red clusters associated with higher outcomes.

Patients who developed HE demonstrated higher serum clusters related to pyridines and pyrimidines, sugar acids, sugar alcohols, amino acids, phenylacetates, disaccharides, monosaccharides, hexoses, uronic acid and glutarates while basic amino acids, saturated fatty acids, dicarboxylic acids, purinones and adipates were lower. In the urine, higher clusters related to pentoses, purines, disaccharides, phenylacetates, sugar alcohols, amino acids (Cyclic, basic, acidic, sulfur and aromatic), glutarates, hexuronates, malates, hexoses, sugar acids, citrates, indoles, saturated FA, dicarboxylates, purinones were associated with HE.

#### Death (Fig 3)

**Fig 3:**
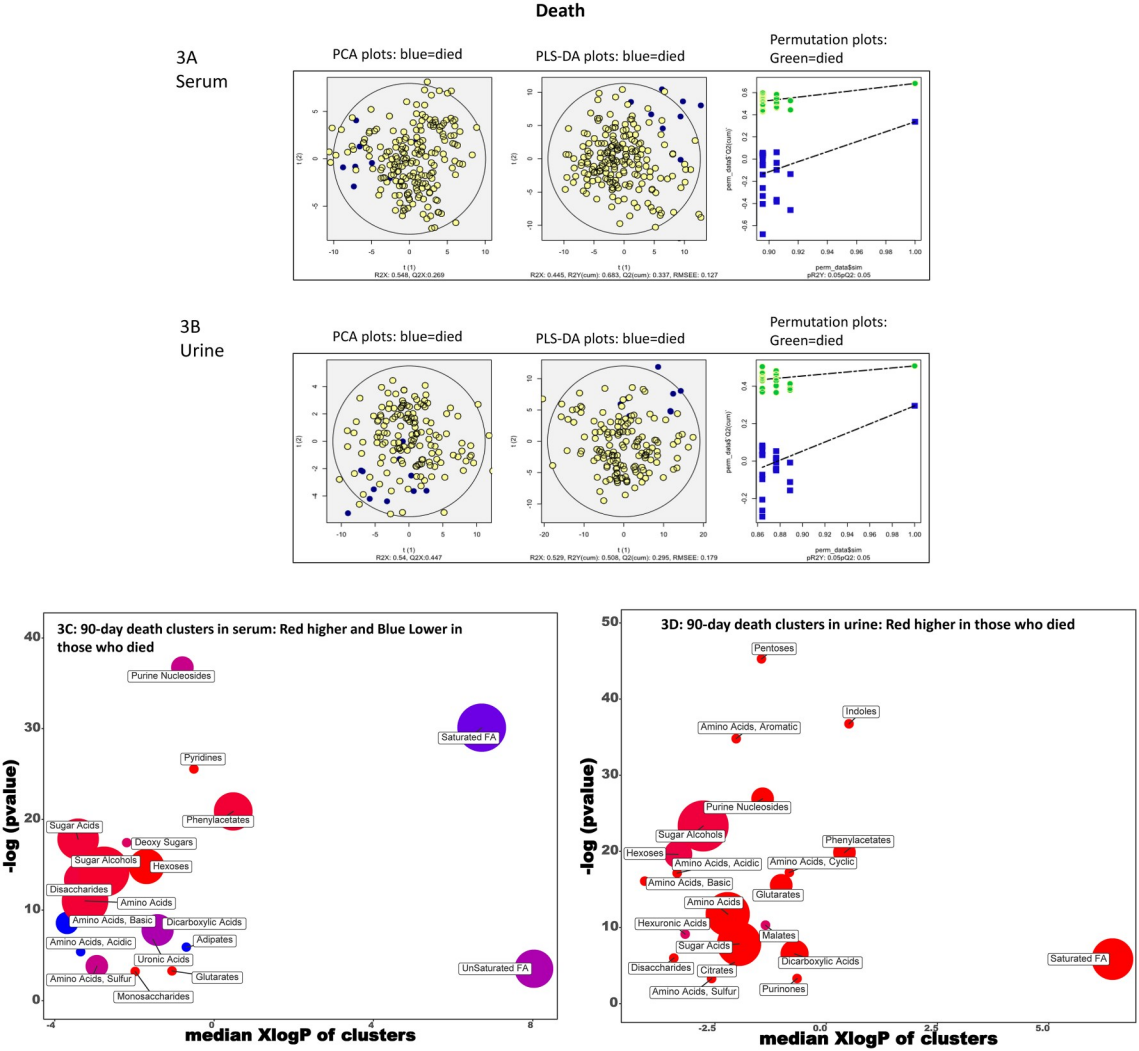
90-day death. A: Serum PCA/PLSDA and permutation plots PCA showing visual separation between those who died (blue) vs the rest (yellow dots), PLS-DA showing visual separation between those who died (blue) vs those who did not (yellow dots) and Permutation test plots indicating the validation of the PLS-DA models with visual separation between those who died (green) versus not (blue). B: Urine PCA/PLSDA and permutation plots PCA showing visual separation between those who died (blue) vs the rest (yellow dots), PLS-DA showing visual separation between those who died (blue) vs those who did not (yellow dots) and Permutation test plots indicating the validation of the PLS-DA models with visual separation between those who died (green) versus not (blue). C: ChemRICH analysis of serum. Red clusters associated with higher and blue one associated with lower outcomes. D. ChemRICH analysis of urine. Red clusters associated with higher outcomes.

In the serum, pyridines, sugar acids, sugar alcohols, acidic amino acids, phenylacetates, disaccharides, monosaccharides, hexoses, and glutarates were higher in those who died while saturated and unsaturated fatty acids, purine nucleosides, basic and sulfur amino acids, dicarboxylic acids, and adipates were lower. In the urine, metabolites related to saturated FAs, pentoses, Indoles, amino acids (basic, cyclic, acidic and basic), purines malates, disaccharides, dicarboxylic acids, hexuronic acids, hexoses, phenylacetate and citrates were associated with higher death.

#### Transplant (Fig 4)

**Fig 4:**
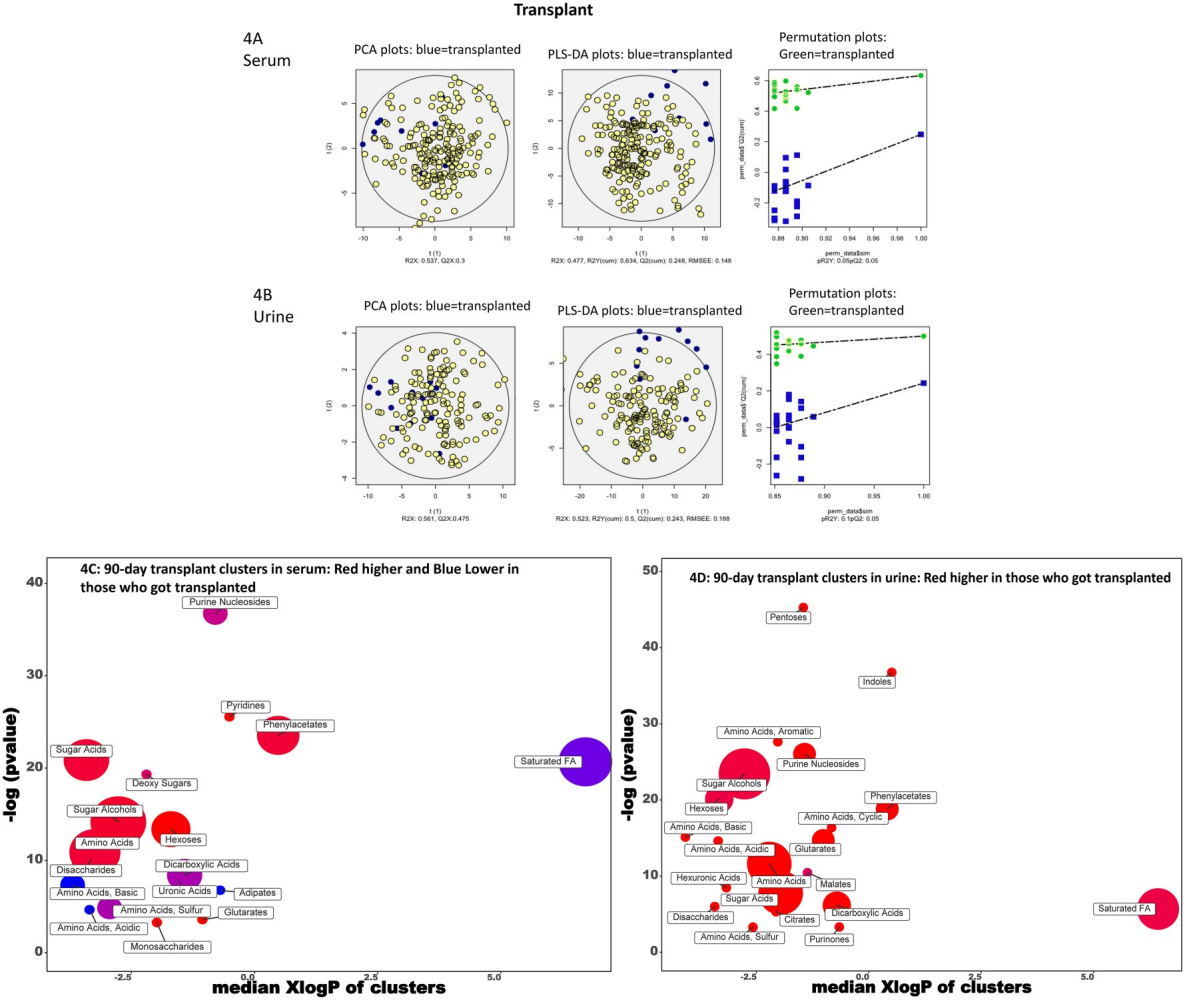
90-day transplant. A: Serum PCA/PLSDA and permutation plots PCA showing visual separation between those who received a transplant (blue) vs the rest (yellow dots), PLS-DA showing visual separation between those who died (blue) vs those who did not (yellow dots) and Permutation test plots indicating the validation of the PLS-DA models with visual separation between those who received a transplant (green) versus not (blue). B: Urine PCA/PLSDA and permutation plots PCA showing visual separation between those who received a transplant (blue) vs the rest (yellow dots), PLS-DA showing visual separation between those who received a transplant (blue) vs those who did not (yellow dots) and Permutation test plots indicating the validation of the PLS-DA models with visual separation between those who received a transplant (green) versus not (blue). C: ChemRICH analysis of serum. Red clusters associated with higher and blue one associated with lower outcomes. D. ChemRICH analysis of urine. Red clusters associated with higher outcomes.

Patient who received a transplant demonstrated again a similar pattern to those who died with higher serum pyridines, sugar acids, sugar alcohols, phenylacetates, disaccharides, monosaccharides, hexoses, and glutarates and lower serum saturated fatty acids, purine nucleosides, basic, acidic and sulfur amino acids, dicarboxylic acids, and adipates in those who were not transplanted. In the urine again, there were higher metabolites related to saturated FAs, pentoses, indoles, amino acids, purines, malates, disaccharides, dicarboxylic acids, hexuronic acids, hexoses, phenylacetate and citrates in those who got transplanted.

#### Metabolomics

On logistic regression after adjustment for age, gender, diabetes, prior HE, medication use and controlling for FDR we found that for 90-day hospitalization there were 290 urinary and 232 serum metabolites that were significant, for HE there were 284 urinary and 228 serum metabolites while 284 serum and 227 urine metabolites were significant for death. 285 urine and 229 serum metabolites were significant for transplant. The specific metabolites are shown in S5 to S12 Tables.

#### PCA and PLS-DA

Using the variables significant on logistic regression, the PCA and PLS-DA and permutation analyses showed visual separation between patients who developed HE, needed transplant, required hospitalization or died (Figs 1A/1B, 2A/2B, 3A/3B and 4A/4B) for urine and serum metabolites. MetaMaPP changes are shown in S1–S8 Figs for each fluid and specific outcomes.

#### VIP results

Tables 2 and 3 show top 20 metabolites that was associated for each outcome in serum and urine. The entire dataset is in S13 and S14 Tables.

**Table 2:** Top 20 individual serum metabolites with the highest VIP scores for all outcomes.

Death	VIP	Overt HE	VIP	Transplant	VIP	Hospitalization	VIP
hypoxanthine mix spec with ornithine	1.691674911	sophorose	1.761664709	N-acetyl-D-tryptophan minor2	2.077881	2-deoxyerythritol NIST	1.743146383
oxoproline	1.6136051	**leucine**	1.632356319	oxoproline	1.811711	conduritol betat epoxide minor	1.735224901
2-hydroxybutanoic acid	1.547067628	oxoproline	1.600209198	caprylic acid	1.571073	arabitol	1.735079543
aconitic acid	1.532266306	**isoleucine**	1.582285313	xylulose NIST	1.525188	3-phenyllactic acid	1.582691715
1-deoxyerythritol	1.479701386	stearic acid	1.543379415	stearic acid	1.506294	**valine**	1.564061841
urea	1.467688896	glycine	1.533397167	ornithine	1.436212	tocopherol alpha	1.558857206
heptadecanoic acid NIST	1.467584884	**valine**	1.474016441	malic acid	1.428341	threitol 2	1.536478704
palmitic acid	1.465626513	icosenoic acid	1.45732143	asparagine 2TMS minor	1.41985	alpha ketoglutaric acid	1.507600006
linoleic acid	1.439986096	heptadecanoic acid NIST	1.44794942	2-deoxyerythritol NIST	1.415952	pseudo uridine	1.490157196
oleic acid	1.419174514	3-hydroxybutanoic acid mix spec	1.439422872	dodecanol	1.40343	2-deoxyerythritol	1.415289639
3-hydroxybutanoic acid mix spec	1.416205224	palmitic acid	1.439262932	trans-4-hydroxyproline	1.394916	phenylethylamine	1.406504891
galacturonic acid 2	1.415715043	linolenic acid	1.415766801	tyrosine minor	1.394313	ethanolamine	1.404227462
glucose 2	1.409341965	linoleic acid	1.414421309	2-hydroxybutanoic acid	1.392622	**leucine**	1.40017147
1-methyladenosine	1.39527319	xylitol	1.401049684	isolinoleic acid NIST	1.351142	2-ketoadipic acid	1.378520717
**isoleucine**	1.388507228	oleic acid	1.389973739	N-acetylglutamate	1.336039	N-acetyl-D-hexosamine	1.376185515
**leucine**	1.3862066	2-hydroxybutanoic acid	1.386819203	glycine TMS1x	1.332613	inulobiose 2	1.351429174
phthalic acid	1.381706001	glutamine	1.381953827	adipic acid	1.332586	maltose 1	1.341200867
**valine**	1.367678245	1-deoxyerythritol	1.356001613	succinic acid	1.326415	**isoleucine**	1.331328379
1,5-anhydroglucitol	1.360892224	methylhexadecanoic acid	1.344876304	threitol 2	1.323956	aspartic acid	1.330337414
glucuronic acid mix spec	1.360625469	tocopherol alpha	1.338038687	glutamine	1.296891	3-methoxytyrosine NIST	1.328860561

**Table 3:** Top 20 individual urine metabolites with the highest VIP scores for all outcomes.

Death	VIP	Overt HE	VIP	Transplant	VIP	Hospitalizations	VIP
3-aminoisobutyric acid	1.643070115	xylitol	1.872164501	phosphoric acid.1	1.725698799	glycolic acid	1.827212618
2,3-dihydroxybutanoic acid NIST	1.614889928	5-methoxytryptamine	1.867140102	2-hydroxyvaleric acid	1.701237906	3-hydroxypropionic acid	1.7560861
2-hydroxy-2-methylbutanoic acid	1.610992622	citric acid	1.805605851	2-deoxytetronic acid NIST	1.692077564	2-hydroxyvaleric acid	1.584754621
3-ureidopropionate	1.605150109	creatinine	1.741549209	butane-2,3-diol (NIST)	1.650237473	butane-2,3-diol (NIST)	1.573148827
creatinine	1.596144716	2-hydroxy-2-methylbutanoic acid	1.738767214	serine minor	1.630710133	creatinine	1.55118776
2-hydroxyvaleric acid	1.54878599	2-hydroxyvaleric acid	1.52753461	leucine	1.625056096	2,3-dihydroxybutanoic acid NIST	1.545142682
5-methoxytryptamine	1.528630586	glutamine	1.505993268	adipic acid	1.624222805	fructose 2	1.542568903
uracil	1.482652188	glycolic acid	1.45153304	creatinine	1.61593923	glycine	1.530902016
glycine TMS1x	1.478569024	dodecane	1.414469964	oxalic acid	1.560206429	glycine TMS1x	1.491975324
proline	1.451424687	azelaic acid	1.414292736	2-hydroxy-2-methylbutanoic acid	1.498640399	azelaic acid	1.411987043
glycolic acid	1.443983922	histidine	1.403092474	glycine TMS1x	1.46031286	fructose 1	1.409805348
xylitol	1.399886017	threitol 2	1.40223294	indole-3-lactate	1.460261311	2-hydroxy-2-methylbutanoic acid	1.397753125
3-hydroxypropionic acid	1.397233101	UDP-glucuronic acid	1.398986905	3-hydroxybutanoic acid	1.443439445	xylulose NIST	1.391645123
butane-2,3-diol (NIST)	1.391963441	N-methylalanine	1.386943204	isorhamnose	1.420245818	benzoic acid mix spec	1.38144168
pelargonic acid	1.370941662	glycine TMS1x	1.382226302	benzoic acid mix spec	1.408113448	cyclohexylamine NIST	1.342356615
benzoic acid mix spec	1.352670887	2,3-dihydroxybutanoic acid NIST	1.380952853	3-ureidopropionate	1.407285453	threitol 2	1.32700058
2-deoxyerythritol NIST	1.342240486	butane-2,3-diol (NIST)	1.367090966	galacturonic acid	1.386165728	dodecane	1.304573934
citric acid	1.340997281	shikimic acid	1.349692331	glycolic acid	1.381902268	uracil	1.285124236
shikimic acid	1.332763509	inulotriose 1	1.34653941	dodecane	1.315481375	stearic acid	1.281632065

#### Serum ROC results (Fig 5)

**Fig 5:**
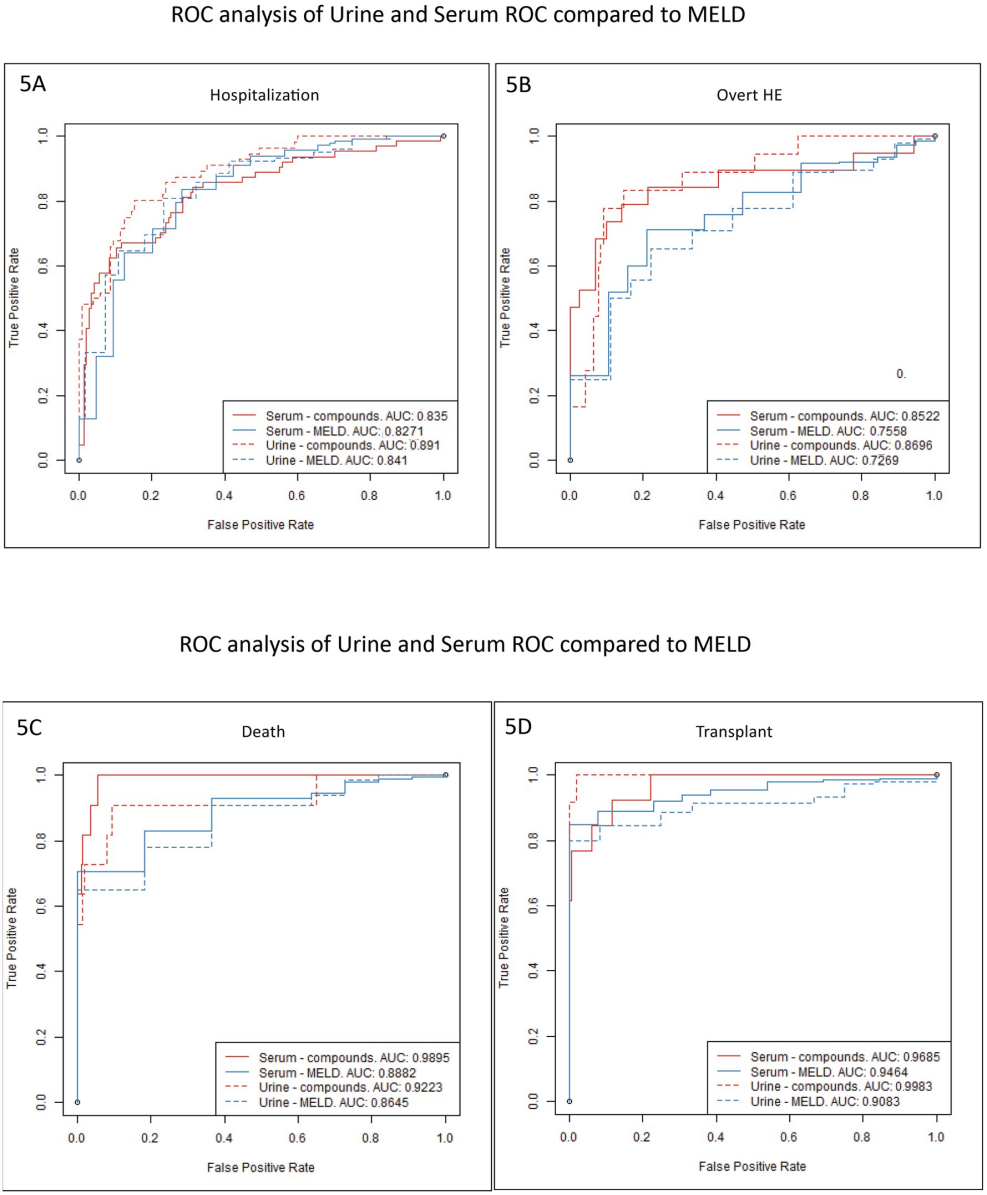
Receiver operating characteristic curve analysis of serum and urine metabolites compared to MELD score. Solid red: adjusted serum metabolites, dashed red: MELD score for patients who gave serum, solid blue: adjusted urine metabolites, dashed blue: MELD score for those who gave urine. Areas under the curve are noted underneath the specific figures A: 90-day hospitalizations, B: 90-day overt HE episodes, C: 90-day death, D: 90-day transplant.

AUC for metabolites was 0.99 compared to 0.88 MELD score for death. Serum metabolites for overt HE had an AUC of 0.85 compared to MELD with an AUC of 0.75. AUC for transplants were higher in serum metabolites 0.97 vs 0.94 for MELD score. 90-day hospitalization AUCs were similarly higher with serum metabolites 0.84 vs 0.83 on MELD alone. Of these comparisons, prediction of death and overt HE was statistically higher for metabolites compared to MELD (p = 0.03 and p = 0.05 respectively).

#### Urine ROC results (Fig 5)

90-day death AUC for metabolites was 0.92 compared to 0.86 for MELD score. For overt HE episodes also the urine metabolites AUC was higher 0.87 vs MELD at 0.72. Urine metabolite prediction for transplants was 0.99 compared to 0.90 for MELD score. An AUC of 0.89 vs 0.84 for hospitalizations was seen with metabolites compared to MELD alone. Of these comparisons, prediction of transplant was statistically significant with metabolites (p = 0.001) with a trend towards better hospitalization prediction (p = 0.058). Therefore, serum and urine metabolites had higher AUC compared to MELD on all outcomes. In addition, serum metabolites were better predictors than urine for 90-day overt HE and death while the reverse was true for transplant and hospitalizations.

## Discussion

The current exploratory study found that specific patterns of changes in serum and urine metabolites were associated with prediction of clinically relevant outcomes centered on hepatic encephalopathy, hospitalizations, death and transplant. Metabolites linked with changes in lipid, amino-acid and bioenergetics metabolism were associated with the development of these complications. The adjusted metabolites also suggested an improved predictability compared to MELD score.

Changes in metabolomics in cirrhosis are important to analyze due to the major role of the liver in several important metabolic processes[11]. These span amino acid, lipid and energy metabolism that have the potential to create serum and urine biomarkers and provide pathophysiological insight into the disease process. Development of HE is clinically relevant and was the leading cause of hospitalization in our population[2]. HE has emerged as the leading reason for readmissions and the potential pathophysiology and clinical prediction of this outcome is very important[17, 18]. Saturated serum FAs (caprylic, arachidic, lauric, stearic etc.) were associated with lower while FAs belonging to the eicosanoid pathway (iso-linoleic and icosenoic acids) were associated with greater HE. This is interesting because rifaximin therapy, which is usually protective against overt HE, is associated with higher serum saturated medium and long chain fatty acids[19]. Saturated fatty acids have also been associated with a lower liver injury due to alcohol, which may a role in this relative protection[20]. On the other hand, branched chain amino acids typically associated with lower ammonia production, and glutamine, that represents ammonia capture with glutamate were protective[21, 22]. Benzoic acid was associated with lower HE development and is likely related to gut microbial changes associated with cirrhosis, which can influence hippurate formation[23]. Also supporting the HE-related systemic milieu, there was higher inositol which is extruded from astrocytes after ammonia influx, and aromatic amino acid metabolites such as phenylacetates, phenyl-lactates, indoles, and urea cycle intermediates that were higher in those who developed overt HE[24, 25]. Lactic acid and high free sugars and sugar alcohols were also associated with minimal HE in prior serum studies using nuclear magnetic resonance (NMR)[26]. Our findings extend these using GC/MS spectroscopy and link these to outcome development in this exploratory experience. Since HE was the major cause of hospitalization, the majority of the metabolomic findings were similar in pattern and predictive capability for that outcome as well.

Using individual VIP analyses, similar changes were noted above using CHEMRICH. Urinary and serum metabolites that were consistently in the top twenty by VIP were related to hippurate metabolism (benzoic acid and glycine). Glycine is associated with several important metabolites that were also highly represented in urine and serum, which are creatinine, and metabolites required for glutathione formation. These were glycine, glutamic acid and 2-Hydroxybutyric acid, which are involved in glutathione formation, an important hepato-protective metabolite[27]. There was also a contribution of urea cycle intermediates (ornithine, urea, aspartate) and products of ammonia metabolism such as glutamine and aspartate in the serum. In addition, butanoate or butyric acid metabolites and propionate metabolites, which are major short-chain fatty acids, were also found to be predictive of outcomes[28]. Lastly, again long-to medium-chain fatty acids and branched chain amino acids, valine, leucine and isoleucine, were consistently represented among the serum metabolites.

The prediction of outcomes in cirrhosis is challenging given the multiple competing factors related to prior complications, cirrhosis severity, etiology, demographics and medications[3]. We demonstrated in this initial experience that serum and urine metabolomics were complementary and tended to be better than the MELD score alone in predicting transplant, hospitalizations, death and HE. This is important because these could be prevented or anticipated if their occurrence can be more reliably predicted[29]. We found that the relative predictive capability of the urine was greater than serum for transplant and hospitalizations while serum was better than urine for HE and prediction of death. Prior studies have been performed in decompensated cirrhosis and plasma metabolomics by McPhail et al and Mindikoglu et al[30, 31]. They demonstrated excellent predictive capability for death using multiple metabolomic platforms in decompensated cirrhosis and focused in those with kidney dysfunction and hepatorenal syndrome. Our experience extends this by including compensated and decompensated cirrhosis, patients with and without pre-existing kidney disease, analyzing both serum and urine, and analyzing the pattern of change of metabolomics associated with other outcomes as well. Most of the complications showed a statistically significant or a trend towards better prediction with metabolites compared to MELD. These may expand the generalizability of these findings in a more general outpatient cirrhosis population once validated in other cohorts.

It is interesting that similar groups of serum and urinary metabolites could predict the major complications regardless of the specific outcome and remained better than MELD despite adjusting for clinical indices and several medications. The 90-day interval was chosen because this is the validity period of the MELD score and also to reflect prior studies on readmissions in this population[32]. The importance of this additive component to the MELD score using metabolomics reflects other potential biomarkers of disease severity such as minimal/covert HE, sarcopenia or microbiota that are not captured by MELD[7, 33–37]. Samples were collected from cirrhotic outpatients, who already have a skewed metabolic baseline. Therefore, despite controlling for clinically relevant variables and in this skewed background, we were able to define added value of these metabolites in both fluids with trends or with statistical significance. However, the need to develop better biomarkers is even greater in the more complex hospitalized cirrhotic patients[38], where better predictors for acute-on-chronic liver failure are needed. There is a call for hybrid clinical and biological markers for improving prognostication in this population[39].

We recognize that although it may improve prognostication, it is not viable to routinely perform metabolomics in the clinic. Therefore, these results are the initial experience and need to be further validated. However, the demonstration of these alterations, we believe may help focus on specific metabolite patterns that could be narrowed down in the future to potentially add to our current clinical biomarkers. Our study is also limited using only the GC/MS platform and it is likely that use of NMR and lipidomics could have further improved the prognostication. Since many outcomes follow one another, we found similar groups of metabolites that were associated with these predictions. Therefore, our analysis also focused on HE and hospitalizations, which precede transplant and death. This could also reflect the altered metabolomic milieu in advancing stages of liver disease, rather than be focused on specific complications. Due to the relatively lower number of non-OHE hospitalizations, we did not perform a subgroup analysis of hospitalizations that were related to hepato-renal syndrome, hyponatremia, infections and liver-unrelated reasons. Not all patients provided urine, the majority due to dialysis, which skewed the potential prediction towards transplant in the serum providers since serum was collected from every patient. This may also be the reason for better differentiation between MELD score and transplant prediction in those who gave urine since this only included patients not on dialysis. Due to the relatively short follow-up, we did not perform a time-to-event and associated competing-risks analysis.

We conclude in this exploratory study, that there are major alterations in serum and urinary metabolomics focused on lipid, amino-acid and bioenergetic metabolism that are associated with development of overt HE, hospitalization, transplant and death over 90 days. Glycine metabolism intermediates, urea cycle intermediates, branched chain amino acids and long to medium chain fatty acids should form the focus of future directed metabolomic strategies. Further validation of these results is needed in larger multi-center studies to determine the utility of hybrid scores combining biomarkers with clinical variables for predicting outcomes in cirrhosis.

## Supporting information

S1 Methods (DOCX)

S1 Fig MetaMapp in serum for hospitalizations. Red nodes = ↑ in those with outcomes, Blue nodes = ↓ in those with outcomes, Yellow nodes = not significant, Red edges: KEGG similarity and Blue edges: Tanimoto chemical similarity.
(TIF)

S2 Fig MetaMapp in urine for hospitalizations. Red nodes = ↑ in those with outcomes, Blue nodes = ↓ in those with outcomes, Yellow nodes = not significant, Red edges: KEGG similarity and Blue edges: Tanimoto chemical.
(TIF)

S3 Fig MetaMapp in serum for HE development. Red nodes = ↑ in those with outcomes, Blue nodes = ↓ in those with outcomes, Yellow nodes = not significant, Red edges: KEGG similarity and Blue edges: Tanimoto chemical.
(TIF)

S4 Fig MetaMapp in urine for HE development. Red nodes = ↑ in those with outcomes, Blue nodes = ↓ in those with outcomes, Yellow nodes = not significant, Red edges: KEGG similarity and Blue edges: Tanimoto chemical.
(TIF)

S5 Fig MetaMapp in serum for death. Red nodes = ↑ in those with outcomes, Blue nodes = ↓ in those with outcomes, Yellow nodes = not significant, Red edges: KEGG similarity and Blue edges: Tanimoto chemical.
(TIF)

S6 Fig MetaMapp in urine for death. Red nodes = ↑ in those with outcomes, Blue nodes = ↓ in those with outcomes, Yellow nodes = not significant, Red edges: KEGG similarity and Blue edges: Tanimoto chemical.
(TIF)

S7 Fig MetaMapp in serum for transplant. Red nodes = ↑ in those with outcomes, Blue nodes = ↓ in those with outcomes, Yellow nodes = not significant, Red edges: KEGG similarity and Blue edges: Tanimoto chemical.
(TIF)

S8 Fig MetaMapp in urine for transplant. Red nodes = ↑ in those with outcomes, Blue nodes = ↓ in those with outcomes, Yellow nodes = not significant, Red edges: KEGG similarity and Blue edges: Tanimoto chemical.
(TIF)

S1 Table ChemRICH overt HE prediction. (DOCX)

S2 Table ChemRICH hospitalization prediction. (DOCX)

S3 Table ChemRICH death prediction. (DOCX)

S4 Table ChemRICH transplant prediction. (DOCX)

S5 Table Logistic regression of serum metabolites with 90-day hospitalizations. (DOCX)

S6 Table Logistic regression of urine metabolites with 90-day hospitalizations. (DOCX)

S7 Table Logistic regression serum 90 day HE development. (DOCX)

S8 Table Logistic regression urine 90 day HE development. (DOCX)

S9 Table Logistic regression serum 90 day death. (DOCX)

S10 Table Logistic regression urine 90 day death. (DOCX)

S11 Table Logistic regression serum 90 day transplant. (DOCX)

S12 Table Logistic regression urine 90 day transplant. (DOCX)

S13 Table Serum VIP scores for individual named metabolites for each complication arranged by VIP score. (DOCX)

S14 Table Urine VIP scores for individual named metabolites for each complication arranged by VIP score. (DOCX)
