# Supplementary material for: Serum and urinary metabolomics and outcomes in cirrhosis
Source: PLoS One. 2019 Sep 27;14(9):e0223061. doi: 10.1371/journal.pone.0223061 (PMC6764675; doi:10.1371/journal.pone.0223061)
Supplement: S1 Methods — (DOCX) [file pone.0223061.s001.docx]

**Supplementary Methods:**

Briefly, 30 μl samples were extracted using 1 mL of a -20°C cold, degassed mixture of acetonitrile/isopropanol/water (3:3:2), dried down and derivatized with methoximation and trimethylsilylation. Data were acquired on a Leco Pegasus IV time of flight mass spectrometer (Leco, St. Joseph, MI) at 17 Hz and -70 eV ionization energy from 85-500 Da mass range at 1800 V detector voltage. 0.5 μl samples were injected into a multi-baffled glass liner using an injection temperature ramping from 50 - 250°C with 25 second splitless time. Chromatography was performed at 1 mL Helium flow/min on a 30 m rtx5Sil-MS column, 0.25 mm i.d. and 0.25 μm film (Restek, Bellefonte, PA). Data were processed on ChromaTOF 4.0 (Leco, St. Joseph, MI) and the BinBase database system.
