# Supplementary figures and images for: Serum and urinary metabolomics and outcomes in cirrhosis
Source: PLoS One. 2019 Sep 27;14(9):e0223061. doi: 10.1371/journal.pone.0223061 (PMC6764675; doi:10.1371/journal.pone.0223061)

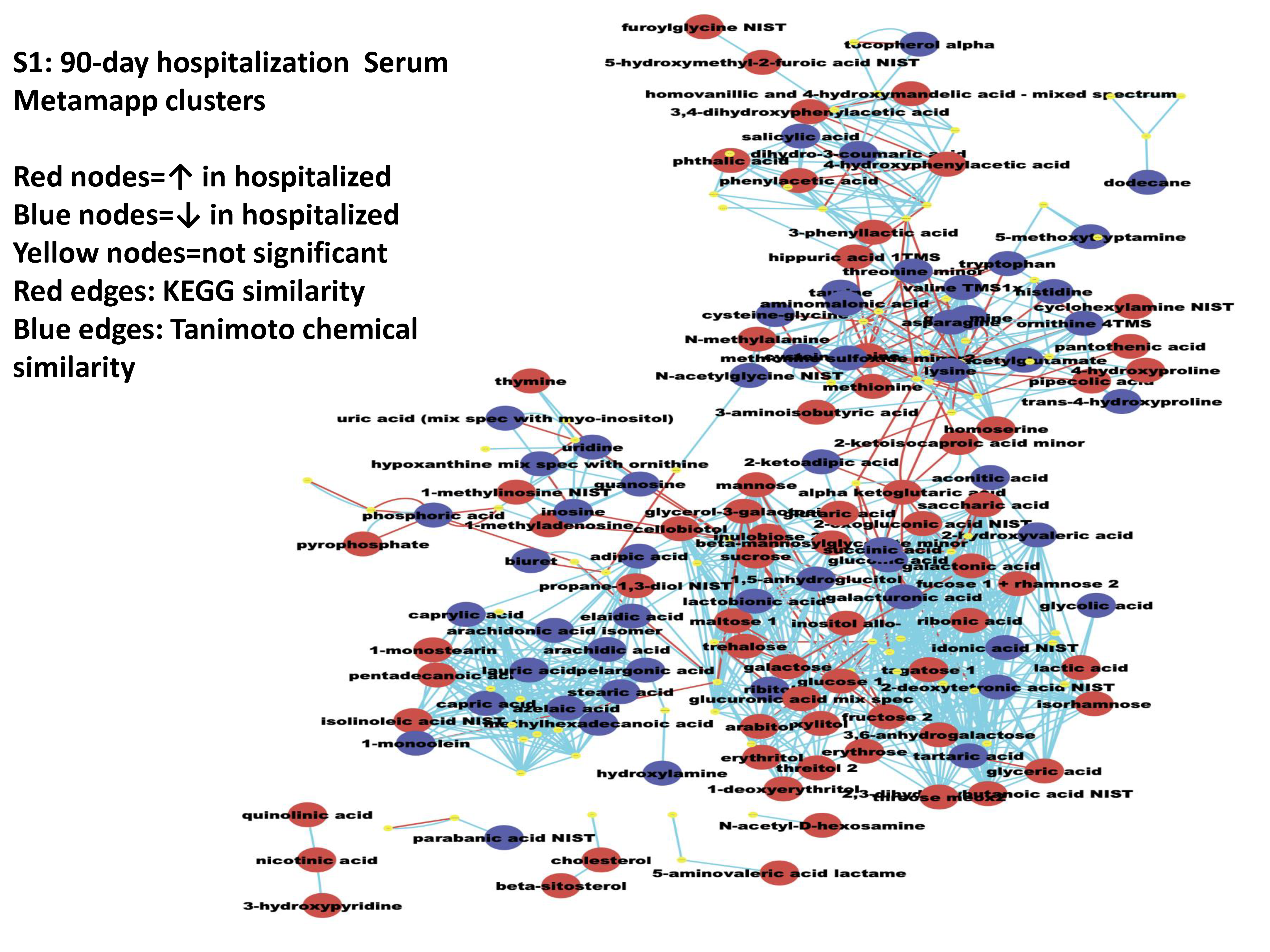

Supplement: S1 Fig — Red nodes = ↑ in those with outcomes, Blue nodes = ↓ in those with outcomes, Yellow nodes = not significant, Red edges: KEGG similarity and Blue edges: Tanimoto chemical similarity. (TIF) [file pone.0223061.s002.tif]

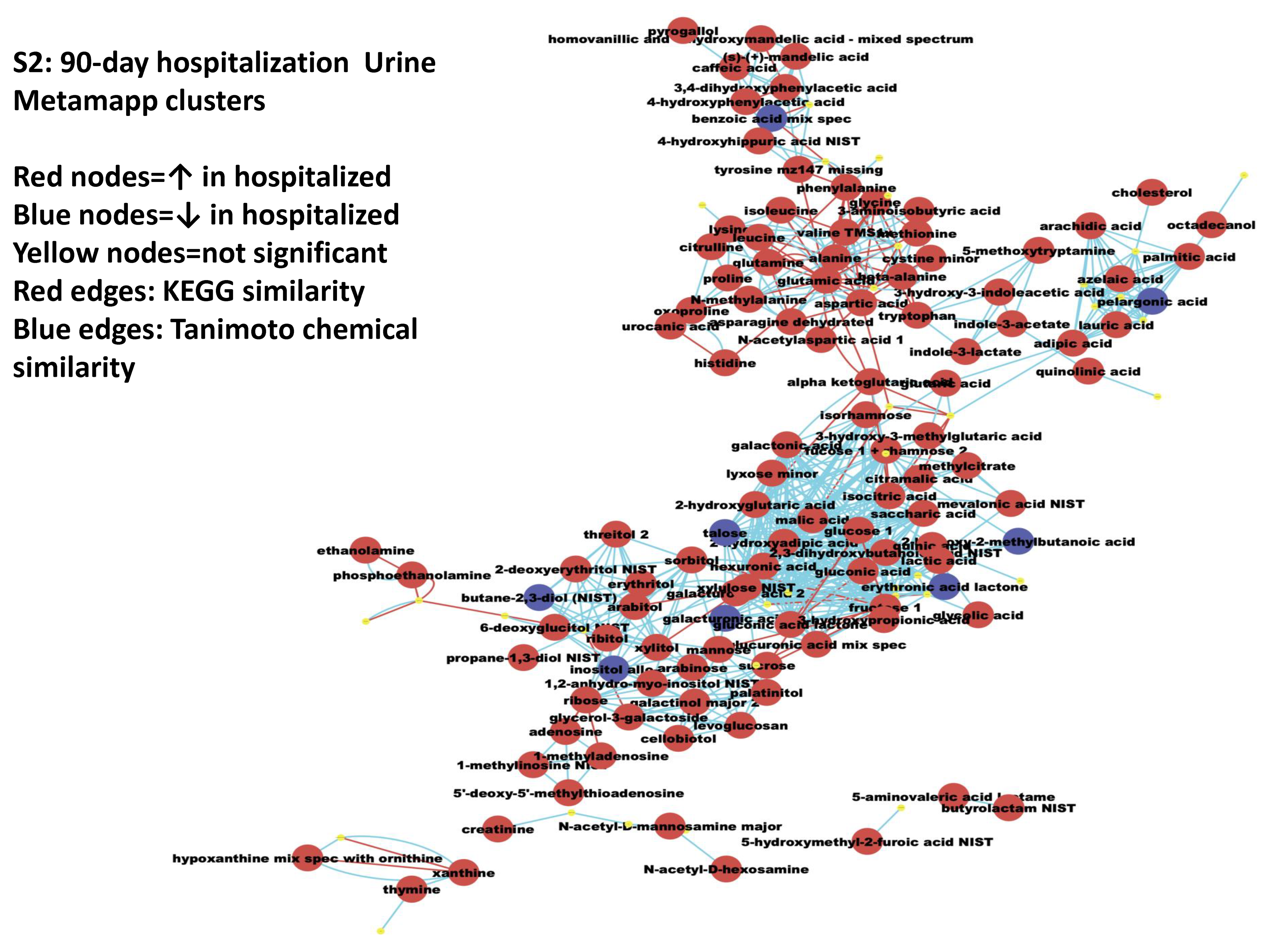

Supplement: S2 Fig — Red nodes = ↑ in those with outcomes, Blue nodes = ↓ in those with outcomes, Yellow nodes = not significant, Red edges: KEGG similarity and Blue edges: Tanimoto chemical. (TIF) [file pone.0223061.s003.tif]

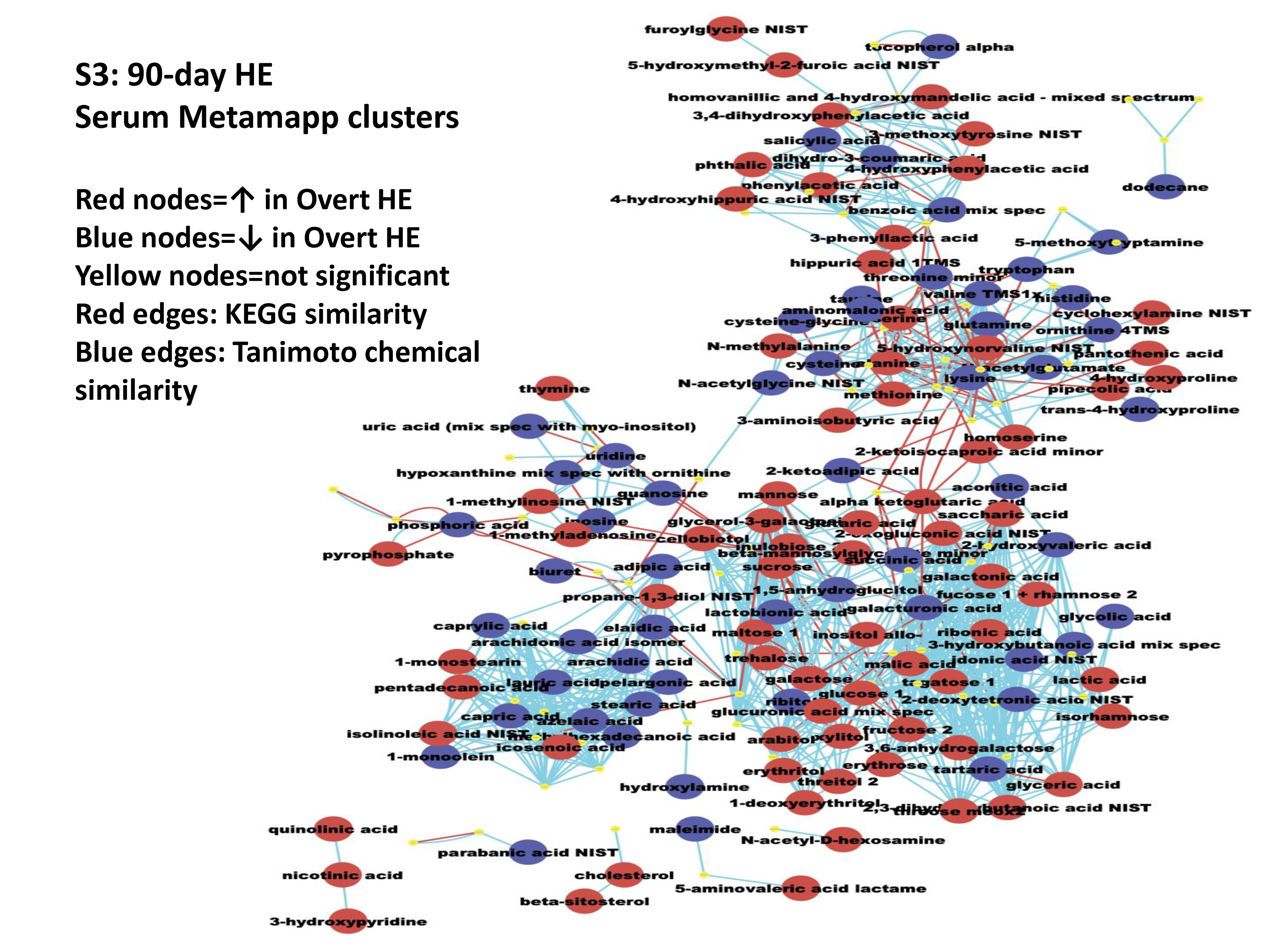

Supplement: S3 Fig — Red nodes = ↑ in those with outcomes, Blue nodes = ↓ in those with outcomes, Yellow nodes = not significant, Red edges: KEGG similarity and Blue edges: Tanimoto chemical. (TIF) [file pone.0223061.s004.tif]

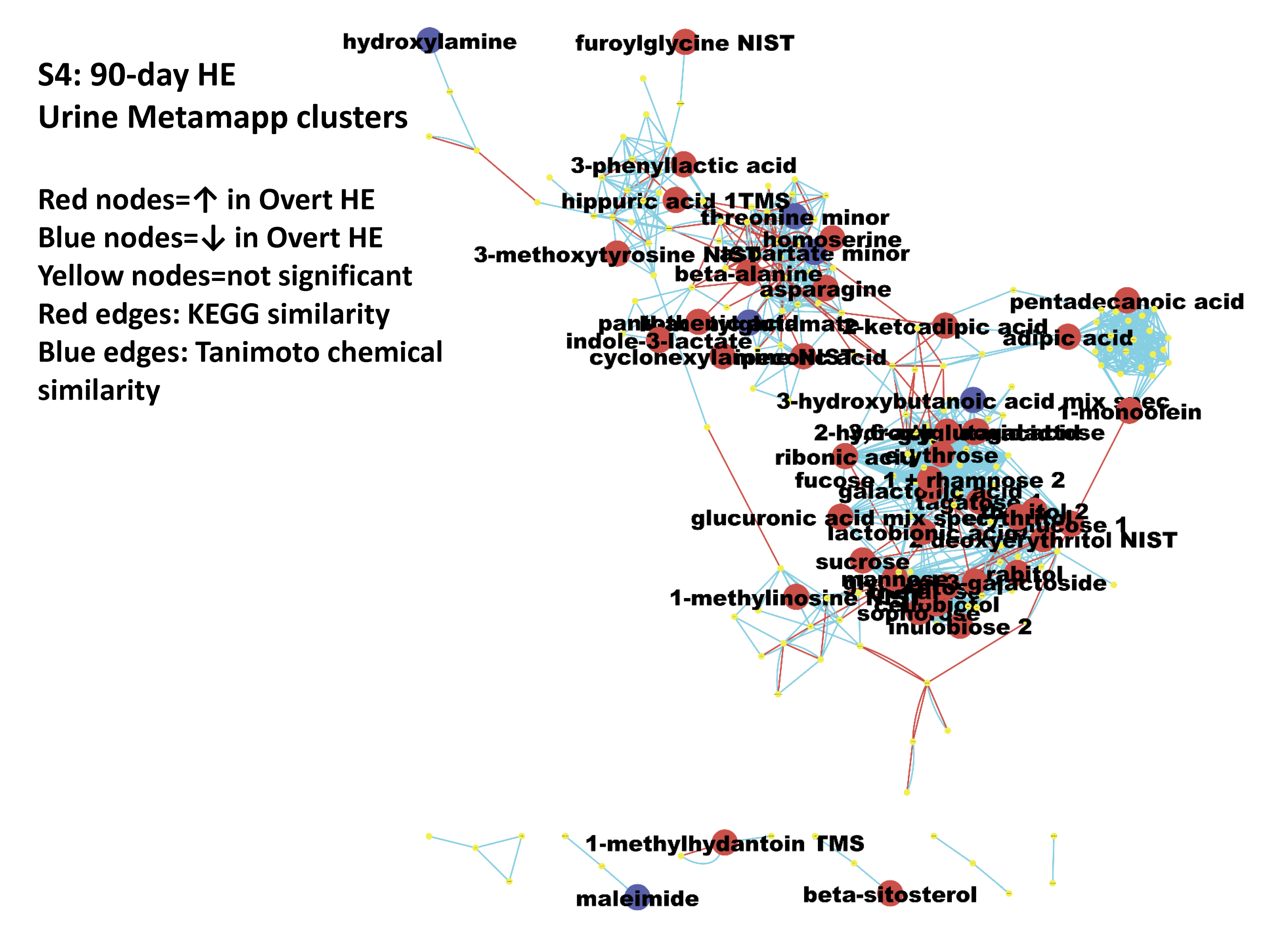

Supplement: S4 Fig — Red nodes = ↑ in those with outcomes, Blue nodes = ↓ in those with outcomes, Yellow nodes = not significant, Red edges: KEGG similarity and Blue edges: Tanimoto chemical. (TIF) [file pone.0223061.s005.tif]

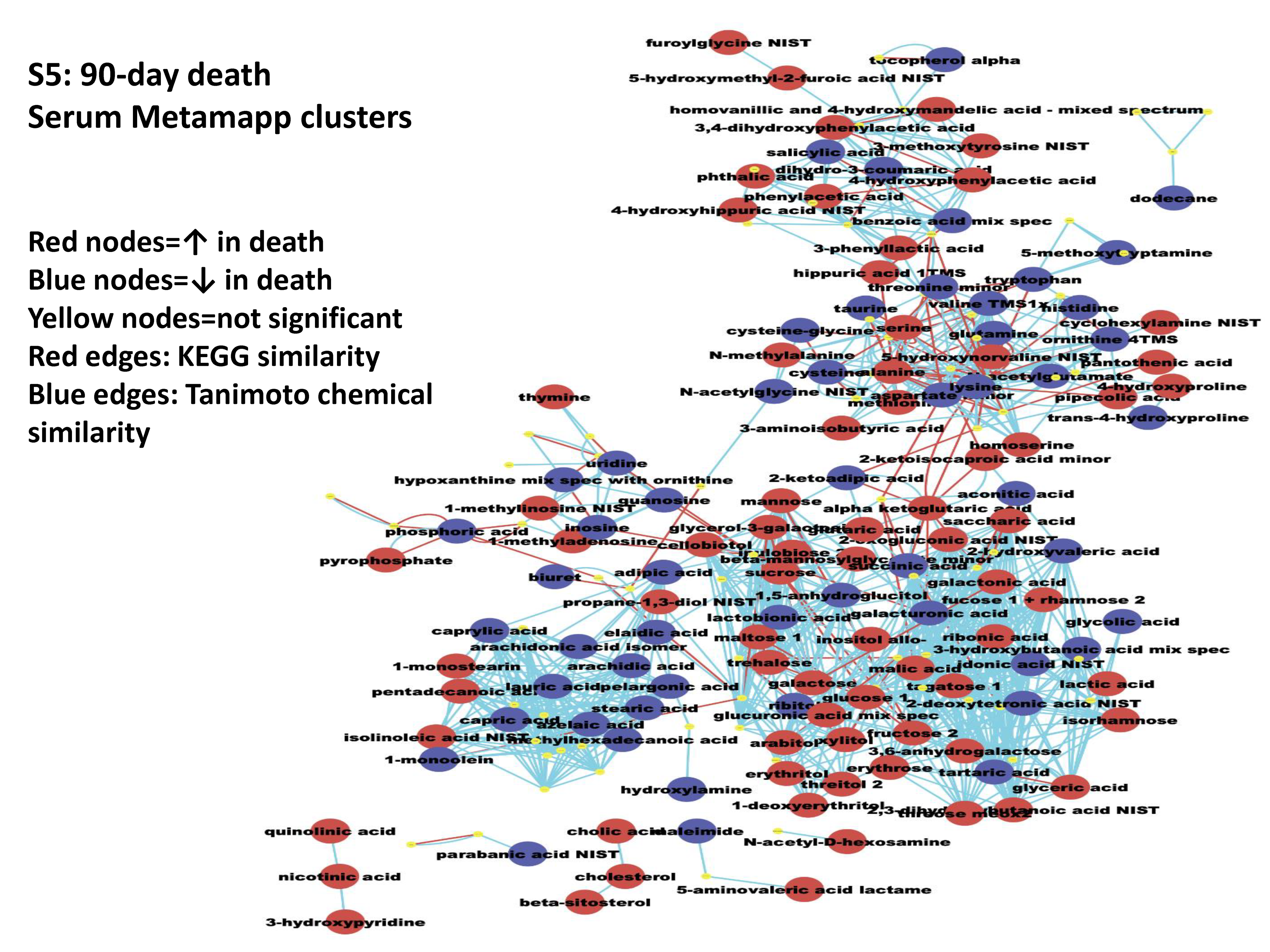

Supplement: S5 Fig — Red nodes = ↑ in those with outcomes, Blue nodes = ↓ in those with outcomes, Yellow nodes = not significant, Red edges: KEGG similarity and Blue edges: Tanimoto chemical. (TIF) [file pone.0223061.s006.tif]

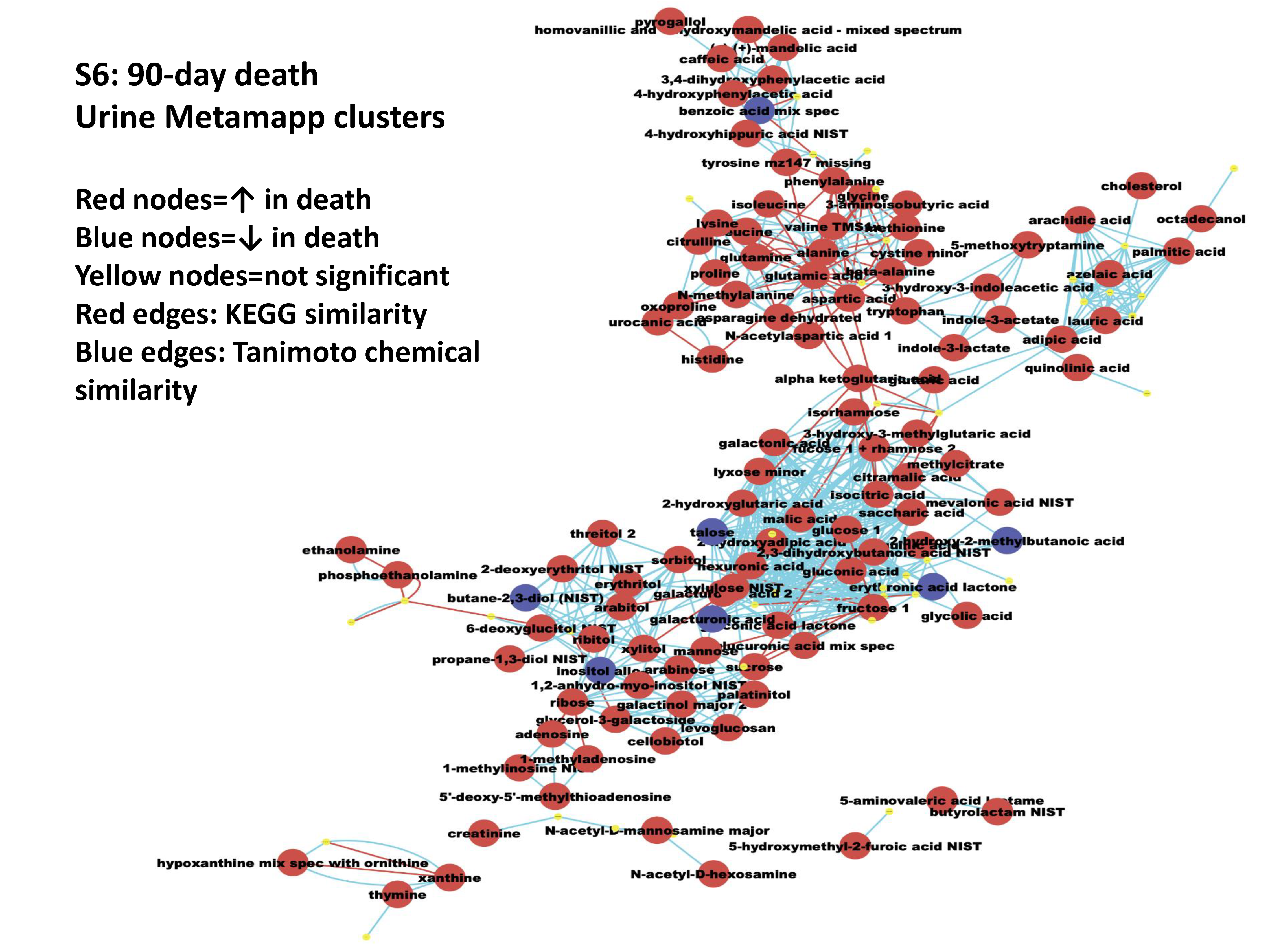

Supplement: S6 Fig — Red nodes = ↑ in those with outcomes, Blue nodes = ↓ in those with outcomes, Yellow nodes = not significant, Red edges: KEGG similarity and Blue edges: Tanimoto chemical. (TIF) [file pone.0223061.s007.tif]

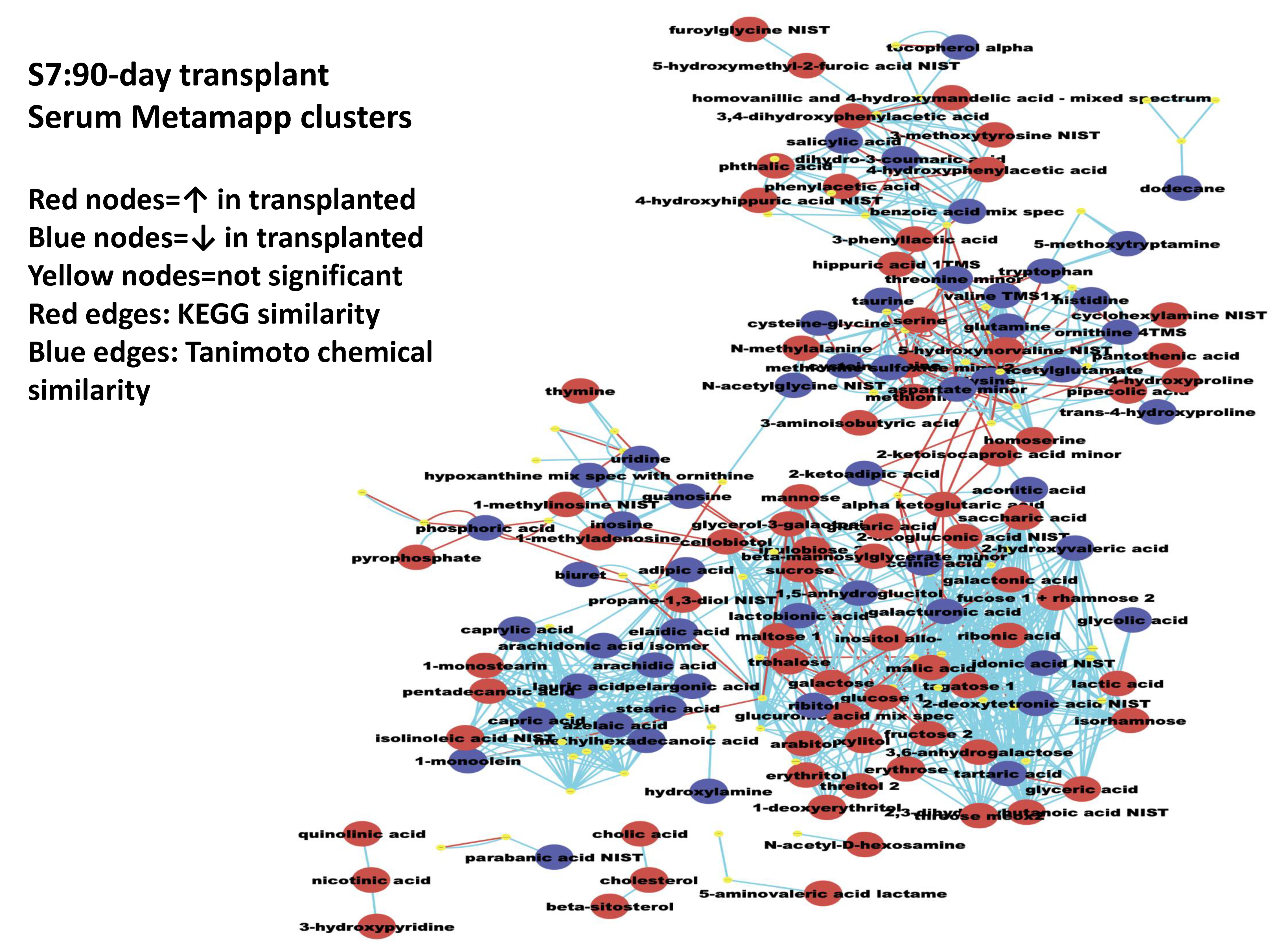

Supplement: S7 Fig — Red nodes = ↑ in those with outcomes, Blue nodes = ↓ in those with outcomes, Yellow nodes = not significant, Red edges: KEGG similarity and Blue edges: Tanimoto chemical. (TIF) [file pone.0223061.s008.tif]

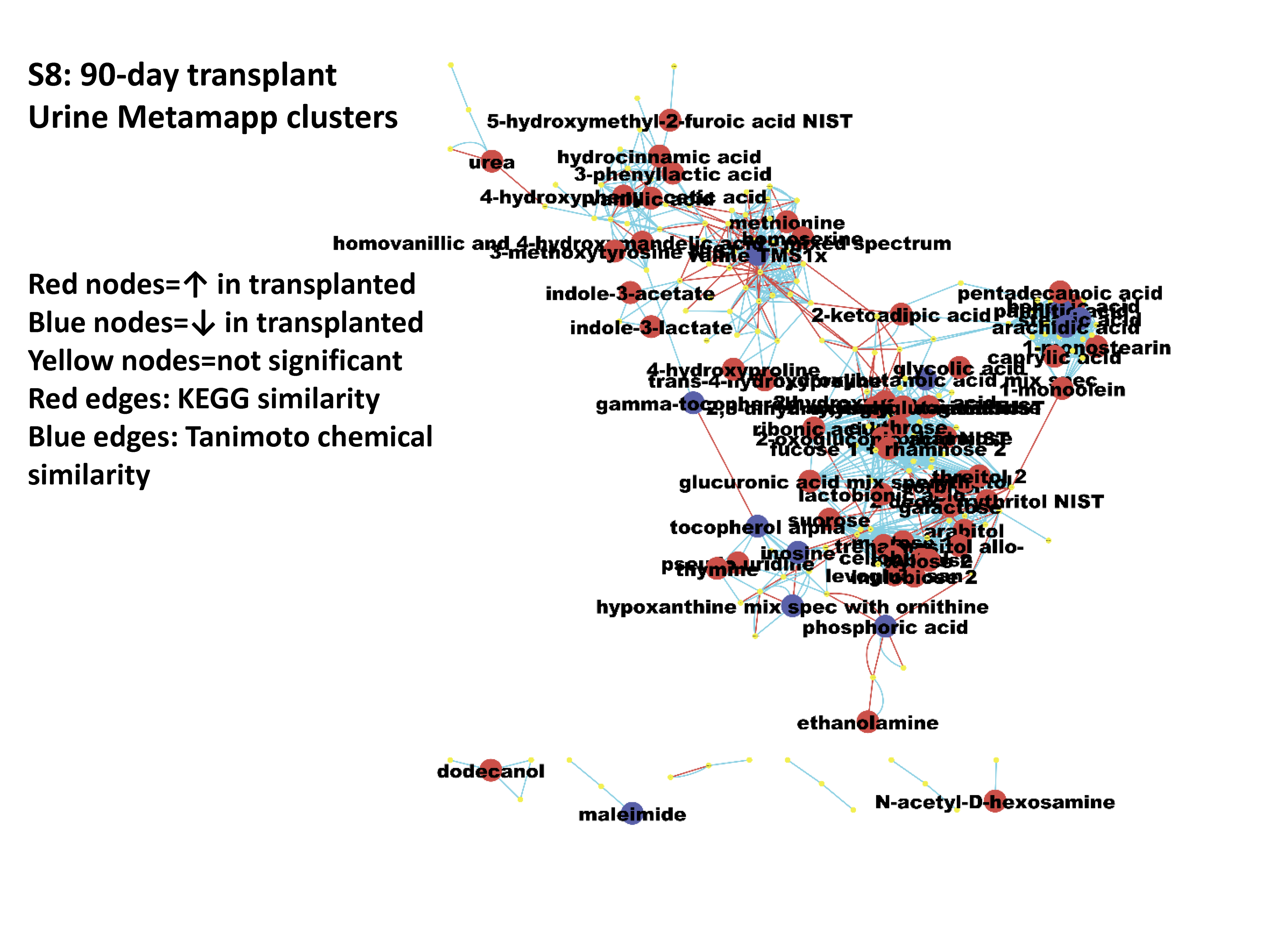

Supplement: S8 Fig — Red nodes = ↑ in those with outcomes, Blue nodes = ↓ in those with outcomes, Yellow nodes = not significant, Red edges: KEGG similarity and Blue edges: Tanimoto chemical. (TIF) [file pone.0223061.s009.tif]
