# Supplementary material for: Serum and urinary metabolomics and outcomes in cirrhosis
Source: PLoS One. 2019 Sep 27;14(9):e0223061. doi: 10.1371/journal.pone.0223061 (PMC6764675; doi:10.1371/journal.pone.0223061)
Supplement: S1 Table — (DOCX) [file pone.0223061.s010.docx]

| **Table S1: Overt HE prediction** | | | | | | | |
| --- | --- | --- | --- | --- | --- | --- | --- |
| **Serum Cluster name** | **Cluster size** | **p-value** | **FDR** | **Key compound** | **Altered metabolites** | **↑** | **↓** |
| Saturated FA | 12 | 1.4E-14 | 3.8E-13 | lauric acid | 9 | 1 | 8 |
| Pyridines | 3 | 2.7E-11 | 3.8E-10 | quinolinic acid | 3 | 3 | 0 |
| Phenylacetates | 8 | 3E-09 | 2.8E-08 | 4-hydroxyphenylacetic acid | 7 | 6 | 1 |
| Sugar Acids | 9 | 8.2E-09 | 5.7E-08 | ribonic acid | 7 | 6 | 1 |
| Deoxy Sugars | 3 | 2.7E-08 | 1.5E-07 | fucose 1 + rhamnose 2 | 3 | 2 | 1 |
| Hexoses | 7 | 3E-07 | 1.4E-06 | tagatose 1 | 6 | 6 | 0 |
| Sugar Alcohols | 13 | 4.8E-06 | 0.000019 | Erythritol | 9 | 8 | 1 |
| Amino Acids | 11 | 0.000038 | 0.00013 | threonine minor | 7 | 6 | 1 |
| Disaccharides | 7 | 0.000041 | 0.00013 | Sucrose | 6 | 5 | 1 |
| Amino Acids, Basic | 4 | 0.000056 | 0.00016 | Lysine | 3 | 0 | 3 |
| Purine Nucleosides | 4 | 0.00034 | 0.00086 | Guanosine | 4 | 2 | 2 |
| Dicarboxylic Acids | 6 | 0.00071 | 0.0016 | tartaric acid | 4 | 1 | 3 |
| Uronic Acids | 3 | 0.00072 | 0.0016 | glucuronic acid mix spec | 2 | 1 | 1 |
| Adipates | 3 | 0.0051 | 0.01 | adipic acid | 2 | 0 | 2 |
| Amino Acids, Sulfur | 4 | 0.025 | 0.045 | Methionine | 2 | 1 | 1 |
| UnSaturated FA | 8 | 0.026 | 0.045 | elaidic acid | 4 | 2 | 2 |
| Glutarates | 3 | 0.038 | 0.062 | glutaric acid | 2 | 2 | 0 |
| Monosaccharides | 3 | 0.041 | 0.063 | Erythrose | 2 | 2 | 0 |
| Pyrimidines | 3 | 0.05 | 0.073 | Thymine | 2 | 1 | 1 |
| Purinones | 3 | 0.057 | 0.08 | hypoxanthine mix spec with ornithine | 2 | 0 | 2 |
| **Urine Cluster name** | **Cluster size** | **p-value** | **FDR** | **Key compound** | **Altered metabolites** | **↑** | **↓** |
| Pentoses | 3 | 1.1E-16 | 2.4E-15 | lyxose minor | 3 | 3 | 0 |
| Indoles | 3 | 2.2E-16 | 2.4E-15 | indole-3-lactate | 3 | 3 | 0 |
| Amino Acids, Aromatic | 3 | 8.9E-16 | 6.5E-15 | Tryptophan | 3 | 3 | 0 |
| Purine Nucleosides | 4 | 1.5E-12 | 8.1E-12 | 5'-deoxy-5'-methylthioadenosine | 4 | 4 | 0 |
| Sugar Alcohols | 13 | 1.9E-10 | 8.3E-10 | Erythritol | 12 | 10 | 2 |
| Phenylacetates | 4 | 4.9E-10 | 1.8E-09 | 3,4-dihydroxyphenylacetic acid | 4 | 4 | 0 |
| Hexoses | 5 | 1E-08 | 3.2E-08 | levoglucosan | 5 | 4 | 1 |
| Amino Acids, Cyclic | 3 | 4.7E-08 | 1.3E-07 | Histidine | 3 | 3 | 0 |
| Amino Acids, Acidic | 3 | 1E-07 | 2.5E-07 | glutamic acid | 3 | 3 | 0 |
| Amino Acids, Basic | 3 | 2.3E-07 | 5E-07 | glutamine | 3 | 3 | 0 |
| Glutarates | 4 | 2.6E-07 | 5.1E-07 | 3-hydroxy-3-methylglutaric acid | 4 | 4 | 0 |
| Amino Acids | 10 | 4.9E-06 | 0.000009 | 3-aminoisobutyric acid | 8 | 8 | 0 |
| Malates | 3 | 0.000061 | 0.0001 | citramalic acid | 3 | 2 | 1 |
| Sugar Acids | 10 | 0.00044 | 0.00069 | saccharic acid | 6 | 6 | 0 |
| Hexuronic Acids | 3 | 0.00066 | 0.00096 | hexuronic acid | 2 | 2 | 0 |
| Dicarboxylic Acids | 5 | 0.0009 | 0.0012 | 2-hydroxyadipic acid | 2 | 2 | 0 |
| Saturated FA | 9 | 0.0011 | 0.0014 | azelaic acid | 4 | 4 | 0 |
| Disaccharides | 3 | 0.0025 | 0.0031 | sucrose | 3 | 3 | 0 |
| Citrates | 3 | 0.011 | 0.013 | isocitric acid | 2 | 2 | 0 |
| Purinones | 3 | 0.037 | 0.039 | hypoxanthine mix spec with ornithine | 2 | 2 | 0 |
| Amino Acids, Sulfur | 3 | 0.037 | 0.039 | methionine | 2 | 2 | 0 |
