# Supplementary material for: Serum and urinary metabolomics and outcomes in cirrhosis
Source: PLoS One. 2019 Sep 27;14(9):e0223061. doi: 10.1371/journal.pone.0223061 (PMC6764675; doi:10.1371/journal.pone.0223061)
Supplement: S2 Table — (DOCX) [file pone.0223061.s011.docx]

| **Table S2: Hospitalization prediction** | | | | | | | |
| --- | --- | --- | --- | --- | --- | --- | --- |
| **Serum Cluster name** | **Cluster size** | **p-values** | **FDR** | **Key compound** | **Altered metabolites** | **↑** | **↓** |
| Pyridines | 3 | 1.7E-09 | 4.7E-08 | quinolinic acid | 3 | 3 | 0 |
| Deoxy Sugars | 3 | 3.4E-09 | 4.7E-08 | isorhamnose | 3 | 2 | 1 |
| Saturated FA | 12 | 1.1E-08 | 1E-07 | pelargonic acid | 9 | 1 | 8 |
| Sugar Acids | 9 | 4.9E-08 | 3.5E-07 | 2-oxogluconic acid NIST | 8 | 6 | 2 |
| Sugar Alcohols | 13 | 8.1E-08 | 4.6E-07 | erythritol | 9 | 8 | 1 |
| Amino Acids, Basic | 4 | 2.1E-06 | 8.7E-06 | Lysine | 4 | 0 | 4 |
| Phenylacetates | 8 | 2.2E-06 | 8.7E-06 | 3-phenyllactic acid | 6 | 5 | 1 |
| Disaccharides | 7 | 0.000048 | 0.00016 | maltose 1 | 6 | 5 | 1 |
| Hexoses | 7 | 0.000053 | 0.00016 | tagatose 1 | 6 | 6 | 0 |
| Purine Nucleosides | 4 | 0.00034 | 0.00094 | inosine | 4 | 2 | 2 |
| Dicarboxylic Acids | 6 | 0.00039 | 0.00099 | tartaric acid | 3 | 0 | 3 |
| Amino Acids | 11 | 0.00043 | 0.001 | homoserine | 5 | 4 | 1 |
| Adipates | 3 | 0.0011 | 0.0025 | adipic acid | 2 | 0 | 2 |
| Uronic Acids | 3 | 0.0029 | 0.0057 | glucuronic acid mix spec | 2 | 1 | 1 |
| Amino Acids, Sulfur | 4 | 0.0043 | 0.0081 | cysteine | 3 | 1 | 2 |
| Glutarates | 3 | 0.038 | 0.066 | glutaric acid | 2 | 2 | 0 |
| Monosaccharides | 3 | 0.043 | 0.07 | erythrose | 2 | 2 | 0 |
| Pyrimidines | 3 | 0.048 | 0.071 | thymine | 2 | 1 | 1 |
| Purinones | 3 | 0.048 | 0.071 | hypoxanthine mix spec with ornithine | 2 | 0 | 2 |
| **Urine Cluster name** | **Cluster size** | **p-value** | **FDR** | **Key compound** | **Altered metabolites** | **↑** | **↓** |
| Pentoses | 3 | 3.3E-16 | 7.3E-15 | ribose | 3 | 3 | 0 |
| Purine Nucleosides | 4 | 3.8E-12 | 4.2E-11 | 5'-deoxy-5'-methyl  thioadenosine | 4 | 4 | 0 |
| Disaccharides | 3 | 6.4E-11 | 4.7E-10 | galactinol major 2 | 3 | 3 | 0 |
| Phenylacetates | 4 | 6.3E-10 | 3E-09 | 4-hydroxy  phenylacetic acid | 4 | 4 | 0 |
| Sugar Alcohols | 13 | 6.7E-10 | 3E-09 | cellobiotol | 12 | 10 | 2 |
| Amino Acids, Cyclic | 3 | 0.000001 | 3.7E-06 | histidine | 3 | 3 | 0 |
| Glutarates | 4 | 3.9E-06 | 0.000012 | 3-hydroxy-3-methylglutarate | 4 | 4 | 0 |
| Amino Acids, Basic | 3 | 5.3E-06 | 0.000014 | glutamine | 3 | 3 | 0 |
| Amino Acids | 10 | 0.00001 | 0.000025 | beta-alanine | 8 | 8 | 0 |
| Hexuronic Acids | 3 | 0.000013 | 0.000029 | hexuronic acid | 3 | 2 | 1 |
| Malates | 3 | 0.000041 | 0.000083 | erythronic acid lactone | 3 | 2 | 1 |
| Amino Acids, Acidic | 3 | 0.000049 | 0.000091 | glutamic acid | 3 | 3 | 0 |
| Hexoses | 5 | 0.000083 | 0.00014 | glucose 1 | 5 | 4 | 1 |
| Sugar Acids | 10 | 0.00046 | 0.00072 | saccharic acid | 6 | 6 | 0 |
| Citrates | 3 | 0.0025 | 0.0032 | isocitric acid | 2 | 2 | 0 |
| Indoles | 3 | 0.0025 | 0.0032 | indole-3-acetate | 3 | 3 | 0 |
| Amino Acids, Aromatic | 3 | 0.0025 | 0.0032 | tyrosine mz147 missing | 3 | 3 | 0 |
| Saturated FA | 9 | 0.0045 | 0.0055 | azelaic acid | 5 | 4 | 1 |
| Dicarboxylic Acids | 5 | 0.0054 | 0.0063 | 2-hydroxyadipic acid | 2 | 2 | 0 |
| Amino Acids, Sulfur | 3 | 0.017 | 0.018 | methionine | 2 | 2 | 0 |
| Purinones | 3 | 0.037 | 0.039 | hypoxanthine mix spec with ornithine | 2 | 2 | 0 |
