# Supplementary material for: Serum and urinary metabolomics and outcomes in cirrhosis
Source: PLoS One. 2019 Sep 27;14(9):e0223061. doi: 10.1371/journal.pone.0223061 (PMC6764675; doi:10.1371/journal.pone.0223061)
Supplement: S3 Table — (DOCX) [file pone.0223061.s012.docx]

| **Table S3: Death prediction** | | | | | | | |
| --- | --- | --- | --- | --- | --- | --- | --- |
| **Serum Cluster name** | **Cluster size** | **p-values** | **FDR** | **Key compound** | **Altered metabolites** | **↑** | **↓** |
| Purine Nucleosides | 4 | 1.1E-16 | 3.1E-15 | guanosine | 4 | 2 | 2 |
| Saturated FA | 12 | 8.5E-14 | 1.2E-12 | lauric acid | 9 | 1 | 8 |
| Pyridines | 3 | 8E-12 | 7.5E-11 | quinolinic acid | 3 | 3 | 0 |
| Phenylacetates | 8 | 8.6E-10 | 6E-09 | 4-hydroxyphenylacetic acid | 7 | 6 | 1 |
| Sugar Acids | 9 | 1.8E-08 | 1E-07 | ribonic acid | 7 | 6 | 1 |
| Deoxy Sugars | 3 | 2.7E-08 | 1.3E-07 | fucose 1 + rhamnose 2 | 3 | 2 | 1 |
| Hexoses | 7 | 3.7E-07 | 1.5E-06 | tagatose 1 | 6 | 6 | 0 |
| Sugar Alcohols | 13 | 6.6E-07 | 2.3E-06 | erythritol | 9 | 8 | 1 |
| Disaccharides | 7 | 1.6E-06 | 0.000005 | sucrose | 6 | 5 | 1 |
| Amino Acids | 11 | 0.000017 | 0.000047 | threonine minor | 7 | 6 | 1 |
| Amino Acids, Basic | 4 | 0.00019 | 0.00048 | lysine | 3 | 0 | 3 |
| Dicarboxylic Acids | 6 | 0.00041 | 0.00096 | tartaric acid | 3 | 1 | 2 |
| Uronic Acids | 3 | 0.0011 | 0.0024 | glucuronic acid mix spec | 2 | 1 | 1 |
| Adipates | 3 | 0.0027 | 0.0055 | adipic acid | 2 | 0 | 2 |
| Amino Acids, Acidic | 3 | 0.0045 | 0.0085 | N-acetylglutamate | 2 | 0 | 2 |
| Amino Acids, Sulfur | 4 | 0.022 | 0.039 | methionine | 2 | 1 | 1 |
| Unsaturated FA | 8 | 0.03 | 0.049 | elaidic acid | 3 | 1 | 2 |
| Glutarates | 3 | 0.038 | 0.058 | glutaric acid | 2 | 2 | 0 |
| Monosaccharides | 3 | 0.04 | 0.059 | erythrose | 2 | 2 | 0 |
| Pyrimidines | 3 | 0.056 | 0.079 | thymine | 2 | 1 | 1 |
| **Urine Cluster name** | **Cluster size** | **p-values** | **FDR** | **Key compound** | **Altered metabolites** | **↑** | **↓** |
| Pentoses | 3 | 2.2E-20 | 4.8E-19 | lyxose minor | 3 | 3 | 0 |
| Indoles | 3 | 1.1E-16 | 1.2E-15 | indole-3-lactate | 3 | 3 | 0 |
| Amino Acids, Aromatic | 3 | 7.8E-16 | 5.7E-15 | tryptophan | 3 | 3 | 0 |
| Purine Nucleosides | 4 | 2.1E-12 | 1.1E-11 | 5'-deoxy-5'-methylthioadenosine | 4 | 4 | 0 |
| Sugar Alcohols | 13 | 7.4E-11 | 3.2E-10 | erythritol | 12 | 10 | 2 |
| Phenylacetates | 4 | 2.4E-09 | 8.8E-09 | 3,4-dihydroxyphenylacetic acid | 4 | 4 | 0 |
| Hexoses | 5 | 3E-09 | 9.6E-09 | levoglucosan | 5 | 4 | 1 |
| Amino Acids, Cyclic | 3 | 3.3E-08 | 8.8E-08 | histidine | 3 | 3 | 0 |
| Amino Acids, Acidic | 3 | 3.6E-08 | 8.8E-08 | glutamic acid | 3 | 3 | 0 |
| Amino Acids, Basic | 3 | 1E-07 | 2.3E-07 | glutamine | 3 | 3 | 0 |
| Glutarates | 4 | 1.8E-07 | 3.6E-07 | 3-hydroxy-3-methylglutaric acid | 4 | 4 | 0 |
| Amino Acids | 10 | 7.9E-06 | 0.000015 | 3-aminoisobutyric acid | 8 | 8 | 0 |
| Malates | 3 | 0.000034 | 0.000057 | erythronic acid lactone | 3 | 2 | 1 |
| Hexuronic Acids | 3 | 0.00011 | 0.00017 | hexuronic acid | 3 | 2 | 1 |
| Sugar Acids | 10 | 0.00039 | 0.00058 | saccharic acid | 6 | 6 | 0 |
| Dicarboxylic Acids | 5 | 0.0015 | 0.002 | 2-hydroxyadipic acid | 2 | 2 | 0 |
| Disaccharides | 3 | 0.0025 | 0.0033 | sucrose | 3 | 3 | 0 |
| Saturated FA | 9 | 0.0029 | 0.0036 | azelaic acid | 4 | 4 | 0 |
| Citrates | 3 | 0.0045 | 0.0052 | isocitric acid | 2 | 2 | 0 |
| Purinones | 3 | 0.037 | 0.039 | hypoxanthine mix spec with ornithine | 2 | 2 | 0 |
| Amino Acids, Sulfur | 3 | 0.037 | 0.039 | methionine | 2 | 2 | 0 |
