# Supplementary material for: Serum and urinary metabolomics and outcomes in cirrhosis
Source: PLoS One. 2019 Sep 27;14(9):e0223061. doi: 10.1371/journal.pone.0223061 (PMC6764675; doi:10.1371/journal.pone.0223061)
Supplement: S4 Table — (DOCX) [file pone.0223061.s013.docx]

| **Table S4: Transplant prediction** | | | | | | | |
| --- | --- | --- | --- | --- | --- | --- | --- |
| **Serum Cluster name** | **Cluster size** | **p-values** | **FDR** | **Key compound** | **Altered metabolites** | **↑** | **↓** |
| Purine Nucleosides | 4 | 1.1E-16 | 3.1E-15 | guanosine | 4 | 2 | 2 |
| Pyridines | 3 | 8E-12 | 1.1E-10 | quinolinic acid | 3 | 3 | 0 |
| Phenylacetates | 8 | 6E-11 | 5.6E-10 | 4-hydroxyphenylacetic acid | 7 | 6 | 1 |
| Sugar Acids | 9 | 8.7E-10 | 6E-09 | ribonic acid | 7 | 6 | 1 |
| Saturated FA | 12 | 1.1E-09 | 6E-09 | stearic acid | 9 | 1 | 8 |
| Deoxy Sugars | 3 | 4.1E-09 | 1.9E-08 | fucose 1 + rhamnose 2 | 3 | 2 | 1 |
| Sugar Alcohols | 13 | 7.1E-07 | 2.9E-06 | erythritol | 9 | 8 | 1 |
| Hexoses | 7 | 1.6E-06 | 5.5E-06 | tagatose 1 | 6 | 6 | 0 |
| Amino Acids | 11 | 0.000019 | 0.00006 | threonine minor | 7 | 6 | 1 |
| Disaccharides | 7 | 0.00004 | 0.00011 | sucrose | 6 | 5 | 1 |
| Dicarboxylic Acids | 6 | 0.00023 | 0.00058 | tartaric acid | 3 | 1 | 2 |
| Uronic Acids | 3 | 0.0004 | 0.00093 | glucuronic acid mix spec | 2 | 1 | 1 |
| Amino Acids, Basic | 4 | 0.00068 | 0.0015 | lysine | 3 | 0 | 3 |
| Adipates | 3 | 0.0012 | 0.0023 | adipic acid | 2 | 0 | 2 |
| Amino Acids, Sulfur | 4 | 0.0079 | 0.015 | methionine | 3 | 1 | 2 |
| Amino Acids, Acidic | 3 | 0.0096 | 0.017 | N-acetylglutamate | 2 | 0 | 2 |
| Glutarates | 3 | 0.028 | 0.046 | glutaric acid | 2 | 2 | 0 |
| **Urine Cluster name** | **Cluster size** | **p-values** | **FDR** | **Key compound** | **Altered metabolites** | **↑** | **↓** |
| Pentoses | 3 | 2.2E-20 | 4.8E-19 | lyxose minor | 3 | 3 | 0 |
| Indoles | 3 | 1.1E-16 | 1.2E-15 | indole-3-lactate | 3 | 3 | 0 |
| Amino Acids, Aromatic | 3 | 1E-12 | 7.3E-12 | phenylalanine | 3 | 3 | 0 |
| Purine Nucleosides | 4 | 5.1E-12 | 2.8E-11 | 5'-deoxy-5'-methylthioadenosine | 4 | 4 | 0 |
| Sugar Alcohols | 13 | 6.7E-11 | 3E-10 | erythritol | 12 | 10 | 2 |
| Hexoses | 5 | 1.8E-09 | 6.7E-09 | glucose 1 | 5 | 4 | 1 |
| Phenylacetates | 4 | 6.9E-09 | 2.2E-08 | 3,4-dihydroxyphenylacetic acid | 4 | 4 | 0 |
| Amino Acids, Cyclic | 3 | 8E-08 | 2.2E-07 | histidine | 3 | 3 | 0 |
| Amino Acids, Basic | 3 | 2.7E-07 | 6.7E-07 | glutamine | 3 | 3 | 0 |
| Glutarates | 4 | 4.2E-07 | 9.1E-07 | 3-hydroxy-3-methylglutaric acid | 4 | 4 | 0 |
| Amino Acids, Acidic | 3 | 4.6E-07 | 9.1E-07 | glutamic acid | 3 | 3 | 0 |
| Amino Acids | 10 | 9.1E-06 | 0.000017 | 3-aminoisobutyric acid | 8 | 8 | 0 |
| Malates | 3 | 0.00003 | 0.00005 | erythronic acid lactone | 3 | 2 | 1 |
| Hexuronic Acids | 3 | 0.00021 | 0.00033 | hexuronic acid | 2 | 2 | 0 |
| Sugar Acids | 10 | 0.00041 | 0.00061 | saccharic acid | 6 | 6 | 0 |
| Dicarboxylic Acids | 5 | 0.0022 | 0.003 | 2-hydroxyadipic acid | 2 | 2 | 0 |
| Disaccharides | 3 | 0.0025 | 0.0033 | sucrose | 3 | 3 | 0 |
| Saturated FA | 9 | 0.0035 | 0.0043 | azelaic acid | 5 | 4 | 1 |
| Citrates | 3 | 0.005 | 0.0058 | isocitric acid | 2 | 2 | 0 |
| Purinones | 3 | 0.037 | 0.04 | hypoxanthine mix spec with ornithine | 2 | 2 | 0 |
| Amino Acids, Sulfur | 3 | 0.038 | 0.04 | methionine | 2 | 2 | 0 |
