# Supplementary material for: Serum and urinary metabolomics and outcomes in cirrhosis
Source: PLoS One. 2019 Sep 27;14(9):e0223061. doi: 10.1371/journal.pone.0223061 (PMC6764675; doi:10.1371/journal.pone.0223061)
Supplement: S5 Table — (DOCX) [file pone.0223061.s014.docx]

| Table S5: Logistic regression of serum metabolites with 90-day hospitalizations | | | | |
| --- | --- | --- | --- | --- |
| index | label | regression_coefficient | p_value | p_values_adjusted |
| 5 | stearic acid | -3.1472 | 2.15E-06 | 0 |
| 17 | threonine minor | -2.6485 | 2.36E-06 | 0 |
| 51 | erythritol | 3.7564 | 2.18E-06 | 0 |
| 72 | methylhexadecanoic acid | -2.0121 | 1.33E-06 | 0 |
| 75 | inosine | -3.0141 | 1.82E-06 | 0 |
| 109 | 2-hydroxyvaleric acid | -2.3273 | 7.03E-07 | 0 |
| 113 | histidine | -2.3242 | 2.6E-06 | 0 |
| 125 | isorhamnose | 2.1895 | 1.62E-06 | 0 |
| 130 | arachidic acid | -2.8826 | 1.89E-06 | 0 |
| 149 | adipic acid | -2.9244 | 2.42E-06 | 0 |
| 173 | N-acetylglycine NIST | -1.7252 | 7.55E-07 | 0 |
| 187 | 2-ketoisocaproic acid minor | 1.9915 | 6.18E-07 | 0 |
| 190 | tartaric acid | -2.3384 | 4.48E-07 | 0 |
| 194 | phosphoric acid.1 | -3.6606 | 2.47E-06 | 0 |
| 198 | 5-hydroxyindole-3-acetic acid NIST | 2.7774 | 2.25E-06 | 0 |
| 208 | quinolinic acid | 2.2114 | 1.03E-06 | 0 |
| 228 | 5-aminovaleric acid lactame | 4.7953 | 7.62E-07 | 0 |
| 230 | pantothenic acid | 3.3851 | 6.26E-07 | 0 |
| 301 | X607692 | -2.1672 | 2.81E-06 | 0 |
| 318 | X640860 | -1.1022 | 1.99E-06 | 0 |
| 359 | X211952 | -2.0129 | 1.33E-06 | 0 |
| 40 | glutamine 2TMS | -3.279 | 5.65E-06 | 0.0001 |
| 43 | isoleucine minor | -4.5228 | 4.26E-06 | 0.0001 |
| 47 | capric acid | -2.8078 | 0 | 0.0001 |
| 73 | fructose 1 | 1.6784 | 7.12E-06 | 0.0001 |
| 85 | xylitol | 3.8494 | 4.13E-06 | 0.0001 |
| 87 | threitol 2 | 3.819 | 3.87E-06 | 0.0001 |
| 92 | phosphoric acid | -7.217 | 6.59E-06 | 0.0001 |
| 98 | phenylethylamine | 3.3691 | 4.58E-06 | 0.0001 |
| 104 | maltose 1 | 4.2989 | 0 | 0.0001 |
| 119 | cysteine | -2.3611 | 0 | 0.0001 |
| 134 | 4-hydroxyphenylacetic acid | 1.9188 | 8.84E-06 | 0.0001 |
| 146 | nicotinic acid | 9.0147 | 6.12E-06 | 0.0001 |
| 148 | 3,6-anhydrogalactose | 4.1495 | 0 | 0.0001 |
| 150 | 5-methoxytryptamine | -1.2242 | 7.02E-06 | 0.0001 |
| 159 | parabanic acid NIST | -1.6704 | 3.33E-06 | 0.0001 |
| 167 | galactose | 6.4201 | 0 | 0.0001 |
| 179 | 2-oxogluconic acid NIST | 4.1291 | 0 | 0.0001 |
| 181 | 1-deoxyerythritol | 2.66 | 7.59E-06 | 0.0001 |
| 185 | galactonic acid | 2.2557 | 4.93E-06 | 0.0001 |
| 186 | ribonic acid | 2.972 | 3.66E-06 | 0.0001 |
| 193 | phthalic acid | 2.2922 | 0 | 0.0001 |
| 196 | asparagine 2TMS minor | 5.7435 | 0 | 0.0001 |
| 223 | N-acetyl-D-tryptophan minor2 | 3.9734 | 0 | 0.0001 |
| 231 | inulobiose 2 | 3.362 | 0 | 0.0001 |
| 234 | homoserine | 3.088 | 0 | 0.0001 |
| 238 | mannose | 5.5383 | 0 | 0.0001 |
| 241 | 3,4-dihydroxyphenylacetic acid | 1.7678 | 0 | 0.0001 |
| 255 | X455340 | -2.1121 | 0 | 0.0001 |
| 262 | X289052 | -1.2917 | 0 | 0.0001 |
| 275 | X223597 | 1.7534 | 0 | 0.0001 |
| 284 | X223548 | -1.4446 | 4.25E-06 | 0.0001 |
| 300 | X597213 | -3.9885 | 0 | 0.0001 |
| 326 | X618071 | -4.1552 | 4.15E-06 | 0.0001 |
| 332 | X484792 | -1.6296 | 0 | 0.0001 |
| 335 | X307915 | -2.1677 | 4.31E-06 | 0.0001 |
| 360 | X208557 | -2.1271 | 9.79E-06 | 0.0001 |
| 363 | X356925 | 3.5937 | 0 | 0.0001 |
| 6 | pelargonic acid | -4.7296 | 0 | 0.0002 |
| 26 | serine minor | -1.4643 | 0 | 0.0002 |
| 28 | lauric acid | -1.6297 | 0 | 0.0002 |
| 68 | glutamine dehydrated 2TMS minor | -2.016 | 0 | 0.0002 |
| 71 | oxalic acid | -1.5737 | 0 | 0.0002 |
| 84 | arabitol | 1.789 | 0.0001 | 0.0002 |
| 95 | valine TMS1x | -1.9354 | 0 | 0.0002 |
| 107 | pipecolic acid | 5.47 | 0 | 0.0002 |
| 110 | glucuronic acid mix spec | 5.5313 | 0 | 0.0002 |
| 120 | tagatose 1 | 1.9556 | 0 | 0.0002 |
| 139 | hypoxanthine mix spec with ornithine | -1.7012 | 0 | 0.0002 |
| 144 | 4-hydroxyproline | 1.5525 | 0 | 0.0002 |
| 145 | 3-phenyllactic acid | 2.725 | 0 | 0.0002 |
| 213 | cyclohexylamine NIST | 3.487 | 0.0001 | 0.0002 |
| 216 | erythrose | 3.4305 | 0 | 0.0002 |
| 251 | X213253 | -6.007 | 0 | 0.0002 |
| 285 | X201862 | -2.0663 | 0 | 0.0002 |
| 287 | X223566 | -6.827 | 0.0001 | 0.0002 |
| 290 | X228911 | 3.554 | 0 | 0.0002 |
| 291 | X381876 | 2.2665 | 0 | 0.0002 |
| 317 | X216428 | 4.0205 | 0 | 0.0002 |
| 320 | X213193 | 7.1634 | 0 | 0.0002 |
| 329 | X223629 | 3.0751 | 0 | 0.0002 |
| 337 | X486017 | -1.6183 | 0 | 0.0002 |
| 344 | X227367 | 4.0954 | 0 | 0.0002 |
| 345 | X653345 | 2.1569 | 0 | 0.0002 |
| 347 | X362005 | -5.1118 | 0 | 0.0002 |
| 350 | X617556 | 1.5005 | 0.0001 | 0.0002 |
| 355 | X224849 | 4.7244 | 0 | 0.0002 |
| 357 | X231850 | 1.4725 | 0.0001 | 0.0002 |
| 362 | X216838 | -4.6251 | 0 | 0.0002 |
| 48 | caprylic acid | -1.6843 | 0.0001 | 0.0003 |
| 115 | fucose | 1.8153 | 0.0001 | 0.0003 |
| 128 | asparagine dehydrated | -1.1469 | 0.0001 | 0.0003 |
| 152 | pyrophosphate | 6.7632 | 0.0001 | 0.0003 |
| 168 | propane-1,3-diol NIST | 1.3932 | 0.0001 | 0.0003 |
| 236 | 1-methyladenosine | 1.4354 | 0.0001 | 0.0003 |
| 250 | X222169 | 1.167 | 0.0001 | 0.0003 |
| 259 | X211979 | -1.3545 | 0.0001 | 0.0003 |
| 315 | X455826 | -1.3449 | 0.0001 | 0.0003 |
| 316 | X314770 | 5.7411 | 0.0001 | 0.0003 |
| 328 | X497413 | 2.1057 | 0.0001 | 0.0003 |
| 336 | X495239 | -3.4626 | 0.0001 | 0.0003 |
| 45 | lysine | -1.6081 | 0.0001 | 0.0004 |
| 74 | methionine | 1.681 | 0.0001 | 0.0004 |
| 156 | glutaric acid | 1.4771 | 0.0001 | 0.0004 |
| 166 | guanosine | -1.1222 | 0.0001 | 0.0004 |
| 174 | beta-sitosterol | 1.9433 | 0.0001 | 0.0004 |
| 206 | beta-mannosylglycerate minor | 6.0266 | 0.0001 | 0.0004 |
| 271 | X612625 | 1.0621 | 0.0001 | 0.0004 |
| 296 | X438101 | 1.7532 | 0.0001 | 0.0004 |
| 298 | X223505 | 7.7751 | 0.0001 | 0.0004 |
| 310 | X508725 | 3.1843 | 0.0001 | 0.0004 |
| 331 | X367932 | -1.0551 | 0.0001 | 0.0004 |
| 93 | sucrose | 2.707 | 0.0002 | 0.0005 |
| 133 | threonic acid 1 | -9.9581 | 0.0002 | 0.0005 |
| 151 | 1-monoolein | -0.9494 | 0.0001 | 0.0005 |
| 224 | lactobionic acid | -1.1254 | 0.0002 | 0.0007 |
| 240 | 2-ketoadipic acid | -1.636 | 0.0002 | 0.0007 |
| 274 | X213143 | 15.0043 | 0.0002 | 0.0007 |
| 286 | X234717 | 1.6589 | 0.0002 | 0.0007 |
| 309 | X199596 | -2.2842 | 0.0002 | 0.0007 |
| 36 | ornithine 4TMS | -1.5334 | 0.0003 | 0.0008 |
| 91 | fucose 1 + rhamnose 2 | 1.6134 | 0.0003 | 0.0008 |
| 297 | X213972 | -10.1888 | 0.0003 | 0.0008 |
| 323 | X223871 | 1.5783 | 0.0003 | 0.0008 |
| 60 | methanolphosphate | 1.2274 | 0.0003 | 0.0009 |
| 101 | alanine 3TMS | -1.3236 | 0.0003 | 0.0009 |
| 245 | 1-methylinosine NIST | 1.0084 | 0.0003 | 0.0009 |
| 254 | X356938 | -10.6774 | 0.0003 | 0.0009 |
| 267 | X200850 | -1.5133 | 0.0003 | 0.0009 |
| 172 | pyruvic acid | -1.757 | 0.0004 | 0.001 |
| 288 | X438057 | 1.817 | 0.0004 | 0.001 |
| 171 | dodecane | -1.2809 | 0.0004 | 0.0011 |
| 121 | elaidic acid | -1.0644 | 0.0004 | 0.0012 |
| 203 | cellobiotol | 1.8487 | 0.0005 | 0.0014 |
| 12 | lactic acid | 0.8837 | 0.0005 | 0.0015 |
| 34 | trans-4-hydroxyproline | -1.0381 | 0.0006 | 0.0017 |
| 322 | X227352 | 20.3353 | 0.0006 | 0.0017 |
| 24 | proline | 1.5494 | 0.0007 | 0.0018 |
| 161 | 3-aminoisobutyric acid | 1.3804 | 0.0007 | 0.0018 |
| 58 | glutamine dehydrated | -1.1896 | 0.0007 | 0.0019 |
| 188 | hippuric acid 1TMS | 1.0136 | 0.0007 | 0.0019 |
| 199 | methionine sulfoxide minor1 | 1.4885 | 0.0007 | 0.0019 |
| 127 | idonic acid NIST | -1.3897 | 0.0009 | 0.0022 |
| 131 | methionine sulfoxide major | -1.059 | 0.0009 | 0.0022 |
| 293 | X537868 | -2.7475 | 0.0009 | 0.0022 |
| 324 | X309540 | -1.0645 | 0.0009 | 0.0022 |
| 22 | ribitol | -0.8628 | 0.0009 | 0.0023 |
| 325 | X223625 | 0.991 | 0.0011 | 0.0027 |
| 10 | glucose 2 | -1.1808 | 0.0012 | 0.003 |
| 183 | thymine | 1.0038 | 0.0012 | 0.003 |
| 189 | 3-hydroxypyridine | 1.2877 | 0.0012 | 0.003 |
| 313 | X273773 | 10.2308 | 0.0012 | 0.003 |
| 122 | biuret | -1.1344 | 0.0013 | 0.0031 |
| 164 | azelaic acid | -0.6967 | 0.0013 | 0.0031 |
| 367 | X270508 | 1.2912 | 0.0014 | 0.0034 |
| 50 | 1,5-anhydroglucitol | -1.3909 | 0.0015 | 0.0036 |
| 147 | glycerol-3-galactoside | 1.0264 | 0.0016 | 0.0037 |
| 278 | X339455 | -1.0137 | 0.0016 | 0.0038 |
| 165 | alpha ketoglutaric acid | 1.4933 | 0.0017 | 0.0039 |
| 175 | salicylic acid | -0.6964 | 0.0017 | 0.0039 |
| 279 | X321685 | -1.09 | 0.0017 | 0.0039 |
| 30 | cholesterol | 1.1534 | 0.0018 | 0.0041 |
| 346 | X612627 | 3.3419 | 0.0018 | 0.0041 |
| 96 | 2-deoxytetronic acid NIST | -0.9588 | 0.0019 | 0.0042 |
| 233 | N-acetyl-D-mannosamine major | 0.9143 | 0.002 | 0.0044 |
| 79 | glyceric acid | 1.081 | 0.002 | 0.0045 |
| 78 | glycolic acid | -1.111 | 0.0023 | 0.005 |
| 265 | X207223 | -20.6246 | 0.0025 | 0.0054 |
| 20 | phenylalanine TMS1x | -1.006 | 0.0025 | 0.0055 |
| 81 | N-acetylglutamate | -1.088 | 0.0027 | 0.0057 |
| 212 | cysteine-glycine | -0.8093 | 0.0026 | 0.0057 |
| 154 | 1-monostearin | 0.9933 | 0.0029 | 0.0062 |
| 15 | fructose 2 | 0.7598 | 0.003 | 0.0063 |
| 117 | mannitol mix spec with histidine | -5.4134 | 0.003 | 0.0063 |
| 289 | X223618 | -0.8541 | 0.0033 | 0.0069 |
| 38 | succinic acid | -0.9332 | 0.0034 | 0.0071 |
| 170 | pentadecanoic acid | 0.9482 | 0.0034 | 0.0071 |
| 11 | alanine | 0.801 | 0.0035 | 0.0072 |
| 100 | N-methylalanine | 0.872 | 0.0037 | 0.0075 |
| 361 | X428311 | -0.9665 | 0.0038 | 0.0077 |
| 158 | 2-deoxyerythritol | 1.0605 | 0.0039 | 0.008 |
| 176 | isolinoleic acid NIST | 0.7768 | 0.0039 | 0.008 |
| 137 | phenylacetic acid | 0.987 | 0.0042 | 0.0085 |
| 23 | glutamine | -1.0086 | 0.0045 | 0.0088 |
| 180 | dihydro-3-coumaric acid | -0.7938 | 0.0044 | 0.0088 |
| 54 | glutamate TMS2x | -0.7795 | 0.0048 | 0.0094 |
| 277 | X199794 | -1.5111 | 0.005 | 0.0098 |
| 239 | homovanillic and 4-hydroxymandelic acid - mixed spectrum | 0.9414 | 0.0051 | 0.0099 |
| 33 | tocopherol alpha | -0.9174 | 0.0052 | 0.0101 |
| 88 | shikimic acid | -0.8097 | 0.0053 | 0.0101 |
| 90 | taurine | -0.904 | 0.0053 | 0.0102 |
| 314 | X465393 | -0.9124 | 0.0056 | 0.0107 |
| 63 | methionine sulfoxide minor2 | -0.8286 | 0.0066 | 0.0125 |
| 351 | X486054 | 1.1724 | 0.0066 | 0.0125 |
| 338 | X485397 | -22.3013 | 0.0069 | 0.013 |
| 220 | 2,3-dihydroxybutanoic acid NIST | 0.7963 | 0.0078 | 0.0146 |
| 257 | X268506 | -0.8079 | 0.0083 | 0.0155 |
| 3 | tryptophan | -0.8361 | 0.009 | 0.0167 |
| 205 | shikimic acid.1 | -0.7515 | 0.0092 | 0.017 |
| 295 | X199942 | 0.9371 | 0.0093 | 0.0172 |
| 153 | ornithine | -0.6778 | 0.0095 | 0.0173 |
| 319 | X277432 | -0.8833 | 0.01 | 0.0182 |
| 13 | glycine | -0.7624 | 0.0103 | 0.0186 |
| 201 | N-acetyl-D-hexosamine | 0.7171 | 0.0104 | 0.0187 |
| 349 | X226912 | -0.7272 | 0.0112 | 0.02 |
| 356 | X566268 | 0.8965 | 0.0117 | 0.0209 |
| 178 | aconitic acid | -0.735 | 0.012 | 0.0212 |
| 126 | galacturonic acid | -0.7263 | 0.0124 | 0.0219 |
| 82 | asparagine minor 2 | -1.0922 | 0.0127 | 0.0222 |
| 252 | X309642 | 0.6809 | 0.0139 | 0.0243 |
| 311 | X565868 | 0.7459 | 0.0144 | 0.025 |
| 226 | trehalose | 0.8284 | 0.0146 | 0.0252 |
| 163 | threose meox2 | 0.624 | 0.0151 | 0.0259 |
| 312 | X301325 | 47.481 | 0.0153 | 0.0263 |
| 340 | X537746 | 0.7007 | 0.0159 | 0.0271 |
| 16 | hydroxylamine | -0.7554 | 0.0163 | 0.0276 |
| 219 | beta-alanine minor | 0.7838 | 0.0163 | 0.0276 |
| 55 | glucose 1 | 0.6391 | 0.0184 | 0.0309 |
| 215 | saccharic acid | 1.0357 | 0.0189 | 0.0317 |
| 141 | inositol allo- | 1.0952 | 0.0196 | 0.0327 |
| 263 | X220010 | 0.6691 | 0.0202 | 0.0336 |
| 204 | 5-hydroxymethyl-2-furoic acid NIST | 0.7009 | 0.0207 | 0.0342 |
| 333 | X484689 | -0.6752 | 0.0214 | 0.0352 |
| 266 | X223521 | 0.6796 | 0.0222 | 0.0363 |
| 247 | X199777 | -0.7684 | 0.0226 | 0.0368 |
| 365 | X225430 | 0.5743 | 0.0233 | 0.0379 |
| 276 | X566628 | -0.7432 | 0.0243 | 0.0393 |
| 112 | uridine | -0.6323 | 0.0261 | 0.0421 |
| 2 | uric acid (mix spec with myo-inositol) | -0.7517 | 0.027 | 0.0432 |
| 207 | 3-aminoisobutyric acid 1 | 0.8161 | 0.0273 | 0.0436 |
| 52 | arachidonic acid isomer | -0.5852 | 0.0282 | 0.0448 |
| 341 | X486090 | -0.6023 | 0.0284 | 0.0449 |
