# Supplementary material for: Serum and urinary metabolomics and outcomes in cirrhosis
Source: PLoS One. 2019 Sep 27;14(9):e0223061. doi: 10.1371/journal.pone.0223061 (PMC6764675; doi:10.1371/journal.pone.0223061)
Supplement: S6 Table — (DOCX) [file pone.0223061.s015.docx]

| Table S6: Logistic regression of urine metabolites with 90-day hospitalizations | | | | |
| --- | --- | --- | --- | --- |
| index | label | regression_coefficient | p_value | p_values_adjusted |
| 9 | isothreonic acid | 2.0011 | 2.77E-06 | 0.0001 |
| 18 | indole-3-acetate | 1.9795 | 0 | 0.0001 |
| 23 | tyrosine mz147 missing | 1.7764 | 0 | 0.0001 |
| 29 | arabinose | 2.2123 | 6.9E-06 | 0.0001 |
| 43 | indole-3-lactate | 2.2569 | 8.27E-06 | 0.0001 |
| 47 | ethanolamine | 1.5159 | 0 | 0.0001 |
| 54 | 4-hydroxyphenylacetic acid | 3.7474 | 0 | 0.0001 |
| 62 | isocitric acid | 1.4469 | 0 | 0.0001 |
| 68 | cellobiotol | 1.6303 | 0 | 0.0001 |
| 69 | urocanic acid | 1.6005 | 0 | 0.0001 |
| 70 | ribose | 1.7061 | 0 | 0.0001 |
| 79 | saccharic acid | 1.7317 | 5.18E-06 | 0.0001 |
| 82 | fucose | 1.8679 | 9.8E-06 | 0.0001 |
| 89 | hypoxanthine mix spec with ornithine | 1.5415 | 0 | 0.0001 |
| 92 | N-acetyl-D-mannosamine 3 | 2.2394 | 4.36E-06 | 0.0001 |
| 93 | 5-aminovaleric acid lactame | 2.6325 | 0 | 0.0001 |
| 94 | 3-hydroxy-3-indoleacetic acid | 1.7928 | 0 | 0.0001 |
| 96 | xylulose NIST | 1.7333 | 0 | 0.0001 |
| 98 | 2-hydroxyadipic acid | 2.0662 | 0 | 0.0001 |
| 99 | 1,2-anhydro-myo-inositol NIST | 3.5688 | 0 | 0.0001 |
| 103 | azelaic acid | 2.168 | 7.48E-06 | 0.0001 |
| 114 | N-acetyl-D-hexosamine | 2.5961 | 7.56E-06 | 0.0001 |
| 115 | 5'-deoxy-5'-methylthioadenosine | 1.7667 | 0 | 0.0001 |
| 118 | 2,3-dihydroxybutanoic acid NIST | 2.6905 | 6.66E-06 | 0.0001 |
| 119 | serine minor | 1.5898 | 0 | 0.0001 |
| 120 | glucuronic acid mix spec | 2.0314 | 1.57E-06 | 0.0001 |
| 122 | lyxose minor | 2.2757 | 6.58E-06 | 0.0001 |
| 130 | homovanillic and 4-hydroxymandelic acid - mixed spectrum | 2.3926 | 4.26E-06 | 0.0001 |
| 131 | glutamic acid | 2.4478 | 0 | 0.0001 |
| 132 | galactinol major 2 | 2.3214 | 0 | 0.0001 |
| 134 | isorhamnose | 1.6766 | 0 | 0.0001 |
| 141 | methionine | 2.3262 | 8.87E-06 | 0.0001 |
| 142 | 2-deoxyerythritol | 1.6134 | 0 | 0.0001 |
| 144 | 5-hydroxy-3-indoleacetic acid | 1.8158 | 0 | 0.0001 |
| 147 | mevalonic acid NIST | 3.6055 | 0 | 0.0001 |
| 148 | Leucine | 2.0177 | 0 | 0.0001 |
| 159 | glutamine | 2.5221 | 0 | 0.0001 |
| 164 | 3-ureidopropionate | 2.6499 | 8.66E-06 | 0.0001 |
| 175 | X267653 | 1.3056 | 0 | 0.0001 |
| 178 | X267687 | 1.5075 | 0 | 0.0001 |
| 185 | X267714 | 1.4435 | 8.48E-06 | 0.0001 |
| 188 | X303152 | 1.8427 | 0 | 0.0001 |
| 191 | X645667 | 1.965 | 4.27E-06 | 0.0001 |
| 192 | X239312 | 1.3987 | 0 | 0.0001 |
| 200 | X267760 | 1.5926 | 0 | 0.0001 |
| 206 | X267723 | 1.4757 | 0 | 0.0001 |
| 216 | X267737 | 3.462 | 7.05E-06 | 0.0001 |
| 217 | X267670 | 2.2725 | 2.26E-06 | 0.0001 |
| 221 | X369589 | 1.623 | 0 | 0.0001 |
| 225 | X227675 | 2.7887 | 8.37E-06 | 0.0001 |
| 232 | X303060 | 3.9839 | 0 | 0.0001 |
| 235 | X267650 | 1.878 | 2.04E-06 | 0.0001 |
| 237 | X636875 | 1.8557 | 0 | 0.0001 |
| 238 | X636805 | 3.4869 | 5.98E-06 | 0.0001 |
| 248 | X636908 | 3.4825 | 0 | 0.0001 |
| 250 | X324627 | 1.9297 | 5.38E-06 | 0.0001 |
| 253 | X642793 | 2.998 | 0 | 0.0001 |
| 264 | X289055 | 2.0607 | 0 | 0.0001 |
| 266 | X228911 | 2.3076 | 0 | 0.0001 |
| 274 | X480050 | 2.8089 | 0 | 0.0001 |
| 277 | X216860 | 3.4146 | 9.83E-06 | 0.0001 |
| 278 | X199463 | 2.8767 | 6.23E-06 | 0.0001 |
| 280 | X267666 | 2.2293 | 0 | 0.0001 |
| 285 | X267701 | 1.8066 | 3E-06 | 0.0001 |
| 300 | X629980 | 2.7162 | 6.21E-06 | 0.0001 |
| 329 | X636909 | 3.0623 | 0 | 0.0001 |
| 332 | X438101 | 4.5833 | 0 | 0.0001 |
| 334 | X231792 | 3.4145 | 0 | 0.0001 |
| 337 | X636886 | 2.0842 | 0 | 0.0001 |
| 351 | X201042 | 2.6099 | 5.14E-06 | 0.0001 |
| 355 | X221571 | 1.4856 | 0 | 0.0001 |
| 357 | X650967 | 2.4456 | 8.6E-06 | 0.0001 |
| 368 | X213143 | 2.0221 | 7.06E-06 | 0.0001 |
| 374 | X234622 | 1.5409 | 0 | 0.0001 |
| 10 | Cysteine | 1.1609 | 0 | 0.0002 |
| 16 | tryptophan | 1.3431 | 0.0001 | 0.0002 |
| 30 | glucose 1 | 1.1805 | 0.0001 | 0.0002 |
| 60 | butyrolactam NIST | 1.6166 | 0 | 0.0002 |
| 74 | mannitol mix spec with histidine | 1.9105 | 0 | 0.0002 |
| 80 | phenylalanine | 1.4809 | 0 | 0.0002 |
| 85 | creatinine | 2.4174 | 0 | 0.0002 |
| 86 | glycerol-3-galactoside | 1.4556 | 0.0001 | 0.0002 |
| 105 | hexuronic acid | 1.5476 | 0 | 0.0002 |
| 127 | beta-alanine | 1.8951 | 0 | 0.0002 |
| 184 | X267923 | 1.5804 | 0 | 0.0002 |
| 190 | X288966 | 1.2582 | 0.0001 | 0.0002 |
| 193 | X636858 | 1.3377 | 0.0001 | 0.0002 |
| 202 | X200541 | 1.6703 | 0 | 0.0002 |
| 218 | X267647 | 4.2189 | 0 | 0.0002 |
| 282 | X636846 | 1.9653 | 0 | 0.0002 |
| 287 | X233005 | 1.6742 | 0.0001 | 0.0002 |
| 295 | X294129 | 1.686 | 0 | 0.0002 |
| 299 | X631981 | 1.3746 | 0 | 0.0002 |
| 318 | X631962 | 3.3336 | 0 | 0.0002 |
| 319 | X381876 | 1.1856 | 0.0001 | 0.0002 |
| 321 | X267765 | 3.3345 | 0 | 0.0002 |
| 336 | X644975 | 1.9897 | 0 | 0.0002 |
| 347 | X632100 | 1.4331 | 0 | 0.0002 |
| 371 | X349922 | 4.3745 | 0.0001 | 0.0002 |
| 379 | X367950 | 1.3335 | 0 | 0.0002 |
| 14 | Xylitol | 4.7979 | 0.0001 | 0.0003 |
| 57 | 3,4-dihydroxyphenylacetic acid | 1.3223 | 0.0001 | 0.0003 |
| 129 | inulotriose 1 | 1.8676 | 0.0001 | 0.0003 |
| 189 | X616746 | 1.3157 | 0.0001 | 0.0003 |
| 236 | X218821 | 1.3822 | 0.0001 | 0.0003 |
| 239 | X320562 | 1.4141 | 0.0001 | 0.0003 |
| 247 | X267756 | 1.2363 | 0.0001 | 0.0003 |
| 289 | X636954 | 1.2713 | 0.0001 | 0.0003 |
| 294 | X303163 | 1.3343 | 0.0001 | 0.0003 |
| 306 | X267649 | 1.2407 | 0.0001 | 0.0003 |
| 310 | X267658 | 1.4384 | 0.0001 | 0.0003 |
| 311 | X236709 | 1.1522 | 0.0001 | 0.0003 |
| 313 | X637204 | 1.0839 | 0.0001 | 0.0003 |
| 345 | X232659 | 1.4557 | 0.0001 | 0.0003 |
| 349 | X479886 | 1.4557 | 0.0001 | 0.0003 |
| 378 | X438099 | -2.9945 | 0.0001 | 0.0003 |
| 11 | 4-hydroxyhippuric acid NIST | 1.3842 | 0.0001 | 0.0004 |
| 31 | 3-hydroxy-3-methylglutaric acid | 1.3263 | 0.0001 | 0.0004 |
| 72 | 3-aminoisobutyric acid | 1.5913 | 0.0001 | 0.0004 |
| 116 | pyrogallol | 1.3758 | 0.0001 | 0.0004 |
| 176 | X267675 | 1.2794 | 0.0001 | 0.0004 |
| 212 | X288019 | 5.8584 | 0.0001 | 0.0004 |
| 286 | X267686 | 1.2119 | 0.0001 | 0.0004 |
| 49 | Arabitol | 5.1597 | 0.0002 | 0.0005 |
| 67 | fucose 1 + rhamnose 2 | 4.5493 | 0.0002 | 0.0005 |
| 182 | X326500 | 5.7652 | 0.0002 | 0.0005 |
| 219 | X225867 | 1.3465 | 0.0002 | 0.0005 |
| 240 | X267730 | 1.3794 | 0.0001 | 0.0005 |
| 245 | X636809 | 1.2177 | 0.0002 | 0.0005 |
| 263 | X229199 | 2.0568 | 0.0002 | 0.0005 |
| 284 | X267715 | 1.2193 | 0.0002 | 0.0005 |
| 326 | X300451 | 1.1828 | 0.0002 | 0.0005 |
| 372 | X339455 | 1.1828 | 0.0002 | 0.0005 |
| 26 | xanthine | 1.1026 | 0.0002 | 0.0006 |
| 61 | sucrose | 1.4479 | 0.0002 | 0.0006 |
| 136 | galacturonic acid 2 | 1.286 | 0.0002 | 0.0006 |
| 230 | X640528 | 1.3365 | 0.0002 | 0.0006 |
| 354 | X382318 | 1.6943 | 0.0002 | 0.0006 |
| 110 | benzoic acid mix spec | -2.73 | 0.0003 | 0.0007 |
| 167 | cholesterol | 1.2391 | 0.0003 | 0.0007 |
| 214 | X268106 | 1.2615 | 0.0002 | 0.0007 |
| 254 | X268093 | 1.216 | 0.0003 | 0.0007 |
| 315 | X304945 | 1.232 | 0.0003 | 0.0007 |
| 335 | X650930 | 1.2015 | 0.0003 | 0.0007 |
| 376 | X485397 | -1.2492 | 0.0003 | 0.0007 |
| 28 | 1-methyladenosine | 1.1012 | 0.0004 | 0.0009 |
| 42 | levoglucosan | 1.2311 | 0.0004 | 0.0009 |
| 227 | X267707 | 0.9117 | 0.0004 | 0.0009 |
| 358 | X644946 | 1.0788 | 0.0003 | 0.0009 |
| 51 | gluconic acid | 0.9078 | 0.0004 | 0.001 |
| 56 | fructose 1 | 1.0924 | 0.0004 | 0.001 |
| 117 | erythronic acid lactone.1 | 1.269 | 0.0004 | 0.001 |
| 152 | palatinitol | 1.7328 | 0.0004 | 0.001 |
| 276 | X231796 | 1.038 | 0.0004 | 0.001 |
| 328 | X368056 | 4.5992 | 0.0004 | 0.001 |
| 346 | X208647 | 1.5757 | 0.0004 | 0.001 |
| 380 | X349036 | 1.4222 | 0.0004 | 0.001 |
| 181 | X647819 | 1.1645 | 0.0004 | 0.0011 |
| 195 | X631980 | 1.1743 | 0.0004 | 0.0011 |
| 343 | X281409 | 1.014 | 0.0005 | 0.0011 |
| 367 | X467949 | 1.1774 | 0.0005 | 0.0011 |
| 46 | glycocyamine major | 1.1085 | 0.0005 | 0.0012 |
| 48 | sorbitol | 1.0519 | 0.0005 | 0.0012 |
| 288 | X228249 | 1.2385 | 0.0005 | 0.0012 |
| 324 | X244467 | 1.2002 | 0.0005 | 0.0012 |
| 66 | histidine | 0.9738 | 0.0006 | 0.0013 |
| 224 | X267652 | 4.8034 | 0.0006 | 0.0013 |
| 327 | X267704 | 1.1236 | 0.0006 | 0.0013 |
| 342 | X328803 | 1.1208 | 0.0006 | 0.0013 |
| 108 | 2-deoxyribonic acid | 1.1339 | 0.0006 | 0.0014 |
| 369 | X651283 | 0.8843 | 0.0006 | 0.0014 |
| 34 | N-acetyl-D-mannosamine major | 0.912 | 0.0007 | 0.0015 |
| 41 | lysine | 0.9346 | 0.0007 | 0.0015 |
| 91 | citrulline | 1.1054 | 0.0007 | 0.0015 |
| 261 | X267755 | 1.0053 | 0.0007 | 0.0016 |
| 293 | X308106 | -1.4015 | 0.0008 | 0.0016 |
| 333 | X241189 | 1.0891 | 0.0008 | 0.0016 |
| 344 | X267691 | 1.0097 | 0.0007 | 0.0016 |
| 373 | X238549 | 1.0071 | 0.0008 | 0.0016 |
| 156 | mannose | 1.1384 | 0.0008 | 0.0017 |
| 157 | asparagine | 1.0409 | 0.0008 | 0.0017 |
| 7 | glycolic acid | 1.0199 | 0.0008 | 0.0018 |
| 107 | erythronic acid lactone | -0.9101 | 0.0009 | 0.0018 |
| 210 | X231544 | 0.9891 | 0.0008 | 0.0018 |
| 100 | quinolinic acid | 0.9898 | 0.0009 | 0.0019 |
| 199 | X368028 | 1.0955 | 0.0009 | 0.0019 |
| 275 | X294547 | 1.0831 | 0.0009 | 0.0019 |
| 38 | 5-hydroxymethyl-2-furoic acid NIST | 1.2263 | 0.001 | 0.002 |
| 78 | quinic acid | 0.9855 | 0.001 | 0.0021 |
| 113 | phosphoethanolamine | 1.0122 | 0.0011 | 0.0022 |
| 170 | arachidic acid | 1.0541 | 0.0011 | 0.0022 |
| 5 | glucose 2 | 1.1467 | 0.0011 | 0.0023 |
| 251 | X267904 | 0.9395 | 0.0011 | 0.0023 |
| 365 | X267937 | 1.0319 | 0.0011 | 0.0023 |
| 44 | 1-methylinosine NIST | 1.0977 | 0.0012 | 0.0024 |
| 187 | X321685 | 0.9751 | 0.0012 | 0.0024 |
| 205 | X408731 | -3.9042 | 0.0012 | 0.0024 |
| 272 | X241141 | 0.9794 | 0.0012 | 0.0024 |
| 353 | X480180 | 0.8801 | 0.0013 | 0.0024 |
| 20 | citramalic acid | 0.9597 | 0.0013 | 0.0025 |
| 109 | (s)-(+)-mandelic acid | 1.0455 | 0.0013 | 0.0025 |
| 138 | inositol allo- | -1.0235 | 0.0014 | 0.0025 |
| 150 | valine TMS1x | 1.0781 | 0.0013 | 0.0025 |
| 283 | X267890 | 1.2204 | 0.0013 | 0.0025 |
| 125 | adenosine | 0.9672 | 0.0014 | 0.0026 |
| 137 | UDP-glucuronic acid | 1.0673 | 0.0014 | 0.0026 |
| 2 | threonic acid 2 | 0.9324 | 0.0015 | 0.0028 |
| 338 | X485388 | 0.9543 | 0.0015 | 0.0028 |
| 37 | cystine minor | 0.937 | 0.0017 | 0.0031 |
| 203 | X647447 | 0.8973 | 0.0018 | 0.0032 |
| 186 | X203765 | 1.0078 | 0.0018 | 0.0033 |
| 267 | X205670 | 1.1076 | 0.0018 | 0.0033 |
| 83 | 6-deoxyglucitol NIST | 1.2301 | 0.002 | 0.0037 |
| 95 | 2-deoxyerythritol NIST | 0.885 | 0.0021 | 0.0037 |
| 121 | 5-methoxytryptamine | 1.2841 | 0.0021 | 0.0038 |
| 382 | X218829 | 1.0959 | 0.0021 | 0.0038 |
| 81 | N-acetylaspartic acid 1 | 0.8488 | 0.0022 | 0.0039 |
| 211 | X348900 | 1.0119 | 0.0024 | 0.0042 |
| 246 | X267774 | 0.8963 | 0.0025 | 0.0043 |
| 307 | X232075 | 0.9507 | 0.0025 | 0.0043 |
| 339 | X483175 | 0.9311 | 0.0025 | 0.0043 |
| 6 | valine | 0.7727 | 0.0026 | 0.0045 |
