# Supplementary material for: Serum and urinary metabolomics and outcomes in cirrhosis
Source: PLoS One. 2019 Sep 27;14(9):e0223061. doi: 10.1371/journal.pone.0223061 (PMC6764675; doi:10.1371/journal.pone.0223061)
Supplement: S7 Table — (DOCX) [file pone.0223061.s016.docx]

| Table S7: Logistic regression serum 90 day HE development | | | | |
| --- | --- | --- | --- | --- |
| index | label | regression_coefficient | p_value | p_values_adjusted |
| 5 | stearic acid | -2.8635 | 5.25E-07 | 0 |
| 6 | pelargonic acid | -3.8022 | 4.54E-06 | 0 |
| 17 | threonine minor | -2.3386 | 7.49E-07 | 0 |
| 40 | glutamine 2TMS | -3.2908 | 2.76E-06 | 0 |
| 43 | isoleucine minor | -4.1872 | 1.26E-06 | 0 |
| 47 | capric acid | -2.6736 | 4.89E-06 | 0 |
| 51 | erythritol | 3.9801 | 4.71E-07 | 0 |
| 72 | methylhexadecanoic acid | -1.7369 | 7.40E-07 | 0 |
| 73 | fructose 1 | 1.4382 | 4.34E-06 | 0 |
| 75 | inosine | -2.9573 | 3.74E-07 | 0 |
| 84 | arabitol | 2.0632 | 3.12E-06 | 0 |
| 85 | xylitol | 4.1645 | 2.15E-06 | 0 |
| 87 | threitol 2 | 4.1113 | 7.51E-07 | 0 |
| 92 | phosphoric acid | -6.2952 | 9.99E-07 | 0 |
| 98 | phenylethylamine | 3.2113 | 9.38E-07 | 0 |
| 104 | maltose 1 | 3.8013 | 2.59E-06 | 0 |
| 110 | glucuronic acid mix spec | 4.4468 | 5.43E-07 | 0 |
| 113 | histidine | -1.8049 | 4.35E-06 | 0 |
| 130 | arachidic acid | -2.6303 | 5.92E-07 | 0 |
| 134 | 4-hydroxyphenylacetic acid | 1.8221 | 2.36E-06 | 0 |
| 144 | 4-hydroxyproline | 1.6712 | 5.59E-06 | 0 |
| 145 | 3-phenyllactic acid | 2.8761 | 4.4E-06 | 0 |
| 146 | nicotinic acid | 8.0748 | 3.68E-06 | 0 |
| 148 | 3,6-anhydrogalactose | 3.7346 | 7.36E-06 | 0 |
| 149 | adipic acid | -1.4932 | 1.84E-06 | 0 |
| 150 | 5-methoxytryptamine | -1.2842 | 1.8E-06 | 0 |
| 159 | parabanic acid NIST | -1.526 | 1.69E-06 | 0 |
| 179 | 2-oxogluconic acid NIST | 3.7749 | 3.5E-06 | 0 |
| 181 | 1-deoxyerythritol | 2.8978 | 5.01E-07 | 0 |
| 185 | galactonic acid | 2.1247 | 3.36E-06 | 0 |
| 186 | ribonic acid | 3.0047 | 1.09E-06 | 0 |
| 187 | 2-ketoisocaproic acid minor | 2.022 | 1.83E-06 | 0 |
| 193 | phthalic acid | 2.175 | 3.97E-06 | 0 |
| 194 | phosphoric acid.1 | -3.5978 | 2.18E-06 | 0 |
| 196 | asparagine 2TMS minor | 5.2645 | 1.33E-06 | 0 |
| 206 | beta-mannosylglycerate minor | 4.1489 | 5.78E-06 | 0 |
| 216 | erythrose | 2.6737 | 7.36E-06 | 0 |
| 234 | homoserine | 2.6739 | 1.56E-06 | 0 |
| 238 | mannose | 5.0428 | 4.9E-06 | 0 |
| 241 | 3,4-dihydroxyphenylacetic acid | 1.563 | 6.87E-06 | 0 |
| 251 | X213253 | -5.4089 | 3.34E-06 | 0 |
| 255 | X455340 | -2.2506 | 4.14E-06 | 0 |
| 284 | X223548 | -1.4371 | 6.64E-07 | 0 |
| 285 | X201862 | -2.196 | 5.9E-06 | 0 |
| 298 | X223505 | 5.4154 | 4.45E-06 | 0 |
| 300 | X597213 | -3.2845 | 4.13E-06 | 0 |
| 301 | X607692 | -2.1814 | 4.96E-07 | 0 |
| 316 | X314770 | 5.6766 | 7.79E-06 | 0 |
| 317 | X216428 | 4.0745 | 3.73E-06 | 0 |
| 320 | X213193 | 4.5453 | 2.39E-06 | 0 |
| 326 | X618071 | -2.9643 | 7.40E-07 | 0 |
| 344 | X227367 | 3.5713 | 4.04E-06 | 0 |
| 347 | X362005 | -4.1348 | 9.21E-07 | 0 |
| 359 | X211952 | -2.0385 | 4.85E-07 | 0 |
| 360 | X208557 | -1.8904 | 5.35E-06 | 0 |
| 362 | X216838 | -3.581 | 1.62E-06 | 0 |
| 363 | X356925 | 3.7474 | 3.28E-06 | 0 |
| 109 | 2-hydroxyvaleric acid | -2.03 | 1.37E-07 | 9.19E-06 |
| 173 | N-acetylglycine NIST | -1.6459 | 1.17E-07 | 9.19E-06 |
| 190 | tartaric acid | -2.0606 | 1.00E-07 | 9.19E-06 |
| 208 | quinolinic acid | 2.2996 | 1.50E-07 | 9.19E-06 |
| 228 | 5-aminovaleric acid lactame | 5.0354 | 1.30E-07 | 9.19E-06 |
| 230 | pantothenic acid | 3.1364 | 6.68E-08 | 9.19E-06 |
| 125 | isorhamnose | 2.37 | 2.11E-07 | 9.39E-06 |
| 198 | 5-hydroxyindole-3-acetic acid NIST | 2.9174 | 2.15E-07 | 9.39E-06 |
| 318 | X640860 | -1.1377 | 2.30E-07 | 9.39E-06 |
| 28 | lauric acid | -1.4101 | 0 | 0.0001 |
| 71 | oxalic acid | -1.5042 | 0 | 0.0001 |
| 91 | fucose 1 + rhamnose 2 | 1.8322 | 0 | 0.0001 |
| 95 | valine TMS1x | -1.8398 | 0 | 0.0001 |
| 115 | fucose | 1.8938 | 0 | 0.0001 |
| 139 | hypoxanthine mix spec with ornithine | -1.4649 | 0 | 0.0001 |
| 152 | pyrophosphate | 7.0608 | 0 | 0.0001 |
| 166 | guanosine | -1.1942 | 0 | 0.0001 |
| 168 | propane-1,3-diol NIST | 1.3733 | 0 | 0.0001 |
| 213 | cyclohexylamine NIST | 3.2337 | 0 | 0.0001 |
| 223 | N-acetyl-D-tryptophan minor2 | 3.9173 | 0 | 0.0001 |
| 231 | inulobiose 2 | 3.3468 | 0 | 0.0001 |
| 236 | 1-methyladenosine | 1.4982 | 0 | 0.0001 |
| 250 | X222169 | 1.2774 | 0 | 0.0001 |
| 254 | X356938 | -5.2835 | 9.83E-06 | 0.0001 |
| 262 | X289052 | -1.1734 | 0 | 0.0001 |
| 267 | X200850 | -1.6731 | 0 | 0.0001 |
| 271 | X612625 | 1.1949 | 0 | 0.0001 |
| 275 | X223597 | 1.6346 | 0 | 0.0001 |
| 287 | X223566 | -6.4386 | 0 | 0.0001 |
| 290 | X228911 | 2.8825 | 0 | 0.0001 |
| 312 | X301325 | 13.6233 | 0 | 0.0001 |
| 315 | X455826 | -1.4154 | 0 | 0.0001 |
| 328 | X497413 | 1.8759 | 0 | 0.0001 |
| 329 | X223629 | 2.3742 | 9.74E-06 | 0.0001 |
| 332 | X484792 | -1.4543 | 0 | 0.0001 |
| 335 | X307915 | -1.7967 | 0 | 0.0001 |
| 336 | X495239 | -1.9599 | 0 | 0.0001 |
| 355 | X224849 | 2.5795 | 9.48E-06 | 0.0001 |
| 357 | X231850 | 1.5135 | 0 | 0.0001 |
| 93 | sucrose | 2.81 | 0 | 0.0002 |
| 107 | pipecolic acid | 5.4889 | 0 | 0.0002 |
| 120 | tagatose 1 | 1.7029 | 0 | 0.0002 |
| 156 | glutaric acid | 1.3097 | 0.0001 | 0.0002 |
| 265 | X207223 | -9.1556 | 0.0001 | 0.0002 |
| 274 | X213143 | 12.4657 | 0 | 0.0002 |
| 291 | X381876 | 1.8479 | 0.0001 | 0.0002 |
| 309 | X199596 | -2.5142 | 0.0001 | 0.0002 |
| 322 | X227352 | 13.4788 | 0 | 0.0002 |
| 345 | X653345 | 1.7849 | 0 | 0.0002 |
| 45 | lysine | -1.4785 | 0.0001 | 0.0003 |
| 74 | methionine | 1.6106 | 0.0001 | 0.0003 |
| 119 | cysteine | -1.4876 | 0.0001 | 0.0003 |
| 133 | threonic acid 1 | -7.7654 | 0.0001 | 0.0003 |
| 161 | 3-aminoisobutyric acid | 1.4514 | 0.0001 | 0.0003 |
| 171 | dodecane | -1.263 | 0.0001 | 0.0003 |
| 297 | X213972 | -7.7788 | 0.0001 | 0.0003 |
| 313 | X273773 | 9.1624 | 0.0001 | 0.0003 |
| 350 | X617556 | 1.4692 | 0.0001 | 0.0003 |
| 24 | proline | 1.6913 | 0.0001 | 0.0004 |
| 199 | methionine sulfoxide minor1 | 1.6283 | 0.0001 | 0.0004 |
| 296 | X438101 | 1.4256 | 0.0001 | 0.0004 |
| 323 | X223871 | 1.5911 | 0.0001 | 0.0004 |
| 26 | serine minor | -1.0702 | 0.0002 | 0.0005 |
| 167 | galactose | 6.9122 | 0.0002 | 0.0005 |
| 203 | cellobiotol | 1.809 | 0.0002 | 0.0005 |
| 245 | 1-methylinosine NIST | 0.9825 | 0.0002 | 0.0005 |
| 121 | elaidic acid | -1.0205 | 0.0002 | 0.0006 |
| 331 | X367932 | -0.9149 | 0.0002 | 0.0006 |
| 337 | X486017 | -1.0189 | 0.0002 | 0.0006 |
| 12 | lactic acid | 0.9328 | 0.0002 | 0.0007 |
| 68 | glutamine dehydrated 2TMS minor | -1.5796 | 0.0002 | 0.0007 |
| 128 | asparagine dehydrated | -0.9287 | 0.0003 | 0.0008 |
| 174 | beta-sitosterol | 1.6873 | 0.0003 | 0.0008 |
| 189 | 3-hydroxypyridine | 1.4734 | 0.0003 | 0.0008 |
| 233 | N-acetyl-D-mannosamine major | 1.0489 | 0.0003 | 0.0008 |
| 117 | mannitol mix spec with histidine | -4.5959 | 0.0003 | 0.0009 |
| 259 | X211979 | -1.013 | 0.0003 | 0.0009 |
| 151 | 1-monoolein | -0.7742 | 0.0004 | 0.001 |
| 101 | alanine 3TMS | -1.1279 | 0.0004 | 0.0011 |
| 158 | 2-deoxyerythritol | 1.2314 | 0.0004 | 0.0011 |
| 310 | X508725 | 2.4392 | 0.0004 | 0.0011 |
| 164 | azelaic acid | -0.6042 | 0.0005 | 0.0012 |
| 288 | X438057 | 1.505 | 0.0005 | 0.0013 |
| 240 | 2-ketoadipic acid | -1.3396 | 0.0006 | 0.0014 |
| 183 | thymine | 1.0094 | 0.0006 | 0.0015 |
| 343 | X486016 | -22.6493 | 0.0006 | 0.0015 |
| 338 | X485397 | -18.765 | 0.0006 | 0.0016 |
| 160 | maleimide | -9.4137 | 0.0007 | 0.0017 |
| 22 | ribitol | -0.8755 | 0.0008 | 0.002 |
| 60 | methanolphosphate | 1.0496 | 0.0008 | 0.002 |
| 147 | glycerol-3-galactoside | 1.0071 | 0.0008 | 0.002 |
| 33 | tocopherol alpha | -1.0288 | 0.0008 | 0.0021 |
| 246 | X408731 | -26.3211 | 0.0009 | 0.0021 |
| 286 | X234717 | 1.1626 | 0.001 | 0.0023 |
| 289 | X223618 | -0.9252 | 0.0009 | 0.0023 |
| 325 | X223625 | 0.9382 | 0.001 | 0.0023 |
| 30 | cholesterol | 1.0858 | 0.001 | 0.0024 |
| 239 | homovanillic and 4-hydroxymandelic acid - mixed spectrum | 1.0866 | 0.001 | 0.0024 |
| 188 | hippuric acid 1TMS | 0.8846 | 0.0012 | 0.0029 |
| 305 | X499123 | -2.8369 | 0.0013 | 0.003 |
| 81 | N-acetylglutamate | -1.0416 | 0.0014 | 0.0033 |
| 324 | X309540 | -0.8139 | 0.0015 | 0.0035 |
| 36 | ornithine 4TMS | -1.1227 | 0.0016 | 0.0036 |
| 11 | alanine | 0.8744 | 0.0016 | 0.0037 |
| 137 | phenylacetic acid | 1.029 | 0.0017 | 0.0038 |
| 58 | glutamine dehydrated | -0.9698 | 0.0019 | 0.0042 |
| 361 | X428311 | -0.7369 | 0.002 | 0.0044 |
| 79 | glyceric acid | 0.9853 | 0.002 | 0.0045 |
| 154 | 1-monostearin | 1.0275 | 0.0021 | 0.0046 |
| 165 | alpha ketoglutaric acid | 1.0729 | 0.0021 | 0.0046 |
| 224 | lactobionic acid | -0.7678 | 0.0025 | 0.0055 |
| 90 | taurine | -0.9996 | 0.0028 | 0.006 |
| 212 | cysteine-glycine | -0.7054 | 0.0027 | 0.006 |
| 50 | 1,5-anhydroglucitol | -1.1834 | 0.003 | 0.0063 |
| 100 | N-methylalanine | 0.8494 | 0.003 | 0.0063 |
| 170 | pentadecanoic acid | 0.8833 | 0.0032 | 0.0067 |
| 172 | pyruvic acid | -1.1958 | 0.0032 | 0.0067 |
| 314 | X465393 | -0.9167 | 0.0033 | 0.0068 |
| 277 | X199794 | -1.2625 | 0.0033 | 0.0069 |
| 127 | idonic acid NIST | -0.9196 | 0.0036 | 0.0074 |
| 207 | 3-aminoisobutyric acid 1 | 1.0398 | 0.0036 | 0.0075 |
| 48 | caprylic acid | -0.6077 | 0.0037 | 0.0076 |
| 129 | benzoic acid mix spec | -24.0789 | 0.0038 | 0.0077 |
| 220 | 2,3-dihydroxybutanoic acid NIST | 0.8025 | 0.0039 | 0.008 |
| 176 | isolinoleic acid NIST | 0.7447 | 0.004 | 0.0081 |
| 215 | saccharic acid | 1.196 | 0.0041 | 0.0083 |
| 175 | salicylic acid | -0.5216 | 0.0042 | 0.0084 |
| 78 | glycolic acid | -0.9627 | 0.0056 | 0.011 |
| 122 | biuret | -0.7904 | 0.0055 | 0.011 |
| 141 | inositol allo- | 1.2175 | 0.0057 | 0.0111 |
| 15 | fructose 2 | 0.6592 | 0.0058 | 0.0112 |
| 54 | glutamate TMS2x | -0.6958 | 0.0058 | 0.0112 |
| 340 | X537746 | 0.7522 | 0.0057 | 0.0112 |
| 88 | shikimic acid | -0.7322 | 0.0059 | 0.0113 |
| 180 | dihydro-3-coumaric acid | -0.6698 | 0.0063 | 0.0118 |
| 219 | beta-alanine minor | 0.863 | 0.0062 | 0.0118 |
| 257 | X268506 | -0.7532 | 0.0062 | 0.0118 |
| 346 | X612627 | 2.7579 | 0.0063 | 0.0119 |
| 10 | glucose 2 | -0.8442 | 0.0065 | 0.0122 |
| 96 | 2-deoxytetronic acid NIST | -0.7306 | 0.0066 | 0.0122 |
| 293 | X537868 | -2.218 | 0.0068 | 0.0126 |
| 351 | X486054 | 1.0909 | 0.007 | 0.0129 |
| 226 | trehalose | 0.7943 | 0.0078 | 0.0144 |
| 205 | shikimic acid.1 | -0.7147 | 0.0084 | 0.0152 |
| 356 | X566268 | 0.8459 | 0.0083 | 0.0152 |
| 23 | glutamine | -0.7638 | 0.0086 | 0.0156 |
| 204 | 5-hydroxymethyl-2-furoic acid NIST | 0.771 | 0.0091 | 0.0164 |
| 295 | X199942 | 0.821 | 0.0093 | 0.0166 |
| 201 | N-acetyl-D-hexosamine | 0.6006 | 0.0097 | 0.0172 |
| 163 | threose meox2 | 0.6157 | 0.0098 | 0.0174 |
| 34 | trans-4-hydroxyproline | -0.6993 | 0.0103 | 0.0181 |
| 278 | X339455 | -0.6497 | 0.0103 | 0.0181 |
| 319 | X277432 | -0.7653 | 0.0104 | 0.0182 |
| 20 | phenylalanine TMS1x | -0.769 | 0.0107 | 0.0186 |
| 55 | glucose 1 | 0.6583 | 0.0113 | 0.0195 |
| 225 | 3-methoxytyrosine NIST | 0.5788 | 0.0121 | 0.0209 |
| 131 | methionine sulfoxide major | -0.6008 | 0.0124 | 0.0213 |
| 16 | hydroxylamine | -0.7248 | 0.013 | 0.0223 |
| 192 | 5-hydroxynorvaline NIST | 0.7874 | 0.0142 | 0.0242 |
| 258 | X225446 | 0.7082 | 0.016 | 0.0271 |
| 367 | X270508 | 0.8119 | 0.0163 | 0.0274 |
| 82 | asparagine minor 2 | -0.9031 | 0.0184 | 0.0308 |
| 52 | arachidonic acid isomer | -0.5781 | 0.0192 | 0.032 |
| 3 | tryptophan | -0.6064 | 0.0201 | 0.0334 |
| 365 | X225430 | 0.5816 | 0.0213 | 0.0352 |
| 14 | serine | 0.6464 | 0.022 | 0.0363 |
| 178 | aconitic acid | -0.5443 | 0.0226 | 0.037 |
| 311 | X565868 | 0.6259 | 0.0261 | 0.0426 |
| 248 | X199786 | -0.6784 | 0.0269 | 0.0437 |
| 38 | succinic acid | -0.5937 | 0.0273 | 0.0442 |
| 112 | uridine | -0.5821 | 0.0301 | 0.0484 |
