# Supplementary material for: Serum and urinary metabolomics and outcomes in cirrhosis
Source: PLoS One. 2019 Sep 27;14(9):e0223061. doi: 10.1371/journal.pone.0223061 (PMC6764675; doi:10.1371/journal.pone.0223061)
Supplement: S8 Table — (DOCX) [file pone.0223061.s017.docx]

| Table S8: Urine metabolites Logistic Regression HE | | | | |
| --- | --- | --- | --- | --- |
| index | label | regression_coefficient | p_value | p_values_adjusted |
| 9 | isothreonic acid | 1.4486 | 3.17E-06 | 0 |
| 14 | xylitol | 4.0152 | 3.64E-06 | 0 |
| 18 | indole-3-acetate | 1.733 | 3.68E-06 | 0 |
| 23 | tyrosine mz147 missing | 1.4733 | 4.89E-06 | 0 |
| 29 | arabinose | 1.5844 | 2.54E-06 | 0 |
| 30 | glucose 1 | 1.074 | 6.06E-06 | 0 |
| 43 | indole-3-lactate | 2.1797 | 8.97E-07 | 0 |
| 47 | ethanolamine | 1.4524 | 1.21E-06 | 0 |
| 49 | arabitol | 3.2284 | 1.38E-06 | 0 |
| 54 | 4-hydroxyphenylacetic acid | 2.8554 | 1.55E-06 | 0 |
| 67 | fucose 1 + rhamnose 2 | 2.8286 | 1.9E-06 | 0 |
| 68 | cellobiotol | 1.6666 | 3.76E-06 | 0 |
| 69 | urocanic acid | 1.3785 | 8.72E-06 | 0 |
| 70 | ribose | 1.5059 | 5.14E-06 | 0 |
| 74 | mannitol mix spec with histidine | 1.3199 | 5.33E-06 | 0 |
| 79 | saccharic acid | 1.4695 | 2.6E-06 | 0 |
| 80 | phenylalanine | 1.4365 | 9.61E-06 | 0 |
| 82 | fucose | 1.5179 | 5.05E-06 | 0 |
| 85 | creatinine | 2.2676 | 5.15E-06 | 0 |
| 89 | hypoxanthine mix spec with ornithine | 1.3185 | 9.63E-06 | 0 |
| 92 | N-acetyl-D-mannosamine 3 | 1.6192 | 2.22E-06 | 0 |
| 94 | 3-hydroxy-3-indoleacetic acid | 1.56 | 6E-06 | 0 |
| 96 | xylulose NIST | 1.6159 | 4.95E-06 | 0 |
| 99 | 1,2-anhydro-myo-inositol NIST | 2.1566 | 1.54E-06 | 0 |
| 103 | azelaic acid | 1.528 | 2.62E-06 | 0 |
| 114 | N-acetyl-D-hexosamine | 1.7074 | 3.86E-06 | 0 |
| 115 | 5'-deoxy-5'-methylthioadenosine | 1.6439 | 2.03E-06 | 0 |
| 118 | 2,3-dihydroxybutanoic acid NIST | 2.026 | 2.64E-06 | 0 |
| 120 | glucuronic acid mix spec | 2.053 | 1.06E-07 | 0 |
| 122 | lyxose minor | 1.9182 | 5.90E-07 | 0 |
| 130 | homovanillic and 4-hydroxymandelic acid - mixed spectrum | 1.9867 | 1.09E-06 | 0 |
| 131 | glutamic acid | 2.1786 | 1.86E-06 | 0 |
| 141 | methionine | 2.3281 | 1.34E-06 | 0 |
| 142 | 2-deoxyerythritol | 1.2269 | 9.51E-06 | 0 |
| 144 | 5-hydroxy-3-indoleacetic acid | 1.5812 | 5.68E-06 | 0 |
| 147 | mevalonic acid NIST | 2.1109 | 5.06E-06 | 0 |
| 148 | leucine | 1.8932 | 2.55E-06 | 0 |
| 159 | glutamine | 2.2783 | 3.81E-06 | 0 |
| 164 | 3-ureidopropionate | 1.8559 | 4.03E-06 | 0 |
| 182 | X326500 | 2.6092 | 3.45E-07 | 0 |
| 185 | X267714 | 1.3404 | 3.8E-06 | 0 |
| 188 | X303152 | 1.9482 | 1.71E-06 | 0 |
| 191 | X645667 | 1.5362 | 4.4E-06 | 0 |
| 192 | X239312 | 1.1648 | 6.9E-06 | 0 |
| 200 | X267760 | 1.2861 | 0 | 0 |
| 212 | X288019 | 4.4444 | 2.67E-06 | 0 |
| 216 | X267737 | 2.2354 | 6.79E-07 | 0 |
| 217 | X267670 | 1.9974 | 1.2E-06 | 0 |
| 218 | X267647 | 3.6143 | 4.97E-07 | 0 |
| 221 | X369589 | 1.398 | 6.4E-06 | 0 |
| 225 | X227675 | 2.7224 | 1.5E-06 | 0 |
| 232 | X303060 | 3.3359 | 2.71E-06 | 0 |
| 235 | X267650 | 1.3831 | 1.56E-06 | 0 |
| 237 | X636875 | 1.8081 | 1.73E-06 | 0 |
| 238 | X636805 | 2.1739 | 6.63E-07 | 0 |
| 248 | X636908 | 2.1423 | 2.19E-06 | 0 |
| 250 | X324627 | 1.5569 | 3.02E-06 | 0 |
| 253 | X642793 | 2.1866 | 1.49E-06 | 0 |
| 266 | X228911 | 1.9883 | 8.97E-07 | 0 |
| 274 | X480050 | 2.0794 | 2.75E-06 | 0 |
| 277 | X216860 | 2.5051 | 4.78E-07 | 0 |
| 278 | X199463 | 2.6268 | 2.15E-06 | 0 |
| 280 | X267666 | 1.8872 | 9.4E-06 | 0 |
| 285 | X267701 | 1.4807 | 2.8E-06 | 0 |
| 294 | X303163 | 1.3267 | 9.99E-06 | 0 |
| 295 | X294129 | 1.5548 | 6.06E-06 | 0 |
| 299 | X631981 | 1.3524 | 5.83E-06 | 0 |
| 300 | X629980 | 1.7188 | 1.99E-06 | 0 |
| 321 | X267765 | 2.78 | 6.9E-06 | 0 |
| 328 | X368056 | 2.8248 | 3.32E-07 | 0 |
| 329 | X636909 | 2.1964 | 4.42E-06 | 0 |
| 332 | X438101 | 4.8169 | 2.73E-06 | 0 |
| 334 | X231792 | 2.667 | 8.06E-07 | 0 |
| 337 | X636886 | 1.4653 | 6.48E-06 | 0 |
| 351 | X201042 | 2.1919 | 3.92E-07 | 0 |
| 355 | X221571 | 1.5482 | 4.55E-06 | 0 |
| 357 | X650967 | 2.0501 | 1.71E-06 | 0 |
| 368 | X213143 | 1.4779 | 8.35E-06 | 0 |
| 371 | X349922 | 2.5263 | 1.24E-06 | 0 |
| 374 | X234622 | 1.2912 | 5.38E-06 | 0 |
| 10 | cystine | 1.0084 | 0 | 0.0001 |
| 13 | erythritol | 4.9826 | 0 | 0.0001 |
| 16 | tryptophan | 1.1655 | 0 | 0.0001 |
| 57 | 3,4-dihydroxyphenylacetic acid | 1.1545 | 0 | 0.0001 |
| 60 | butyrolactam NIST | 1.3429 | 0 | 0.0001 |
| 61 | sucrose | 1.4791 | 0 | 0.0001 |
| 62 | isocitric acid | 1.1448 | 0 | 0.0001 |
| 72 | 3-aminoisobutyric acid | 1.8249 | 0 | 0.0001 |
| 86 | glycerol-3-galactoside | 1.1849 | 0 | 0.0001 |
| 93 | 5-aminovaleric acid lactame | 1.9196 | 0 | 0.0001 |
| 98 | 2-hydroxyadipic acid | 1.4903 | 0 | 0.0001 |
| 119 | serine minor | 1.1746 | 0 | 0.0001 |
| 127 | beta-alanine | 1.3579 | 0 | 0.0001 |
| 132 | galactinol major 2 | 1.3204 | 0 | 0.0001 |
| 134 | isorhamnose | 1.3535 | 0 | 0.0001 |
| 175 | X267653 | 1.0562 | 0 | 0.0001 |
| 178 | X267687 | 1.2149 | 0 | 0.0001 |
| 184 | X267923 | 1.3834 | 0 | 0.0001 |
| 190 | X288966 | 1.0447 | 0 | 0.0001 |
| 193 | X636858 | 1.1265 | 0 | 0.0001 |
| 202 | X200541 | 1.171 | 0 | 0.0001 |
| 206 | X267723 | 1.1942 | 0 | 0.0001 |
| 214 | X268106 | 1.1418 | 0 | 0.0001 |
| 224 | X267652 | 3.9658 | 0 | 0.0001 |
| 230 | X640528 | 1.4455 | 0 | 0.0001 |
| 236 | X218821 | 1.2405 | 0 | 0.0001 |
| 239 | X320562 | 1.414 | 0 | 0.0001 |
| 240 | X267730 | 1.2687 | 0 | 0.0001 |
| 264 | X289055 | 1.3806 | 0 | 0.0001 |
| 282 | X636846 | 1.4443 | 0 | 0.0001 |
| 306 | X267649 | 1.0323 | 0 | 0.0001 |
| 318 | X631962 | 2.7079 | 0 | 0.0001 |
| 336 | X644975 | 1.328 | 0 | 0.0001 |
| 345 | X232659 | 1.3574 | 0 | 0.0001 |
| 347 | X632100 | 1.2396 | 0 | 0.0001 |
| 358 | X644946 | 1.1893 | 0 | 0.0001 |
| 379 | X367950 | 1.0561 | 0 | 0.0001 |
| 11 | 4-hydroxyhippuric acid NIST | 1.0374 | 0.0001 | 0.0002 |
| 42 | levoglucosan | 1.1559 | 0 | 0.0002 |
| 116 | pyrogallol | 1.114 | 0.0001 | 0.0002 |
| 189 | X616746 | 1.0715 | 0.0001 | 0.0002 |
| 247 | X267756 | 1.0065 | 0.0001 | 0.0002 |
| 276 | X231796 | 1.0694 | 0.0001 | 0.0002 |
| 286 | X267686 | 0.9951 | 0.0001 | 0.0002 |
| 287 | X233005 | 1.2376 | 0.0001 | 0.0002 |
| 310 | X267658 | 1.1644 | 0.0001 | 0.0002 |
| 319 | X381876 | 1.025 | 0.0001 | 0.0002 |
| 326 | X300451 | 1.0636 | 0.0001 | 0.0002 |
| 335 | X650930 | 1.1401 | 0.0001 | 0.0002 |
| 46 | glycocyamine major | 1.2249 | 0.0001 | 0.0003 |
| 105 | hexuronic acid | 1.0662 | 0.0001 | 0.0003 |
| 227 | X267707 | 0.9015 | 0.0001 | 0.0003 |
| 245 | X636809 | 1.0029 | 0.0001 | 0.0003 |
| 263 | X229199 | 1.5486 | 0.0001 | 0.0003 |
| 289 | X636954 | 1.038 | 0.0001 | 0.0003 |
| 313 | X637204 | 0.887 | 0.0001 | 0.0003 |
| 346 | X208647 | 1.4445 | 0.0001 | 0.0003 |
| 376 | X485397 | -1.2192 | 0.0001 | 0.0003 |
| 129 | inulotriose 1 | 1.2185 | 0.0001 | 0.0004 |
| 284 | X267715 | 1.0603 | 0.0002 | 0.0004 |
| 378 | X438099 | -1.818 | 0.0001 | 0.0004 |
| 26 | xanthine | 0.9577 | 0.0002 | 0.0005 |
| 28 | 1-methyladenosine | 0.9597 | 0.0002 | 0.0005 |
| 31 | 3-hydroxy-3-methylglutaric acid | 0.949 | 0.0002 | 0.0005 |
| 51 | gluconic acid | 0.8911 | 0.0002 | 0.0005 |
| 121 | 5-methoxytryptamine | 1.4954 | 0.0002 | 0.0005 |
| 170 | arachidic acid | 1.1173 | 0.0002 | 0.0005 |
| 311 | X236709 | 0.9205 | 0.0002 | 0.0005 |
| 342 | X328803 | 1.0082 | 0.0002 | 0.0005 |
| 343 | X281409 | 1.0003 | 0.0002 | 0.0005 |
| 344 | X267691 | 1.0343 | 0.0002 | 0.0005 |
| 38 | 5-hydroxymethyl-2-furoic acid NIST | 1.056 | 0.0003 | 0.0006 |
| 56 | fructose 1 | 0.9249 | 0.0002 | 0.0006 |
| 91 | citrulline | 0.9969 | 0.0002 | 0.0006 |
| 176 | X267675 | 0.9132 | 0.0003 | 0.0006 |
| 205 | X408731 | -3.992 | 0.0002 | 0.0006 |
| 41 | lysine | 0.9294 | 0.0003 | 0.0007 |
| 66 | histidine | 0.9315 | 0.0003 | 0.0007 |
| 100 | quinolinic acid | 0.882 | 0.0003 | 0.0007 |
| 110 | benzoic acid mix spec | -2.4571 | 0.0003 | 0.0007 |
| 125 | adenosine | 0.9864 | 0.0003 | 0.0007 |
| 195 | X631980 | 0.8933 | 0.0003 | 0.0007 |
| 210 | X231544 | 0.9195 | 0.0003 | 0.0007 |
| 251 | X267904 | 0.9038 | 0.0003 | 0.0007 |
| 354 | X382318 | 1.2001 | 0.0003 | 0.0007 |
| 2 | threonic acid 2 | 0.8936 | 0.0003 | 0.0008 |
| 219 | X225867 | 0.9603 | 0.0004 | 0.0008 |
| 367 | X467949 | 0.9333 | 0.0004 | 0.0008 |
| 382 | X218829 | 1.1461 | 0.0003 | 0.0008 |
| 34 | N-acetyl-D-mannosamine major | 0.8197 | 0.0004 | 0.0009 |
| 137 | UDP-glucuronic acid | 1.0152 | 0.0004 | 0.0009 |
| 157 | asparagine | 0.9738 | 0.0004 | 0.0009 |
| 181 | X647819 | 0.9607 | 0.0004 | 0.0009 |
| 261 | X267755 | 0.8856 | 0.0004 | 0.0009 |
| 373 | X238549 | 0.915 | 0.0004 | 0.0009 |
| 288 | X228249 | 1.0163 | 0.0005 | 0.001 |
| 324 | X244467 | 0.9357 | 0.0005 | 0.001 |
| 380 | X349036 | 0.9753 | 0.0005 | 0.001 |
| 113 | phosphoethanolamine | 0.8869 | 0.0005 | 0.0011 |
| 117 | erythronic acid lactone.1 | 0.9761 | 0.0005 | 0.0011 |
| 187 | X321685 | 0.8511 | 0.0005 | 0.0011 |
| 167 | cholesterol | 0.9518 | 0.0006 | 0.0012 |
| 349 | X479886 | 0.8569 | 0.0006 | 0.0012 |
| 372 | X339455 | 0.8616 | 0.0006 | 0.0013 |
| 83 | 6-deoxyglucitol NIST | 1.0392 | 0.0007 | 0.0014 |
| 171 | lauric acid | 0.8792 | 0.0007 | 0.0014 |
| 283 | X267890 | 1.0119 | 0.0007 | 0.0014 |
| 315 | X304945 | 0.9885 | 0.0007 | 0.0015 |
| 365 | X267937 | 0.8368 | 0.0008 | 0.0015 |
| 78 | quinic acid | 0.8349 | 0.0008 | 0.0016 |
| 108 | 2-deoxyribonic acid | 0.8535 | 0.0008 | 0.0016 |
| 254 | X268093 | 0.9072 | 0.0009 | 0.0017 |
| 369 | X651283 | 0.729 | 0.0009 | 0.0017 |
| 203 | X647447 | 0.7847 | 0.0009 | 0.0018 |
| 37 | cystine minor | 0.8266 | 0.001 | 0.002 |
| 44 | 1-methylinosine NIST | 0.9523 | 0.0011 | 0.0021 |
| 156 | mannose | 0.8859 | 0.0012 | 0.0023 |
| 333 | X241189 | 0.7885 | 0.0012 | 0.0023 |
| 6 | valine | 0.7757 | 0.0012 | 0.0024 |
| 81 | N-acetylaspartic acid 1 | 0.7781 | 0.0012 | 0.0024 |
| 338 | X485388 | 0.7423 | 0.0013 | 0.0024 |
| 152 | palatinitol | 1.0887 | 0.0014 | 0.0026 |
| 198 | X223625 | 0.8362 | 0.0014 | 0.0026 |
| 267 | X205670 | 0.8606 | 0.0014 | 0.0026 |
| 366 | X636861 | 0.8383 | 0.0014 | 0.0026 |
| 20 | citramalic acid | 0.7677 | 0.0015 | 0.0027 |
| 186 | X203765 | 0.8019 | 0.0014 | 0.0027 |
| 275 | X294547 | 0.7729 | 0.0015 | 0.0027 |
| 48 | sorbitol | 0.734 | 0.0015 | 0.0028 |
| 109 | (s)-(+)-mandelic acid | 0.8785 | 0.0016 | 0.0029 |
| 124 | alpha ketoglutaric acid | 0.7945 | 0.0016 | 0.0029 |
| 136 | galacturonic acid 2 | 0.7736 | 0.0016 | 0.0029 |
| 353 | X480180 | 0.6624 | 0.0019 | 0.0035 |
| 4 | palmitic acid | 0.806 | 0.002 | 0.0036 |
| 107 | erythronic acid lactone | -0.6722 | 0.0021 | 0.0037 |
| 204 | X644906 | 0.7219 | 0.0021 | 0.0037 |
| 292 | X321716 | 1.0003 | 0.0023 | 0.004 |
| 95 | 2-deoxyerythritol NIST | 0.6714 | 0.0025 | 0.0044 |
| 76 | 2-hydroxy-2-methylbutanoic acid | -0.9762 | 0.0026 | 0.0045 |
| 207 | X267926 | 0.7088 | 0.0026 | 0.0045 |
| 104 | propane-1,3-diol NIST | 0.853 | 0.0028 | 0.0048 |
