# Supplementary material for: Serum and urinary metabolomics and outcomes in cirrhosis
Source: PLoS One. 2019 Sep 27;14(9):e0223061. doi: 10.1371/journal.pone.0223061 (PMC6764675; doi:10.1371/journal.pone.0223061)
Supplement: S9 Table — (DOCX) [file pone.0223061.s018.docx]

| Table S9: Serum 90 day death logistic regression | | | | |
| --- | --- | --- | --- | --- |
| index | label | regression_coefficient | p_value | p_values_adjusted |
| 5 | stearic acid | -2.8589 | 4.54E-07 | 0 |
| 6 | pelargonic acid | -3.8773 | 3.67E-06 | 0 |
| 17 | threonine minor | -2.3577 | 6.51E-07 | 0 |
| 40 | glutamine 2TMS | -3.3199 | 2.54E-06 | 0 |
| 43 | isoleucine minor | -4.2053 | 1.06E-06 | 0 |
| 47 | capric acid | -2.7028 | 3E-06 | 0 |
| 51 | erythritol | 4.0056 | 4.45E-07 | 0 |
| 72 | methylhexadecanoic acid | -1.7634 | 8.06E-07 | 0 |
| 73 | fructose 1 | 1.4439 | 3.37E-06 | 0 |
| 75 | inosine | -2.9625 | 3.73E-07 | 0 |
| 84 | arabitol | 2.088 | 2.08E-06 | 0 |
| 85 | xylitol | 4.216 | 2.04E-06 | 0 |
| 87 | threitol 2 | 4.1503 | 7.07E-07 | 0 |
| 92 | phosphoric acid | -6.3656 | 8.39E-07 | 0 |
| 98 | phenylethylamine | 3.2363 | 7.33E-07 | 0 |
| 104 | maltose 1 | 3.8537 | 2.34E-06 | 0 |
| 110 | glucuronic acid mix spec | 4.5012 | 5.32E-07 | 0 |
| 113 | histidine | -1.7528 | 3.66E-06 | 0 |
| 130 | arachidic acid | -2.6795 | 4.40E-07 | 0 |
| 134 | 4-hydroxyphenylacetic acid | 1.9104 | 1.34E-06 | 0 |
| 144 | 4-hydroxyproline | 1.691 | 3.73E-06 | 0 |
| 145 | 3-phenyllactic acid | 2.8672 | 4.57E-06 | 0 |
| 146 | nicotinic acid | 8.1015 | 3.34E-06 | 0 |
| 148 | 3,6-anhydrogalactose | 3.7317 | 7.51E-06 | 0 |
| 150 | 5-methoxytryptamine | -1.3059 | 1.18E-06 | 0 |
| 159 | parabanic acid NIST | -1.5206 | 1.23E-06 | 0 |
| 179 | 2-oxogluconic acid NIST | 3.8569 | 2.89E-06 | 0 |
| 181 | 1-deoxyerythritol | 2.8307 | 5.68E-07 | 0 |
| 185 | galactonic acid | 2.1699 | 2.52E-06 | 0 |
| 186 | ribonic acid | 3.0091 | 7.52E-07 | 0 |
| 187 | 2-ketoisocaproic acid minor | 2.0744 | 1.05E-06 | 0 |
| 193 | phthalic acid | 2.1947 | 3.18E-06 | 0 |
| 194 | phosphoric acid.1 | -3.6022 | 2.16E-06 | 0 |
| 196 | asparagine 2TMS minor | 5.266 | 1.31E-06 | 0 |
| 206 | beta-mannosylglycerate minor | 4.1979 | 5.89E-06 | 0 |
| 216 | erythrose | 2.7192 | 5.23E-06 | 0 |
| 231 | inulobiose 2 | 3.4134 | 7.44E-06 | 0 |
| 234 | homoserine | 2.6816 | 1.36E-06 | 0 |
| 236 | 1-methyladenosine | 1.5216 | 7.52E-06 | 0 |
| 238 | mannose | 5.0691 | 3.62E-06 | 0 |
| 241 | 3,4-dihydroxyphenylacetic acid | 1.6523 | 3.86E-06 | 0 |
| 251 | X213253 | -5.4234 | 2.96E-06 | 0 |
| 254 | X356938 | -5.2939 | 8.89E-06 | 0 |
| 255 | X455340 | -2.2843 | 2.41E-06 | 0 |
| 284 | X223548 | -1.4565 | 5.92E-07 | 0 |
| 285 | X201862 | -2.202 | 6.78E-06 | 0 |
| 298 | X223505 | 5.4458 | 3.79E-06 | 0 |
| 300 | X597213 | -3.3836 | 2.68E-06 | 0 |
| 301 | X607692 | -2.1972 | 4.29E-07 | 0 |
| 315 | X455826 | -1.4191 | 9.59E-06 | 0 |
| 316 | X314770 | 5.6654 | 7.39E-06 | 0 |
| 317 | X216428 | 4.0776 | 2.82E-06 | 0 |
| 320 | X213193 | 4.5788 | 1.81E-06 | 0 |
| 326 | X618071 | -3.0525 | 5.04E-07 | 0 |
| 329 | X223629 | 2.4093 | 8.08E-06 | 0 |
| 335 | X307915 | -1.8338 | 7.53E-06 | 0 |
| 344 | X227367 | 3.5824 | 2.92E-06 | 0 |
| 347 | X362005 | -4.0998 | 6.06E-07 | 0 |
| 355 | X224849 | 2.7495 | 5.68E-06 | 0 |
| 359 | X211952 | -2.0619 | 3.52E-07 | 0 |
| 360 | X208557 | -1.9359 | 3.35E-06 | 0 |
| 362 | X216838 | -3.9214 | 3.25E-06 | 0 |
| 363 | X356925 | 3.7477 | 3.11E-06 | 0 |
| 109 | 2-hydroxyvaleric acid | -2.0541 | 9.74E-08 | 8.13E-06 |
| 125 | isorhamnose | 2.3433 | 1.58E-07 | 8.13E-06 |
| 173 | N-acetylglycine NIST | -1.6729 | 8.64E-08 | 8.13E-06 |
| 190 | tartaric acid | -2.0804 | 6.79E-08 | 8.13E-06 |
| 198 | 5-hydroxyindole-3-acetic acid NIST | 2.9352 | 1.95E-07 | 8.13E-06 |
| 208 | quinolinic acid | 2.3041 | 1.14E-07 | 8.13E-06 |
| 228 | 5-aminovaleric acid lactame | 5.1317 | 1.14E-07 | 8.13E-06 |
| 230 | pantothenic acid | 3.3202 | 1.73E-07 | 8.13E-06 |
| 318 | X640860 | -1.1397 | 1.99E-07 | 8.13E-06 |
| 71 | oxalic acid | -1.5252 | 0 | 0.0001 |
| 91 | fucose 1 + rhamnose 2 | 1.8644 | 0 | 0.0001 |
| 95 | valine TMS1x | -1.8863 | 0 | 0.0001 |
| 107 | pipecolic acid | 5.5122 | 0 | 0.0001 |
| 115 | fucose | 1.9059 | 0 | 0.0001 |
| 139 | hypoxanthine mix spec with ornithine | -1.4433 | 0 | 0.0001 |
| 149 | adipic acid | -1.3269 | 0 | 0.0001 |
| 152 | pyrophosphate | 7.164 | 0 | 0.0001 |
| 166 | guanosine | -1.2066 | 0 | 0.0001 |
| 168 | propane-1,3-diol NIST | 1.4018 | 0 | 0.0001 |
| 213 | cyclohexylamine NIST | 3.2662 | 0 | 0.0001 |
| 223 | N-acetyl-D-tryptophan minor2 | 3.871 | 0 | 0.0001 |
| 250 | X222169 | 1.2889 | 0 | 0.0001 |
| 262 | X289052 | -1.1124 | 0 | 0.0001 |
| 267 | X200850 | -1.63 | 0 | 0.0001 |
| 271 | X612625 | 1.2064 | 0 | 0.0001 |
| 274 | X213143 | 12.6488 | 0 | 0.0001 |
| 275 | X223597 | 1.5149 | 0 | 0.0001 |
| 287 | X223566 | -6.4417 | 0 | 0.0001 |
| 290 | X228911 | 2.8835 | 0 | 0.0001 |
| 312 | X301325 | 13.6449 | 0 | 0.0001 |
| 328 | X497413 | 1.9162 | 0 | 0.0001 |
| 332 | X484792 | -1.4727 | 0 | 0.0001 |
| 336 | X495239 | -2.1042 | 0 | 0.0001 |
| 345 | X653345 | 1.8328 | 0 | 0.0001 |
| 357 | X231850 | 1.4916 | 0 | 0.0001 |
| 28 | lauric acid | -1.3906 | 0 | 0.0002 |
| 74 | methionine | 1.6451 | 0.0001 | 0.0002 |
| 93 | sucrose | 2.8054 | 0 | 0.0002 |
| 120 | tagatose 1 | 1.7145 | 0 | 0.0002 |
| 156 | glutaric acid | 1.3237 | 0.0001 | 0.0002 |
| 265 | X207223 | -9.0504 | 0.0001 | 0.0002 |
| 291 | X381876 | 1.8691 | 0.0001 | 0.0002 |
| 297 | X213972 | -7.8689 | 0.0001 | 0.0002 |
| 309 | X199596 | -2.5467 | 0 | 0.0002 |
| 322 | X227352 | 13.4793 | 0 | 0.0002 |
| 45 | lysine | -1.4319 | 0.0001 | 0.0003 |
| 119 | cysteine | -1.4209 | 0.0001 | 0.0003 |
| 161 | 3-aminoisobutyric acid | 1.4699 | 0.0001 | 0.0003 |
| 199 | methionine sulfoxide minor1 | 1.6326 | 0.0001 | 0.0003 |
| 203 | cellobiotol | 1.9046 | 0.0001 | 0.0003 |
| 296 | X438101 | 1.4574 | 0.0001 | 0.0003 |
| 313 | X273773 | 8.9745 | 0.0001 | 0.0003 |
| 323 | X223871 | 1.6133 | 0.0001 | 0.0003 |
| 24 | proline | 1.7156 | 0.0001 | 0.0004 |
| 26 | serine minor | -1.0975 | 0.0001 | 0.0004 |
| 133 | threonic acid 1 | -7.5072 | 0.0001 | 0.0004 |
| 167 | galactose | 4.3455 | 0.0001 | 0.0004 |
| 171 | dodecane | -1.1955 | 0.0001 | 0.0004 |
| 245 | 1-methylinosine NIST | 1.0031 | 0.0001 | 0.0004 |
| 337 | X486017 | -1.0907 | 0.0001 | 0.0004 |
| 350 | X617556 | 1.3565 | 0.0001 | 0.0004 |
| 12 | lactic acid | 0.9544 | 0.0002 | 0.0005 |
| 174 | beta-sitosterol | 1.7498 | 0.0002 | 0.0005 |
| 331 | X367932 | -0.9103 | 0.0002 | 0.0005 |
| 68 | glutamine dehydrated 2TMS minor | -1.5687 | 0.0002 | 0.0006 |
| 121 | elaidic acid | -0.99 | 0.0002 | 0.0006 |
| 189 | 3-hydroxypyridine | 1.4933 | 0.0002 | 0.0007 |
| 117 | mannitol mix spec with histidine | -4.5517 | 0.0003 | 0.0008 |
| 233 | N-acetyl-D-mannosamine major | 1.0495 | 0.0003 | 0.0008 |
| 259 | X211979 | -1.0079 | 0.0003 | 0.0008 |
| 158 | 2-deoxyerythritol | 1.2425 | 0.0003 | 0.0009 |
| 288 | X438057 | 1.5405 | 0.0003 | 0.0009 |
| 101 | alanine 3TMS | -1.1023 | 0.0004 | 0.001 |
| 164 | azelaic acid | -0.6174 | 0.0004 | 0.001 |
| 310 | X508725 | 2.4049 | 0.0004 | 0.001 |
| 22 | ribitol | -0.9238 | 0.0004 | 0.0011 |
| 240 | 2-ketoadipic acid | -1.4055 | 0.0005 | 0.0012 |
| 128 | asparagine dehydrated | -0.786 | 0.0006 | 0.0014 |
| 183 | thymine | 0.9718 | 0.0006 | 0.0015 |
| 343 | X486016 | -22.6493 | 0.0006 | 0.0015 |
| 147 | glycerol-3-galactoside | 1.018 | 0.0006 | 0.0016 |
| 160 | maleimide | -9.4231 | 0.0007 | 0.0016 |
| 338 | X485397 | -18.7638 | 0.0006 | 0.0016 |
| 239 | homovanillic and 4-hydroxymandelic acid - mixed spectrum | 1.1198 | 0.0007 | 0.0018 |
| 286 | X234717 | 1.2037 | 0.0007 | 0.0018 |
| 289 | X223618 | -0.9328 | 0.0007 | 0.0018 |
| 246 | X408731 | -26.3629 | 0.0008 | 0.002 |
| 151 | 1-monoolein | -0.6983 | 0.0009 | 0.0021 |
| 33 | tocopherol alpha | -1.0272 | 0.0009 | 0.0022 |
| 188 | hippuric acid 1TMS | 0.8974 | 0.0009 | 0.0022 |
| 60 | methanolphosphate | 0.9875 | 0.0011 | 0.0025 |
| 325 | X223625 | 0.94 | 0.001 | 0.0025 |
| 30 | cholesterol | 1.0432 | 0.0012 | 0.0028 |
| 79 | glyceric acid | 1.0318 | 0.0012 | 0.0028 |
| 305 | X499123 | -2.8415 | 0.0012 | 0.0028 |
| 11 | alanine | 0.8899 | 0.0012 | 0.0029 |
| 324 | X309540 | -0.7983 | 0.0014 | 0.0031 |
| 81 | N-acetylglutamate | -1.0018 | 0.0014 | 0.0033 |
| 165 | alpha ketoglutaric acid | 1.1013 | 0.0014 | 0.0033 |
| 58 | glutamine dehydrated | -0.9889 | 0.0016 | 0.0036 |
| 154 | 1-monostearin | 1.035 | 0.0016 | 0.0036 |
| 361 | X428311 | -0.7461 | 0.0017 | 0.0037 |
| 36 | ornithine 4TMS | -1.0801 | 0.0018 | 0.0041 |
| 100 | N-methylalanine | 0.881 | 0.002 | 0.0044 |
| 127 | idonic acid NIST | -0.9698 | 0.0022 | 0.0048 |
| 137 | phenylacetic acid | 1.0001 | 0.0022 | 0.0048 |
| 170 | pentadecanoic acid | 0.9119 | 0.0024 | 0.0051 |
| 129 | benzoic acid mix spec | -24.4844 | 0.0025 | 0.0053 |
| 176 | isolinoleic acid NIST | 0.785 | 0.0025 | 0.0053 |
| 220 | 2,3-dihydroxybutanoic acid NIST | 0.8382 | 0.0026 | 0.0054 |
| 90 | taurine | -1.0065 | 0.0028 | 0.0058 |
| 50 | 1,5-anhydroglucitol | -1.1963 | 0.003 | 0.0062 |
| 172 | pyruvic acid | -1.2104 | 0.003 | 0.0062 |
| 224 | lactobionic acid | -0.736 | 0.003 | 0.0062 |
| 277 | X199794 | -1.2782 | 0.0031 | 0.0064 |
| 314 | X465393 | -0.9158 | 0.0031 | 0.0064 |
| 122 | biuret | -0.8438 | 0.0034 | 0.0069 |
| 175 | salicylic acid | -0.5335 | 0.0035 | 0.0072 |
| 48 | caprylic acid | -0.6095 | 0.0038 | 0.0077 |
| 207 | 3-aminoisobutyric acid 1 | 1.0353 | 0.0038 | 0.0077 |
| 215 | saccharic acid | 1.2246 | 0.0038 | 0.0077 |
| 54 | glutamate TMS2x | -0.7228 | 0.0042 | 0.0084 |
| 340 | X537746 | 0.777 | 0.0045 | 0.0089 |
| 257 | X268506 | -0.7792 | 0.0047 | 0.0092 |
| 219 | beta-alanine minor | 0.8917 | 0.0048 | 0.0094 |
| 212 | cysteine-glycine | -0.6372 | 0.0049 | 0.0095 |
| 78 | glycolic acid | -0.9733 | 0.005 | 0.0096 |
| 15 | fructose 2 | 0.6622 | 0.0051 | 0.0098 |
| 96 | 2-deoxytetronic acid NIST | -0.7674 | 0.0056 | 0.0107 |
| 180 | dihydro-3-coumaric acid | -0.6824 | 0.0057 | 0.0107 |
| 10 | glucose 2 | -0.8385 | 0.0059 | 0.0111 |
| 346 | X612627 | 2.8844 | 0.006 | 0.0112 |
| 226 | trehalose | 0.8322 | 0.006 | 0.0113 |
| 88 | shikimic acid | -0.7154 | 0.0064 | 0.012 |
| 141 | inositol allo- | 1.1765 | 0.0065 | 0.0121 |
| 351 | X486054 | 1.1204 | 0.0068 | 0.0125 |
| 293 | X537868 | -2.2832 | 0.0074 | 0.0135 |
| 131 | methionine sulfoxide major | -0.6986 | 0.0078 | 0.0142 |
| 319 | X277432 | -0.7924 | 0.0078 | 0.0142 |
| 201 | N-acetyl-D-hexosamine | 0.6164 | 0.008 | 0.0144 |
| 55 | glucose 1 | 0.6842 | 0.0081 | 0.0145 |
| 163 | threose meox2 | 0.6165 | 0.0082 | 0.0147 |
| 295 | X199942 | 0.8452 | 0.0084 | 0.015 |
| 20 | phenylalanine TMS1x | -0.785 | 0.0089 | 0.0157 |
| 34 | trans-4-hydroxyproline | -0.7006 | 0.0096 | 0.0169 |
| 204 | 5-hydroxymethyl-2-furoic acid NIST | 0.7485 | 0.0098 | 0.0169 |
| 205 | shikimic acid.1 | -0.6924 | 0.0098 | 0.0169 |
| 225 | 3-methoxytyrosine NIST | 0.5893 | 0.0097 | 0.0169 |
| 356 | X566268 | 0.8196 | 0.0097 | 0.0169 |
| 16 | hydroxylamine | -0.7468 | 0.01 | 0.0172 |
| 278 | X339455 | -0.6433 | 0.0111 | 0.019 |
| 192 | 5-hydroxynorvaline NIST | 0.8117 | 0.0113 | 0.0193 |
| 258 | X225446 | 0.7272 | 0.0116 | 0.0197 |
| 23 | glutamine | -0.6535 | 0.0167 | 0.0283 |
| 365 | X225430 | 0.5966 | 0.0173 | 0.0292 |
| 14 | serine | 0.6645 | 0.0183 | 0.0307 |
| 311 | X565868 | 0.6569 | 0.0188 | 0.0313 |
| 248 | X199786 | -0.7067 | 0.0207 | 0.0343 |
| 3 | tryptophan | -0.5861 | 0.0239 | 0.0395 |
| 210 | 4-hydroxyhippuric acid NIST | 0.5893 | 0.027 | 0.0445 |
| 178 | aconitic acid | -0.5305 | 0.0273 | 0.0447 |
| 82 | asparagine minor 2 | -0.781 | 0.0276 | 0.0451 |
| 217 | furoylglycine NIST | 0.625 | 0.0283 | 0.0459 |
| 367 | X270508 | 0.6811 | 0.0294 | 0.0475 |
