# Supplementary material for: Serum and urinary metabolomics and outcomes in cirrhosis
Source: PLoS One. 2019 Sep 27;14(9):e0223061. doi: 10.1371/journal.pone.0223061 (PMC6764675; doi:10.1371/journal.pone.0223061)
Supplement: S10 Table — (DOCX) [file pone.0223061.s019.docx]

| Table S10: Urine 90 day death logistic regression | | | | |
| --- | --- | --- | --- | --- |
| index | label | regression_coefficient | p_value | p_values_adjusted |
| 9 | isothreonic acid | 1.485 | 1.85E-06 | 0 |
| 14 | xylitol | 4.039 | 3E-06 | 0 |
| 18 | indole-3-acetate | 1.7641 | 2.49E-06 | 0 |
| 23 | tyrosine mz147 missing | 1.4871 | 3.83E-06 | 0 |
| 29 | arabinose | 1.6357 | 1.86E-06 | 0 |
| 30 | glucose 1 | 1.0935 | 4.5E-06 | 0 |
| 43 | indole-3-lactate | 2.2115 | 5.25E-07 | 0 |
| 47 | ethanolamine | 1.4766 | 7.92E-07 | 0 |
| 49 | arabitol | 3.2323 | 1.03E-06 | 0 |
| 54 | 4-hydroxyphenylacetic acid | 2.8827 | 1.03E-06 | 0 |
| 62 | isocitric acid | 1.1868 | 7.42E-06 | 0 |
| 67 | fucose 1 + rhamnose 2 | 2.8701 | 1.56E-06 | 0 |
| 68 | cellobiotol | 1.5057 | 5.89E-06 | 0 |
| 69 | urocanic acid | 1.4057 | 6.65E-06 | 0 |
| 70 | ribose | 1.5509 | 3.22E-06 | 0 |
| 72 | 3-aminoisobutyric acid | 1.861 | 0 | 0 |
| 74 | mannitol mix spec with histidine | 1.3815 | 4.91E-06 | 0 |
| 79 | saccharic acid | 1.491 | 2.08E-06 | 0 |
| 80 | phenylalanine | 1.443 | 8.99E-06 | 0 |
| 82 | fucose | 1.5321 | 3.85E-06 | 0 |
| 85 | creatinine | 2.3004 | 4.01E-06 | 0 |
| 89 | hypoxanthine mix spec with ornithine | 1.3657 | 6.69E-06 | 0 |
| 92 | N-acetyl-D-mannosamine 3 | 1.662 | 1.35E-06 | 0 |
| 94 | 3-hydroxy-3-indoleacetic acid | 1.5941 | 4.46E-06 | 0 |
| 96 | xylulose NIST | 1.6698 | 3.82E-06 | 0 |
| 99 | 1,2-anhydro-myo-inositol NIST | 2.1792 | 1.12E-06 | 0 |
| 103 | azelaic acid | 1.5582 | 2.22E-06 | 0 |
| 114 | N-acetyl-D-hexosamine | 1.7458 | 2.19E-06 | 0 |
| 115 | 5'-deoxy-5'-methylthioadenosine | 1.6343 | 1.71E-06 | 0 |
| 118 | 2,3-dihydroxybutanoic acid NIST | 2.0589 | 2.21E-06 | 0 |
| 120 | glucuronic acid mix spec | 2.0898 | 6.90E-08 | 0 |
| 122 | lyxose minor | 1.9538 | 4.38E-07 | 0 |
| 130 | homovanillic and 4-hydroxymandelic acid - mixed spectrum | 2.017 | 7.63E-07 | 0 |
| 131 | glutamic acid | 2.2086 | 1.58E-06 | 0 |
| 134 | isorhamnose | 1.3452 | 0 | 0 |
| 141 | methionine | 2.309 | 1.28E-06 | 0 |
| 142 | 2-deoxyerythritol | 1.2472 | 6.78E-06 | 0 |
| 144 | 5-hydroxy-3-indoleacetic acid | 1.6158 | 4.16E-06 | 0 |
| 147 | mevalonic acid NIST | 2.1795 | 3.4E-06 | 0 |
| 148 | leucine | 1.9233 | 1.86E-06 | 0 |
| 159 | glutamine | 2.308 | 5.43E-06 | 0 |
| 164 | 3-ureidopropionate | 1.9222 | 1.85E-06 | 0 |
| 182 | X326500 | 2.6234 | 2.88E-07 | 0 |
| 185 | X267714 | 1.2898 | 3.22E-06 | 0 |
| 188 | X303152 | 1.9764 | 1.57E-06 | 0 |
| 191 | X645667 | 1.5815 | 2.68E-06 | 0 |
| 192 | X239312 | 1.1966 | 4.96E-06 | 0 |
| 200 | X267760 | 1.3087 | 6.79E-06 | 0 |
| 202 | X200541 | 1.1786 | 0 | 0 |
| 206 | X267723 | 1.1947 | 9.54E-06 | 0 |
| 212 | X288019 | 4.4731 | 2.28E-06 | 0 |
| 216 | X267737 | 2.2818 | 3.82E-07 | 0 |
| 217 | X267670 | 2.0169 | 7.39E-07 | 0 |
| 218 | X267647 | 3.4069 | 4.96E-07 | 0 |
| 221 | X369589 | 1.4257 | 4.56E-06 | 0 |
| 225 | X227675 | 2.7623 | 1.21E-06 | 0 |
| 232 | X303060 | 3.3728 | 1.92E-06 | 0 |
| 235 | X267650 | 1.2867 | 2.22E-06 | 0 |
| 237 | X636875 | 1.855 | 1.38E-06 | 0 |
| 238 | X636805 | 2.1823 | 5.63E-07 | 0 |
| 248 | X636908 | 2.0568 | 2.48E-06 | 0 |
| 250 | X324627 | 1.5909 | 2.53E-06 | 0 |
| 253 | X642793 | 2.2371 | 6.94E-07 | 0 |
| 266 | X228911 | 2.0184 | 9.39E-07 | 0 |
| 274 | X480050 | 2.1158 | 1.64E-06 | 0 |
| 277 | X216860 | 2.5369 | 2.84E-07 | 0 |
| 278 | X199463 | 2.6568 | 1.8E-06 | 0 |
| 280 | X267666 | 1.9622 | 6.34E-06 | 0 |
| 285 | X267701 | 1.4535 | 2.68E-06 | 0 |
| 294 | X303163 | 1.3304 | 7.88E-06 | 0 |
| 295 | X294129 | 1.5787 | 4.36E-06 | 0 |
| 299 | X631981 | 1.3763 | 3.64E-06 | 0 |
| 300 | X629980 | 1.7699 | 1.21E-06 | 0 |
| 318 | X631962 | 2.7739 | 9.46E-06 | 0 |
| 328 | X368056 | 2.8362 | 2.17E-07 | 0 |
| 329 | X636909 | 2.2368 | 3.2E-06 | 0 |
| 332 | X438101 | 4.8435 | 2.54E-06 | 0 |
| 334 | X231792 | 2.8155 | 6.86E-07 | 0 |
| 337 | X636886 | 1.4682 | 4.96E-06 | 0 |
| 351 | X201042 | 2.2326 | 3.22E-07 | 0 |
| 355 | X221571 | 1.576 | 2.88E-06 | 0 |
| 357 | X650967 | 2.0418 | 1.35E-06 | 0 |
| 368 | X213143 | 1.5205 | 5.42E-06 | 0 |
| 371 | X349922 | 2.5369 | 1.05E-06 | 0 |
| 374 | X234622 | 1.312 | 3.71E-06 | 0 |
| 10 | cystine | 1.0056 | 0 | 0.0001 |
| 13 | erythritol | 4.9822 | 0 | 0.0001 |
| 16 | tryptophan | 1.1169 | 0 | 0.0001 |
| 57 | 3,4-dihydroxyphenylacetic acid | 1.1934 | 0 | 0.0001 |
| 60 | butyrolactam NIST | 1.3986 | 0 | 0.0001 |
| 61 | sucrose | 1.5131 | 0 | 0.0001 |
| 86 | glycerol-3-galactoside | 1.2157 | 0 | 0.0001 |
| 93 | 5-aminovaleric acid lactame | 1.9571 | 0 | 0.0001 |
| 98 | 2-hydroxyadipic acid | 1.5092 | 0 | 0.0001 |
| 119 | serine minor | 1.2069 | 0 | 0.0001 |
| 127 | beta-alanine | 1.3638 | 0 | 0.0001 |
| 132 | galactinol major 2 | 1.387 | 0 | 0.0001 |
| 175 | X267653 | 1.0668 | 0 | 0.0001 |
| 178 | X267687 | 1.2294 | 0 | 0.0001 |
| 184 | X267923 | 1.369 | 0 | 0.0001 |
| 190 | X288966 | 1.0637 | 0 | 0.0001 |
| 193 | X636858 | 1.1595 | 0 | 0.0001 |
| 214 | X268106 | 1.1427 | 0 | 0.0001 |
| 224 | X267652 | 3.9887 | 0 | 0.0001 |
| 230 | X640528 | 1.4576 | 0 | 0.0001 |
| 236 | X218821 | 1.2807 | 0 | 0.0001 |
| 239 | X320562 | 1.4622 | 0 | 0.0001 |
| 264 | X289055 | 1.4141 | 0 | 0.0001 |
| 282 | X636846 | 1.4668 | 0 | 0.0001 |
| 306 | X267649 | 1.0459 | 0 | 0.0001 |
| 319 | X381876 | 1.0294 | 0 | 0.0001 |
| 321 | X267765 | 2.19 | 0 | 0.0001 |
| 336 | X644975 | 1.3112 | 0 | 0.0001 |
| 345 | X232659 | 1.3941 | 0 | 0.0001 |
| 347 | X632100 | 1.2509 | 0 | 0.0001 |
| 358 | X644946 | 1.1957 | 0 | 0.0001 |
| 379 | X367950 | 1.066 | 0 | 0.0001 |
| 11 | 4-hydroxyhippuric acid NIST | 1.0781 | 0.0001 | 0.0002 |
| 42 | levoglucosan | 1.131 | 0 | 0.0002 |
| 46 | glycocyamine major | 1.2414 | 0.0001 | 0.0002 |
| 105 | hexuronic acid | 1.0898 | 0.0001 | 0.0002 |
| 116 | pyrogallol | 1.0958 | 0.0001 | 0.0002 |
| 189 | X616746 | 1.1202 | 0 | 0.0002 |
| 240 | X267730 | 1.2131 | 0.0001 | 0.0002 |
| 247 | X267756 | 0.9812 | 0.0001 | 0.0002 |
| 263 | X229199 | 1.5766 | 0.0001 | 0.0002 |
| 276 | X231796 | 1.1115 | 0 | 0.0002 |
| 286 | X267686 | 1.0133 | 0.0001 | 0.0002 |
| 287 | X233005 | 1.2458 | 0.0001 | 0.0002 |
| 310 | X267658 | 1.1667 | 0.0001 | 0.0002 |
| 313 | X637204 | 0.8941 | 0.0001 | 0.0002 |
| 326 | X300451 | 1.0817 | 0.0001 | 0.0002 |
| 335 | X650930 | 1.1457 | 0 | 0.0002 |
| 376 | X485397 | -1.2563 | 0.0001 | 0.0002 |
| 31 | 3-hydroxy-3-methylglutaric acid | 0.9835 | 0.0001 | 0.0003 |
| 51 | gluconic acid | 0.927 | 0.0001 | 0.0003 |
| 129 | inulotriose 1 | 1.2433 | 0.0001 | 0.0003 |
| 227 | X267707 | 0.9095 | 0.0001 | 0.0003 |
| 245 | X636809 | 1.0134 | 0.0001 | 0.0003 |
| 289 | X636954 | 1.036 | 0.0001 | 0.0003 |
| 311 | X236709 | 0.9465 | 0.0001 | 0.0003 |
| 346 | X208647 | 1.4318 | 0.0001 | 0.0003 |
| 26 | xanthine | 1.0232 | 0.0002 | 0.0004 |
| 28 | 1-methyladenosine | 1.0094 | 0.0001 | 0.0004 |
| 170 | arachidic acid | 1.1457 | 0.0001 | 0.0004 |
| 284 | X267715 | 1.053 | 0.0002 | 0.0004 |
| 342 | X328803 | 1.0157 | 0.0002 | 0.0004 |
| 343 | X281409 | 0.9567 | 0.0002 | 0.0004 |
| 344 | X267691 | 1.0334 | 0.0002 | 0.0004 |
| 56 | fructose 1 | 0.931 | 0.0002 | 0.0005 |
| 110 | benzoic acid mix spec | -2.4843 | 0.0002 | 0.0005 |
| 121 | 5-methoxytryptamine | 1.4016 | 0.0002 | 0.0005 |
| 176 | X267675 | 0.9255 | 0.0002 | 0.0005 |
| 195 | X631980 | 0.9169 | 0.0002 | 0.0005 |
| 205 | X408731 | -4.0842 | 0.0002 | 0.0005 |
| 354 | X382318 | 1.2016 | 0.0002 | 0.0005 |
| 2 | threonic acid 2 | 0.9201 | 0.0002 | 0.0006 |
| 91 | citrulline | 1.0084 | 0.0002 | 0.0006 |
| 251 | X267904 | 0.914 | 0.0002 | 0.0006 |
| 41 | lysine | 0.9327 | 0.0003 | 0.0007 |
| 125 | adenosine | 0.9867 | 0.0003 | 0.0007 |
| 137 | UDP-glucuronic acid | 1.0476 | 0.0003 | 0.0007 |
| 181 | X647819 | 0.9662 | 0.0003 | 0.0007 |
| 210 | X231544 | 0.9202 | 0.0003 | 0.0007 |
| 373 | X238549 | 0.9498 | 0.0003 | 0.0007 |
| 34 | N-acetyl-D-mannosamine major | 0.8358 | 0.0004 | 0.0008 |
| 38 | 5-hydroxymethyl-2-furoic acid NIST | 0.9952 | 0.0004 | 0.0008 |
| 66 | histidine | 0.8887 | 0.0004 | 0.0008 |
| 100 | quinolinic acid | 0.8644 | 0.0003 | 0.0008 |
| 113 | phosphoethanolamine | 0.8919 | 0.0004 | 0.0008 |
| 117 | erythronic acid lactone.1 | 1.0039 | 0.0003 | 0.0008 |
| 157 | asparagine | 0.9841 | 0.0004 | 0.0008 |
| 187 | X321685 | 0.8813 | 0.0004 | 0.0008 |
| 367 | X467949 | 0.9376 | 0.0003 | 0.0008 |
| 378 | X438099 | -1.3265 | 0.0004 | 0.0008 |
| 382 | X218829 | 1.1686 | 0.0003 | 0.0008 |
| 261 | X267755 | 0.8735 | 0.0004 | 0.0009 |
| 288 | X228249 | 1.0404 | 0.0004 | 0.0009 |
| 372 | X339455 | 0.8678 | 0.0004 | 0.0009 |
| 380 | X349036 | 0.9684 | 0.0004 | 0.0009 |
| 83 | 6-deoxyglucitol NIST | 1.0778 | 0.0005 | 0.001 |
| 365 | X267937 | 0.8669 | 0.0005 | 0.001 |
| 219 | X225867 | 0.9692 | 0.0005 | 0.0011 |
| 324 | X244467 | 0.9264 | 0.0005 | 0.0011 |
| 171 | lauric acid | 0.89 | 0.0006 | 0.0013 |
| 283 | X267890 | 1.0325 | 0.0006 | 0.0013 |
| 78 | quinic acid | 0.848 | 0.0007 | 0.0014 |
| 81 | N-acetylaspartic acid 1 | 0.831 | 0.0007 | 0.0014 |
| 167 | cholesterol | 0.9036 | 0.0007 | 0.0015 |
| 315 | X304945 | 0.9545 | 0.0008 | 0.0015 |
| 369 | X651283 | 0.736 | 0.0007 | 0.0015 |
| 156 | mannose | 0.9066 | 0.0009 | 0.0018 |
| 6 | valine | 0.7795 | 0.0011 | 0.002 |
| 48 | sorbitol | 0.7659 | 0.001 | 0.002 |
| 203 | X647447 | 0.768 | 0.001 | 0.002 |
| 338 | X485388 | 0.7557 | 0.001 | 0.002 |
| 366 | X636861 | 0.8481 | 0.0011 | 0.002 |
| 37 | cystine minor | 0.8145 | 0.0011 | 0.0021 |
| 108 | 2-deoxyribonic acid | 0.8335 | 0.0011 | 0.0021 |
| 124 | alpha ketoglutaric acid | 0.8323 | 0.0011 | 0.0021 |
| 186 | X203765 | 0.8293 | 0.0011 | 0.0021 |
| 292 | X321716 | 1.0554 | 0.0011 | 0.0021 |
| 198 | X223625 | 0.8533 | 0.0012 | 0.0022 |
| 267 | X205670 | 0.8615 | 0.0012 | 0.0022 |
| 44 | 1-methylinosine NIST | 0.9317 | 0.0012 | 0.0023 |
| 109 | (s)-(+)-mandelic acid | 0.8987 | 0.0013 | 0.0024 |
| 254 | X268093 | 0.8726 | 0.0013 | 0.0024 |
| 136 | galacturonic acid 2 | 0.7909 | 0.0014 | 0.0025 |
| 333 | X241189 | 0.7863 | 0.0014 | 0.0025 |
| 107 | erythronic acid lactone | -0.7002 | 0.0014 | 0.0026 |
| 152 | palatinitol | 1.0815 | 0.0016 | 0.0028 |
| 353 | X480180 | 0.6727 | 0.0016 | 0.0028 |
| 20 | citramalic acid | 0.7721 | 0.0018 | 0.0031 |
| 275 | X294547 | 0.7549 | 0.0017 | 0.0031 |
| 76 | 2-hydroxy-2-methylbutanoic acid | -1.0009 | 0.0018 | 0.0033 |
| 95 | 2-deoxyerythritol NIST | 0.6972 | 0.002 | 0.0035 |
| 204 | X644906 | 0.7259 | 0.002 | 0.0035 |
| 146 | isoleucine | 0.7543 | 0.0022 | 0.0038 |
| 207 | X267926 | 0.7236 | 0.0022 | 0.0038 |
| 233 | X294799 | 0.7445 | 0.0022 | 0.0038 |
| 272 | X241141 | 0.7659 | 0.0022 | 0.0038 |
| 4 | palmitic acid | 0.8063 | 0.0025 | 0.0043 |
| 179 | X269625 | 0.801 | 0.0025 | 0.0043 |
| 104 | propane-1,3-diol NIST | 0.8523 | 0.0028 | 0.0047 |
| 304 | X280553 | 0.7311 | 0.0028 | 0.0047 |
| 158 | proline | 0.9418 | 0.0028 | 0.0048 |
| 269 | X636831 | 0.7455 | 0.0029 | 0.005 |
| 349 | X479886 | 0.6524 | 0.003 | 0.0051 |
| 3 | oxoproline | 0.6814 | 0.0032 | 0.0054 |
| 155 | aspartic acid | 0.8806 | 0.0033 | 0.0054 |
| 348 | X484345 | 0.6657 | 0.0032 | 0.0054 |
| 209 | X234636 | 0.6641 | 0.0035 | 0.0057 |
| 5 | glucose 2 | 0.814 | 0.0036 | 0.0059 |
| 252 | X267782 | 0.7036 | 0.0036 | 0.0059 |
| 138 | inositol allo- | -0.7609 | 0.0038 | 0.0062 |
| 65 | thymine | 0.7194 | 0.004 | 0.0064 |
| 262 | X267751 | 0.7716 | 0.0041 | 0.0066 |
| 19 | ribitol | 0.7883 | 0.0044 | 0.007 |
| 331 | X485464 | 0.6988 | 0.0045 | 0.0072 |
| 123 | asparagine dehydrated | 0.7728 | 0.0047 | 0.0075 |
| 161 | octadecanol | 1.2615 | 0.0047 | 0.0075 |
| 322 | X267696 | 0.7245 | 0.0048 | 0.0075 |
| 356 | X666227 | 0.7009 | 0.0057 | 0.009 |
| 241 | X267692 | 0.5882 | 0.0058 | 0.0091 |
| 293 | X308106 | -0.9354 | 0.0059 | 0.0092 |
| 375 | X644900 | 0.7562 | 0.0059 | 0.0092 |
| 199 | X368028 | 0.6998 | 0.0061 | 0.0094 |
| 255 | X237946 | 0.6734 | 0.0063 | 0.0097 |
| 88 | methylcitrate | 0.6159 | 0.0064 | 0.0098 |
| 211 | X348900 | 0.665 | 0.0067 | 0.0101 |
| 220 | X666613 | 0.6535 | 0.0066 | 0.0101 |
| 377 | X484307 | -0.7479 | 0.0066 | 0.0101 |
| 327 | X267704 | 0.7295 | 0.0068 | 0.0102 |
| 166 | caffeic acid | 0.663 | 0.007 | 0.0105 |
| 97 | adipic acid | 0.7532 | 0.0079 | 0.0117 |
| 150 | valine TMS1x | 0.6624 | 0.0079 | 0.0117 |
| 246 | X267774 | 0.6117 | 0.0079 | 0.0117 |
| 268 | X202095 | 0.6622 | 0.0078 | 0.0117 |
| 1 | alanine | 0.6289 | 0.0081 | 0.0118 |
| 297 | X267994 | 0.6519 | 0.008 | 0.0118 |
| 307 | X232075 | 0.5978 | 0.008 | 0.0118 |
| 281 | X267645 | 0.6666 | 0.0083 | 0.0121 |
| 222 | X268103 | 0.5985 | 0.0085 | 0.0123 |
| 22 | 2-hydroxyglutaric acid | 0.6664 | 0.0087 | 0.0126 |
| 102 | galactonic acid | 0.6071 | 0.0088 | 0.0126 |
| 339 | X483175 | 0.6471 | 0.0111 | 0.0159 |
| 90 | gluconic acid lactone | 0.6203 | 0.0112 | 0.016 |
| 370 | X367997 | 0.803 | 0.0121 | 0.0172 |
| 303 | X310046 | 0.5935 | 0.0122 | 0.0173 |
| 7 | glycolic acid | 0.5456 | 0.0125 | 0.0177 |
| 242 | X267673 | 0.5701 | 0.0128 | 0.018 |
| 59 | conduritol betat epoxide minor | 0.5785 | 0.0133 | 0.0186 |
| 12 | glycine | 0.6117 | 0.0147 | 0.0206 |
| 296 | X268006 | 0.6102 | 0.0154 | 0.0215 |
| 194 | X267816 | 0.6041 | 0.0162 | 0.0225 |
| 229 | X200490 | 0.586 | 0.0169 | 0.0234 |
| 340 | X367993 | 0.7649 | 0.0188 | 0.0259 |
| 140 | talose | -0.5728 | 0.0198 | 0.0272 |
| 183 | X268506 | 0.5338 | 0.0203 | 0.0277 |
| 55 | glutaric acid | 0.5244 | 0.0206 | 0.0281 |
| 21 | glycine TMS1x | -0.6474 | 0.023 | 0.0313 |
| 52 | threitol 2 | 8.7894 | 0.0236 | 0.032 |
| 154 | butane-2,3-diol (NIST) | -0.6379 | 0.026 | 0.0351 |
| 312 | X223535 | -0.5626 | 0.0321 | 0.0431 |
| 40 | malic acid | 0.4946 | 0.0323 | 0.0433 |
| 149 | N-methylalanine | 0.4901 | 0.0336 | 0.0449 |
