# Supplementary material for: Serum and urinary metabolomics and outcomes in cirrhosis
Source: PLoS One. 2019 Sep 27;14(9):e0223061. doi: 10.1371/journal.pone.0223061 (PMC6764675; doi:10.1371/journal.pone.0223061)
Supplement: S11 Table — (DOCX) [file pone.0223061.s020.docx]

| Table S11: Serum logistic regression 90 day transplant | | | | |
| --- | --- | --- | --- | --- |
| index | label | regression_coefficient | p_value | p_values_adjusted |
| 6 | pelargonic acid | -4.2457 | 7.89E-06 | 0 |
| 40 | glutamine 2TMS | -3.3095 | 2.64E-06 | 0 |
| 43 | isoleucine minor | -4.2999 | 6.39E-07 | 0 |
| 47 | capric acid | -2.2893 | 1.7E-06 | 0 |
| 71 | oxalic acid | -1.5792 | 9.94E-06 | 0 |
| 72 | methylhexadecanoic acid | -1.7816 | 9.21E-07 | 0 |
| 73 | fructose 1 | 1.6647 | 1.79E-06 | 0 |
| 84 | arabitol | 2.0682 | 2.39E-06 | 0 |
| 85 | xylitol | 4.1124 | 1.13E-06 | 0 |
| 87 | threitol 2 | 4.1178 | 8.82E-07 | 0 |
| 98 | phenylethylamine | 3.2263 | 9.24E-07 | 0 |
| 104 | maltose 1 | 3.7909 | 2.97E-06 | 0 |
| 110 | glucuronic acid mix spec | 4.4808 | 6.82E-07 | 0 |
| 113 | histidine | -1.7665 | 4.28E-06 | 0 |
| 130 | arachidic acid | -2.6463 | 7.74E-07 | 0 |
| 134 | 4-hydroxyphenylacetic acid | 1.9327 | 1.97E-06 | 0 |
| 144 | 4-hydroxyproline | 1.7066 | 3.32E-06 | 0 |
| 145 | 3-phenyllactic acid | 2.8933 | 2.62E-06 | 0 |
| 146 | nicotinic acid | 8.8331 | 2.43E-06 | 0 |
| 148 | 3,6-anhydrogalactose | 4.1921 | 2.48E-06 | 0 |
| 150 | 5-methoxytryptamine | -1.3251 | 5.84E-07 | 0 |
| 159 | parabanic acid NIST | -1.559 | 9.04E-07 | 0 |
| 179 | 2-oxogluconic acid NIST | 4.0983 | 1.71E-06 | 0 |
| 181 | 1-deoxyerythritol | 2.123 | 2.36E-06 | 0 |
| 185 | galactonic acid | 2.188 | 1.31E-06 | 0 |
| 186 | ribonic acid | 3.0032 | 7.65E-07 | 0 |
| 193 | phthalic acid | 2.4477 | 3.13E-06 | 0 |
| 194 | phosphoric acid.1 | -3.4798 | 7.78E-07 | 0 |
| 196 | asparagine 2TMS minor | 5.8426 | 3.98E-06 | 0 |
| 206 | beta-mannosylglycerate minor | 3.8776 | 6.53E-06 | 0 |
| 216 | erythrose | 2.922 | 1.47E-06 | 0 |
| 223 | N-acetyl-D-tryptophan minor2 | 4.1368 | 4.5E-06 | 0 |
| 234 | homoserine | 2.7539 | 8.27E-07 | 0 |
| 236 | 1-methyladenosine | 1.5623 | 9.47E-06 | 0 |
| 238 | mannose | 4.8981 | 5.02E-06 | 0 |
| 241 | 3,4-dihydroxyphenylacetic acid | 1.716 | 4.16E-06 | 0 |
| 251 | X213253 | -5.3579 | 1.99E-06 | 0 |
| 254 | X356938 | -5.4271 | 8.58E-06 | 0 |
| 255 | X455340 | -2.2822 | 2.05E-06 | 0 |
| 284 | X223548 | -1.5348 | 7.07E-07 | 0 |
| 285 | X201862 | -2.1467 | 8.01E-06 | 0 |
| 290 | X228911 | 3.2109 | 3.91E-06 | 0 |
| 298 | X223505 | 5.6193 | 1.78E-06 | 0 |
| 300 | X597213 | -3.4845 | 7.88E-06 | 0 |
| 315 | X455826 | -1.4185 | 8.28E-06 | 0 |
| 317 | X216428 | 4.282 | 3.38E-06 | 0 |
| 320 | X213193 | 4.6871 | 7.70E-07 | 0 |
| 329 | X223629 | 2.6266 | 4.46E-06 | 0 |
| 335 | X307915 | -1.9862 | 3.27E-06 | 0 |
| 336 | X495239 | -1.9482 | 7.81E-06 | 0 |
| 344 | X227367 | 3.4921 | 2.41E-06 | 0 |
| 347 | X362005 | -4.1827 | 7.20E-07 | 0 |
| 355 | X224849 | 2.8902 | 2.89E-06 | 0 |
| 360 | X208557 | -1.944 | 3.35E-06 | 0 |
| 362 | X216838 | -4.2934 | 1.94E-06 | 0 |
| 363 | X356925 | 3.5734 | 2.91E-06 | 0 |
| 173 | N-acetylglycine NIST | -1.7008 | 9.15E-08 | 6.9E-06 |
| 190 | tartaric acid | -2.2286 | 4.82E-08 | 6.9E-06 |
| 208 | quinolinic acid | 2.3593 | 9.40E-08 | 6.9E-06 |
| 228 | 5-aminovaleric acid lactame | 4.9966 | 8.96E-08 | 6.9E-06 |
| 230 | pantothenic acid | 3.1386 | 7.49E-08 | 6.9E-06 |
| 5 | stearic acid | -2.9132 | 4.20E-07 | 9.3E-06 |
| 17 | threonine minor | -2.4898 | 3.54E-07 | 9.3E-06 |
| 51 | erythritol | 3.9477 | 3.27E-07 | 9.3E-06 |
| 75 | inosine | -2.9196 | 4.49E-07 | 9.3E-06 |
| 92 | phosphoric acid | -6.3592 | 4.36E-07 | 9.3E-06 |
| 109 | 2-hydroxyvaleric acid | -2.0051 | 1.52E-07 | 9.3E-06 |
| 125 | isorhamnose | 2.3024 | 2.41E-07 | 9.3E-06 |
| 187 | 2-ketoisocaproic acid minor | 2.078 | 3.88E-07 | 9.3E-06 |
| 198 | 5-hydroxyindole-3-acetic acid NIST | 2.9462 | 2.43E-07 | 9.3E-06 |
| 301 | X607692 | -2.2075 | 4.56E-07 | 9.3E-06 |
| 318 | X640860 | -1.1141 | 2.49E-07 | 9.3E-06 |
| 326 | X618071 | -3.1896 | 4.32E-07 | 9.3E-06 |
| 359 | X211952 | -2.0865 | 3.99E-07 | 9.3E-06 |
| 74 | methionine | 1.7931 | 0 | 0.0001 |
| 91 | fucose 1 + rhamnose 2 | 1.8253 | 0 | 0.0001 |
| 93 | sucrose | 2.7643 | 0 | 0.0001 |
| 95 | valine TMS1x | -1.9696 | 0 | 0.0001 |
| 107 | pipecolic acid | 5.5231 | 0 | 0.0001 |
| 115 | fucose | 1.9845 | 0 | 0.0001 |
| 120 | tagatose 1 | 1.8452 | 0 | 0.0001 |
| 139 | hypoxanthine mix spec with ornithine | -1.5337 | 0 | 0.0001 |
| 152 | pyrophosphate | 6.4206 | 0 | 0.0001 |
| 156 | glutaric acid | 1.3774 | 0 | 0.0001 |
| 166 | guanosine | -1.1985 | 0 | 0.0001 |
| 168 | propane-1,3-diol NIST | 1.5107 | 0 | 0.0001 |
| 213 | cyclohexylamine NIST | 3.1508 | 0 | 0.0001 |
| 231 | inulobiose 2 | 3.4147 | 0 | 0.0001 |
| 250 | X222169 | 1.2889 | 0 | 0.0001 |
| 262 | X289052 | -1.1334 | 0 | 0.0001 |
| 267 | X200850 | -1.5924 | 0 | 0.0001 |
| 271 | X612625 | 1.224 | 0 | 0.0001 |
| 275 | X223597 | 1.5806 | 0 | 0.0001 |
| 287 | X223566 | -6.427 | 0 | 0.0001 |
| 291 | X381876 | 1.8237 | 0 | 0.0001 |
| 296 | X438101 | 1.7631 | 0 | 0.0001 |
| 297 | X213972 | -7.5585 | 0 | 0.0001 |
| 316 | X314770 | 5.773 | 0 | 0.0001 |
| 328 | X497413 | 1.9351 | 0 | 0.0001 |
| 332 | X484792 | -1.3542 | 0 | 0.0001 |
| 345 | X653345 | 2.2176 | 0 | 0.0001 |
| 357 | X231850 | 1.5224 | 0 | 0.0001 |
| 26 | serine minor | -1.1515 | 0.0001 | 0.0002 |
| 28 | lauric acid | -1.2713 | 0.0001 | 0.0002 |
| 119 | cysteine | -1.4801 | 0.0001 | 0.0002 |
| 245 | 1-methylinosine NIST | 1.1188 | 0.0001 | 0.0002 |
| 259 | X211979 | -1.2731 | 0.0001 | 0.0002 |
| 309 | X199596 | -2.5608 | 0 | 0.0002 |
| 313 | X273773 | 8.508 | 0.0001 | 0.0002 |
| 323 | X223871 | 1.7163 | 0 | 0.0002 |
| 331 | X367932 | -1.0231 | 0 | 0.0002 |
| 24 | proline | 1.7189 | 0.0001 | 0.0003 |
| 149 | adipic acid | -0.9322 | 0.0001 | 0.0003 |
| 161 | 3-aminoisobutyric acid | 1.5414 | 0.0001 | 0.0003 |
| 265 | X207223 | -8.4258 | 0.0001 | 0.0003 |
| 274 | X213143 | 12.3283 | 0.0001 | 0.0003 |
| 305 | X499123 | -2.3738 | 0.0001 | 0.0003 |
| 312 | X301325 | 9.4428 | 0.0001 | 0.0003 |
| 322 | X227352 | 12.7369 | 0.0001 | 0.0003 |
| 101 | alanine 3TMS | -1.3056 | 0.0001 | 0.0004 |
| 171 | dodecane | -1.212 | 0.0001 | 0.0004 |
| 199 | methionine sulfoxide minor1 | 1.609 | 0.0001 | 0.0004 |
| 288 | X438057 | 1.7796 | 0.0001 | 0.0004 |
| 12 | lactic acid | 0.958 | 0.0002 | 0.0005 |
| 45 | lysine | -1.396 | 0.0002 | 0.0005 |
| 174 | beta-sitosterol | 1.8606 | 0.0002 | 0.0005 |
| 68 | glutamine dehydrated 2TMS minor | -1.5229 | 0.0002 | 0.0006 |
| 121 | elaidic acid | -0.9906 | 0.0002 | 0.0006 |
| 203 | cellobiotol | 1.7828 | 0.0002 | 0.0006 |
| 188 | hippuric acid 1TMS | 1.0467 | 0.0003 | 0.0007 |
| 189 | 3-hydroxypyridine | 1.4746 | 0.0002 | 0.0007 |
| 233 | N-acetyl-D-mannosamine major | 1.0372 | 0.0003 | 0.0007 |
| 286 | X234717 | 1.4294 | 0.0003 | 0.0007 |
| 158 | 2-deoxyerythritol | 1.2545 | 0.0003 | 0.0008 |
| 338 | X485397 | -15.1694 | 0.0003 | 0.0008 |
| 350 | X617556 | 1.233 | 0.0003 | 0.0008 |
| 133 | threonic acid 1 | -6.3434 | 0.0004 | 0.0009 |
| 310 | X508725 | 2.3214 | 0.0003 | 0.0009 |
| 337 | X486017 | -0.8969 | 0.0003 | 0.0009 |
| 128 | asparagine dehydrated | -0.8453 | 0.0004 | 0.001 |
| 151 | 1-monoolein | -0.8061 | 0.0004 | 0.001 |
| 183 | thymine | 1.0394 | 0.0004 | 0.001 |
| 48 | caprylic acid | -1.0256 | 0.0005 | 0.0012 |
| 117 | mannitol mix spec with histidine | -3.5746 | 0.0004 | 0.0012 |
| 164 | azelaic acid | -0.6061 | 0.0005 | 0.0012 |
| 129 | benzoic acid mix spec | -14.3425 | 0.0005 | 0.0013 |
| 147 | glycerol-3-galactoside | 1.066 | 0.0005 | 0.0013 |
| 79 | glyceric acid | 1.1645 | 0.0006 | 0.0014 |
| 289 | X223618 | -0.9648 | 0.0006 | 0.0014 |
| 325 | X223625 | 1.0247 | 0.0006 | 0.0014 |
| 343 | X486016 | -22.7259 | 0.0006 | 0.0014 |
| 167 | galactose | 4.3013 | 0.0006 | 0.0015 |
| 239 | homovanillic and 4-hydroxymandelic acid - mixed spectrum | 1.1562 | 0.0006 | 0.0015 |
| 240 | 2-ketoadipic acid | -1.3444 | 0.0007 | 0.0017 |
| 100 | N-methylalanine | 0.99 | 0.0008 | 0.0019 |
| 11 | alanine | 0.9147 | 0.0009 | 0.0021 |
| 22 | ribitol | -0.8446 | 0.0009 | 0.0021 |
| 246 | X408731 | -26.7421 | 0.001 | 0.0022 |
| 30 | cholesterol | 1.1582 | 0.001 | 0.0023 |
| 60 | methanolphosphate | 0.9751 | 0.0011 | 0.0024 |
| 81 | N-acetylglutamate | -1.0544 | 0.0011 | 0.0024 |
| 36 | ornithine 4TMS | -1.1736 | 0.0011 | 0.0025 |
| 361 | X428311 | -0.8125 | 0.0012 | 0.0027 |
| 127 | idonic acid NIST | -1.0688 | 0.0013 | 0.003 |
| 154 | 1-monostearin | 1.0798 | 0.0014 | 0.003 |
| 15 | fructose 2 | 0.7871 | 0.0015 | 0.0033 |
| 33 | tocopherol alpha | -0.9842 | 0.0015 | 0.0033 |
| 50 | 1,5-anhydroglucitol | -1.2949 | 0.0016 | 0.0034 |
| 122 | biuret | -0.9215 | 0.0015 | 0.0034 |
| 324 | X309540 | -0.8102 | 0.0016 | 0.0034 |
| 170 | pentadecanoic acid | 0.9651 | 0.0016 | 0.0035 |
| 165 | alpha ketoglutaric acid | 1.213 | 0.0017 | 0.0037 |
| 172 | pyruvic acid | -1.2528 | 0.0021 | 0.0045 |
| 58 | glutamine dehydrated | -0.9338 | 0.0025 | 0.0053 |
| 34 | trans-4-hydroxyproline | -0.8399 | 0.0027 | 0.0057 |
| 137 | phenylacetic acid | 0.9842 | 0.0028 | 0.0058 |
| 176 | isolinoleic acid NIST | 0.7803 | 0.003 | 0.0061 |
| 319 | X277432 | -0.8976 | 0.0032 | 0.0066 |
| 314 | X465393 | -0.9407 | 0.0033 | 0.0068 |
| 78 | glycolic acid | -1.0196 | 0.0034 | 0.0069 |
| 346 | X612627 | 3.2913 | 0.0035 | 0.0071 |
| 90 | taurine | -0.9836 | 0.0036 | 0.0072 |
| 220 | 2,3-dihydroxybutanoic acid NIST | 0.8124 | 0.0036 | 0.0072 |
| 20 | phenylalanine TMS1x | -0.8967 | 0.0037 | 0.0074 |
| 278 | X339455 | -0.7624 | 0.0038 | 0.0074 |
| 215 | saccharic acid | 1.2383 | 0.004 | 0.0078 |
| 277 | X199794 | -1.1797 | 0.004 | 0.0079 |
| 16 | hydroxylamine | -0.8766 | 0.0041 | 0.008 |
| 295 | X199942 | 1.0104 | 0.0042 | 0.0081 |
| 88 | shikimic acid | -0.7827 | 0.0044 | 0.0085 |
| 175 | salicylic acid | -0.5146 | 0.0044 | 0.0085 |
| 293 | X537868 | -2.4811 | 0.0045 | 0.0086 |
| 54 | glutamate TMS2x | -0.7263 | 0.005 | 0.0096 |
| 163 | threose meox2 | 0.6614 | 0.0051 | 0.0096 |
| 55 | glucose 1 | 0.738 | 0.0052 | 0.0098 |
| 340 | X537746 | 0.7607 | 0.0056 | 0.0105 |
| 226 | trehalose | 0.8381 | 0.0059 | 0.0109 |
| 10 | glucose 2 | -0.7711 | 0.0062 | 0.0112 |
| 180 | dihydro-3-coumaric acid | -0.7057 | 0.0062 | 0.0112 |
| 201 | N-acetyl-D-hexosamine | 0.6586 | 0.0061 | 0.0112 |
| 207 | 3-aminoisobutyric acid 1 | 0.984 | 0.0061 | 0.0112 |
| 224 | lactobionic acid | -0.6272 | 0.0063 | 0.0114 |
| 212 | cysteine-glycine | -0.6264 | 0.0064 | 0.0115 |
| 257 | X268506 | -0.7183 | 0.0068 | 0.0123 |
| 204 | 5-hydroxymethyl-2-furoic acid NIST | 0.8024 | 0.007 | 0.0124 |
| 205 | shikimic acid.1 | -0.7448 | 0.0075 | 0.0133 |
| 219 | beta-alanine minor | 0.8341 | 0.0076 | 0.0135 |
| 311 | X565868 | 0.7724 | 0.0081 | 0.0142 |
| 141 | inositol allo- | 1.1448 | 0.0082 | 0.0144 |
| 351 | X486054 | 1.1142 | 0.0088 | 0.0154 |
| 252 | X309642 | 0.6785 | 0.009 | 0.0157 |
| 248 | X199786 | -0.8107 | 0.0092 | 0.016 |
| 192 | 5-hydroxynorvaline NIST | 0.8233 | 0.01 | 0.0173 |
| 225 | 3-methoxytyrosine NIST | 0.5908 | 0.0103 | 0.0177 |
| 96 | 2-deoxytetronic acid NIST | -0.6617 | 0.0115 | 0.0196 |
| 131 | methionine sulfoxide major | -0.615 | 0.0123 | 0.0208 |
| 356 | X566268 | 0.7835 | 0.0138 | 0.0233 |
| 3 | tryptophan | -0.7176 | 0.0143 | 0.0241 |
| 217 | furoylglycine NIST | 0.6896 | 0.0177 | 0.0296 |
| 258 | X225446 | 0.6976 | 0.0177 | 0.0296 |
| 14 | serine | 0.6738 | 0.0185 | 0.0307 |
| 64 | aspartate minor | -0.6263 | 0.0208 | 0.0344 |
| 178 | aconitic acid | -0.5445 | 0.0218 | 0.0359 |
| 365 | X225430 | 0.5732 | 0.0225 | 0.0369 |
| 126 | galacturonic acid | -0.5877 | 0.025 | 0.0408 |
| 249 | X204344 | -0.6465 | 0.0262 | 0.0425 |
| 38 | succinic acid | -0.5873 | 0.029 | 0.0465 |
| 82 | asparagine minor 2 | -0.7936 | 0.029 | 0.0465 |
| 327 | X486211 | 0.5644 | 0.029 | 0.0465 |
