# Supplementary material for: Serum and urinary metabolomics and outcomes in cirrhosis
Source: PLoS One. 2019 Sep 27;14(9):e0223061. doi: 10.1371/journal.pone.0223061 (PMC6764675; doi:10.1371/journal.pone.0223061)
Supplement: S12 Table — (DOCX) [file pone.0223061.s021.docx]

| Table S12: Urine metabolites 90 day transplant Logistic regression | | | | |
| --- | --- | --- | --- | --- |
| index | label | regression_coefficient | p_value | p_values_adjusted |
| 9 | isothreonic acid | 1.4582 | 2.46E-06 | 0 |
| 14 | xylitol | 4.1883 | 2.68E-06 | 0 |
| 18 | indole-3-acetate | 1.7078 | 3.98E-06 | 0 |
| 23 | tyrosine mz147 missing | 1.4376 | 7.63E-06 | 0 |
| 29 | arabinose | 1.5541 | 2.97E-06 | 0 |
| 30 | glucose 1 | 1.0932 | 5.74E-06 | 0 |
| 43 | indole-3-lactate | 2.1935 | 5.85E-07 | 0 |
| 47 | ethanolamine | 1.4575 | 1.35E-06 | 0 |
| 49 | arabitol | 3.2271 | 1.16E-06 | 0 |
| 54 | 4-hydroxyphenylacetic acid | 2.8521 | 1.1E-06 | 0 |
| 62 | isocitric acid | 1.1378 | 0 | 0 |
| 67 | fucose 1 + rhamnose 2 | 2.8188 | 1.71E-06 | 0 |
| 68 | cellobiotol | 1.4857 | 5.94E-06 | 0 |
| 69 | urocanic acid | 1.399 | 7.25E-06 | 0 |
| 70 | ribose | 1.5392 | 3.59E-06 | 0 |
| 74 | mannitol mix spec with histidine | 1.3355 | 5.81E-06 | 0 |
| 79 | saccharic acid | 1.4689 | 2.05E-06 | 0 |
| 82 | fucose | 1.4751 | 6.11E-06 | 0 |
| 85 | creatinine | 2.1692 | 2.36E-06 | 0 |
| 89 | hypoxanthine mix spec with ornithine | 1.2785 | 9.52E-06 | 0 |
| 92 | N-acetyl-D-mannosamine 3 | 1.6493 | 1.62E-06 | 0 |
| 94 | 3-hydroxy-3-indoleacetic acid | 1.5614 | 4.77E-06 | 0 |
| 96 | xylulose NIST | 1.7503 | 6.47E-06 | 0 |
| 99 | 1,2-anhydro-myo-inositol NIST | 2.1557 | 1.31E-06 | 0 |
| 103 | azelaic acid | 1.5258 | 4.42E-06 | 0 |
| 114 | N-acetyl-D-hexosamine | 1.8391 | 1.83E-06 | 0 |
| 115 | 5'-deoxy-5'-methylthioadenosine | 1.5233 | 2.45E-06 | 0 |
| 118 | 2,3-dihydroxybutanoic acid NIST | 1.9779 | 3.61E-06 | 0 |
| 119 | serine minor | 1.2664 | 6.94E-06 | 0 |
| 120 | glucuronic acid mix spec | 2.1067 | 9.86E-08 | 0 |
| 122 | lyxose minor | 1.9236 | 5.70E-07 | 0 |
| 130 | homovanillic and 4-hydroxymandelic acid - mixed spectrum | 1.9509 | 1.19E-06 | 0 |
| 131 | glutamic acid | 2.1356 | 3.52E-06 | 0 |
| 141 | methionine | 2.2929 | 2.11E-06 | 0 |
| 142 | 2-deoxyerythritol | 1.2303 | 5.77E-06 | 0 |
| 144 | 5-hydroxy-3-indoleacetic acid | 1.582 | 4.48E-06 | 0 |
| 147 | mevalonic acid NIST | 2.1285 | 5.05E-06 | 0 |
| 148 | leucine | 2.1473 | 1.88E-06 | 0 |
| 159 | glutamine | 2.3312 | 4.1E-06 | 0 |
| 164 | 3-ureidopropionate | 1.8328 | 3.25E-06 | 0 |
| 182 | X326500 | 2.5858 | 2.86E-07 | 0 |
| 185 | X267714 | 1.2847 | 3.59E-06 | 0 |
| 188 | X303152 | 1.9523 | 2.14E-06 | 0 |
| 191 | X645667 | 1.5534 | 3.03E-06 | 0 |
| 192 | X239312 | 1.1783 | 8.49E-06 | 0 |
| 200 | X267760 | 1.3623 | 6.01E-06 | 0 |
| 212 | X288019 | 4.5532 | 1.78E-06 | 0 |
| 216 | X267737 | 2.293 | 5.16E-07 | 0 |
| 217 | X267670 | 1.9922 | 1.06E-06 | 0 |
| 218 | X267647 | 3.3584 | 8.56E-07 | 0 |
| 221 | X369589 | 1.4003 | 5.86E-06 | 0 |
| 225 | X227675 | 2.8226 | 9.66E-07 | 0 |
| 232 | X303060 | 3.3537 | 2.84E-06 | 0 |
| 235 | X267650 | 1.2781 | 2.62E-06 | 0 |
| 237 | X636875 | 1.8248 | 1.87E-06 | 0 |
| 238 | X636805 | 2.1598 | 8.34E-07 | 0 |
| 248 | X636908 | 2.0404 | 2.67E-06 | 0 |
| 250 | X324627 | 1.5414 | 4.19E-06 | 0 |
| 253 | X642793 | 2.2171 | 1.1E-06 | 0 |
| 266 | X228911 | 2.0247 | 1.18E-06 | 0 |
| 274 | X480050 | 2.0947 | 1.91E-06 | 0 |
| 277 | X216860 | 2.5268 | 4.49E-07 | 0 |
| 278 | X199463 | 2.6958 | 2.07E-06 | 0 |
| 280 | X267666 | 2.0019 | 4.5E-06 | 0 |
| 285 | X267701 | 1.4205 | 3.16E-06 | 0 |
| 294 | X303163 | 1.3163 | 9.67E-06 | 0 |
| 295 | X294129 | 1.5561 | 4.93E-06 | 0 |
| 299 | X631981 | 1.3731 | 4.76E-06 | 0 |
| 300 | X629980 | 1.7384 | 2.17E-06 | 0 |
| 318 | X631962 | 2.5946 | 0 | 0 |
| 328 | X368056 | 2.8014 | 2.69E-07 | 0 |
| 329 | X636909 | 2.2318 | 3.77E-06 | 0 |
| 332 | X438101 | 4.7374 | 2.69E-06 | 0 |
| 334 | X231792 | 2.7148 | 7.23E-07 | 0 |
| 337 | X636886 | 1.4308 | 6.73E-06 | 0 |
| 351 | X201042 | 2.1162 | 3.14E-07 | 0 |
| 355 | X221571 | 1.5566 | 4.58E-06 | 0 |
| 357 | X650967 | 1.8207 | 2.06E-06 | 0 |
| 368 | X213143 | 1.5022 | 5.61E-06 | 0 |
| 371 | X349922 | 2.5009 | 1.17E-06 | 0 |
| 374 | X234622 | 1.2938 | 4.63E-06 | 0 |
| 10 | cystine | 1.0005 | 0 | 0.0001 |
| 13 | erythritol | 4.9512 | 0 | 0.0001 |
| 60 | butyrolactam NIST | 1.3405 | 0 | 0.0001 |
| 61 | sucrose | 1.5017 | 0 | 0.0001 |
| 72 | 3-aminoisobutyric acid | 1.8308 | 0 | 0.0001 |
| 80 | phenylalanine | 1.3751 | 0 | 0.0001 |
| 86 | glycerol-3-galactoside | 1.1867 | 0 | 0.0001 |
| 93 | 5-aminovaleric acid lactame | 1.8973 | 0 | 0.0001 |
| 98 | 2-hydroxyadipic acid | 1.4758 | 0 | 0.0001 |
| 127 | beta-alanine | 1.3618 | 0 | 0.0001 |
| 132 | galactinol major 2 | 1.3414 | 0 | 0.0001 |
| 134 | isorhamnose | 1.337 | 0 | 0.0001 |
| 175 | X267653 | 1.0675 | 0 | 0.0001 |
| 178 | X267687 | 1.2002 | 0 | 0.0001 |
| 184 | X267923 | 1.3401 | 0 | 0.0001 |
| 190 | X288966 | 1.0271 | 0 | 0.0001 |
| 193 | X636858 | 1.1294 | 0 | 0.0001 |
| 202 | X200541 | 1.1691 | 0 | 0.0001 |
| 206 | X267723 | 1.1656 | 0 | 0.0001 |
| 214 | X268106 | 1.1329 | 0 | 0.0001 |
| 224 | X267652 | 4.0221 | 0 | 0.0001 |
| 230 | X640528 | 1.4387 | 0 | 0.0001 |
| 236 | X218821 | 1.2346 | 0 | 0.0001 |
| 239 | X320562 | 1.4464 | 0 | 0.0001 |
| 264 | X289055 | 1.3812 | 0 | 0.0001 |
| 282 | X636846 | 1.4305 | 0 | 0.0001 |
| 306 | X267649 | 1.0554 | 0 | 0.0001 |
| 319 | X381876 | 1.0541 | 0 | 0.0001 |
| 321 | X267765 | 2.1061 | 0 | 0.0001 |
| 336 | X644975 | 1.3188 | 0 | 0.0001 |
| 345 | X232659 | 1.4008 | 0 | 0.0001 |
| 347 | X632100 | 1.2894 | 0 | 0.0001 |
| 379 | X367950 | 1.0716 | 0 | 0.0001 |
| 16 | tryptophan | 1.0686 | 0.0001 | 0.0002 |
| 57 | 3,4-dihydroxyphenylacetic acid | 1.1064 | 0 | 0.0002 |
| 189 | X616746 | 1.0576 | 0.0001 | 0.0002 |
| 240 | X267730 | 1.1862 | 0.0001 | 0.0002 |
| 247 | X267756 | 0.9696 | 0.0001 | 0.0002 |
| 287 | X233005 | 1.2315 | 0.0001 | 0.0002 |
| 335 | X650930 | 1.1346 | 0.0001 | 0.0002 |
| 358 | X644946 | 1.168 | 0.0001 | 0.0002 |
| 376 | X485397 | -1.3435 | 0.0001 | 0.0002 |
| 11 | 4-hydroxyhippuric acid NIST | 1.0203 | 0.0001 | 0.0003 |
| 42 | levoglucosan | 1.1071 | 0.0001 | 0.0003 |
| 51 | gluconic acid | 0.9175 | 0.0001 | 0.0003 |
| 105 | hexuronic acid | 1.064 | 0.0001 | 0.0003 |
| 116 | pyrogallol | 1.0248 | 0.0001 | 0.0003 |
| 129 | inulotriose 1 | 1.2426 | 0.0001 | 0.0003 |
| 227 | X267707 | 0.9307 | 0.0001 | 0.0003 |
| 245 | X636809 | 1.0072 | 0.0001 | 0.0003 |
| 263 | X229199 | 1.5228 | 0.0001 | 0.0003 |
| 276 | X231796 | 1.0593 | 0.0001 | 0.0003 |
| 286 | X267686 | 0.9984 | 0.0001 | 0.0003 |
| 310 | X267658 | 1.1345 | 0.0001 | 0.0003 |
| 313 | X637204 | 0.8831 | 0.0001 | 0.0003 |
| 326 | X300451 | 1.046 | 0.0001 | 0.0003 |
| 46 | glycocyamine major | 1.1947 | 0.0001 | 0.0004 |
| 110 | benzoic acid mix spec | -2.9617 | 0.0001 | 0.0004 |
| 284 | X267715 | 1.0265 | 0.0002 | 0.0004 |
| 311 | X236709 | 0.9257 | 0.0001 | 0.0004 |
| 343 | X281409 | 0.9744 | 0.0001 | 0.0004 |
| 378 | X438099 | -1.5403 | 0.0001 | 0.0004 |
| 28 | 1-methyladenosine | 0.962 | 0.0002 | 0.0005 |
| 31 | 3-hydroxy-3-methylglutaric acid | 0.9509 | 0.0002 | 0.0005 |
| 289 | X636954 | 1.0026 | 0.0002 | 0.0005 |
| 346 | X208647 | 1.4053 | 0.0002 | 0.0005 |
| 170 | arachidic acid | 1.0892 | 0.0002 | 0.0006 |
| 176 | X267675 | 0.915 | 0.0003 | 0.0006 |
| 195 | X631980 | 0.9083 | 0.0002 | 0.0006 |
| 342 | X328803 | 0.988 | 0.0002 | 0.0006 |
| 26 | xanthine | 0.958 | 0.0003 | 0.0007 |
| 354 | X382318 | 1.1631 | 0.0003 | 0.0007 |
| 344 | X267691 | 0.9722 | 0.0003 | 0.0008 |
| 2 | threonic acid 2 | 0.9076 | 0.0004 | 0.0009 |
| 56 | fructose 1 | 0.9176 | 0.0003 | 0.0009 |
| 91 | citrulline | 0.9784 | 0.0004 | 0.001 |
| 137 | UDP-glucuronic acid | 1.0196 | 0.0004 | 0.001 |
| 251 | X267904 | 0.876 | 0.0004 | 0.001 |
| 125 | adenosine | 0.9751 | 0.0004 | 0.0011 |
| 205 | X408731 | -3.9648 | 0.0004 | 0.0011 |
| 288 | X228249 | 1.0275 | 0.0005 | 0.0011 |
| 373 | X238549 | 0.9188 | 0.0004 | 0.0011 |
| 380 | X349036 | 0.9746 | 0.0005 | 0.0011 |
| 382 | X218829 | 1.1503 | 0.0005 | 0.0011 |
| 107 | erythronic acid lactone | -0.8691 | 0.0005 | 0.0012 |
| 117 | erythronic acid lactone.1 | 0.9779 | 0.0005 | 0.0012 |
| 181 | X647819 | 0.925 | 0.0005 | 0.0012 |
| 34 | N-acetyl-D-mannosamine major | 0.8013 | 0.0006 | 0.0013 |
| 41 | lysine | 0.8827 | 0.0006 | 0.0013 |
| 100 | quinolinic acid | 0.8127 | 0.0006 | 0.0013 |
| 113 | phosphoethanolamine | 0.8739 | 0.0006 | 0.0013 |
| 187 | X321685 | 0.8421 | 0.0006 | 0.0013 |
| 338 | X485388 | 0.8818 | 0.0006 | 0.0013 |
| 365 | X267937 | 0.859 | 0.0006 | 0.0013 |
| 367 | X467949 | 0.865 | 0.0006 | 0.0013 |
| 210 | X231544 | 0.8717 | 0.0007 | 0.0014 |
| 261 | X267755 | 0.8535 | 0.0006 | 0.0014 |
| 121 | 5-methoxytryptamine | 1.2048 | 0.0007 | 0.0015 |
| 324 | X244467 | 0.8977 | 0.0007 | 0.0015 |
| 78 | quinic acid | 0.8494 | 0.0008 | 0.0016 |
| 167 | cholesterol | 0.9117 | 0.0008 | 0.0016 |
| 283 | X267890 | 1.0275 | 0.0008 | 0.0016 |
| 315 | X304945 | 0.9773 | 0.0008 | 0.0016 |
| 38 | 5-hydroxymethyl-2-furoic acid NIST | 0.9475 | 0.0009 | 0.0018 |
| 66 | histidine | 0.8188 | 0.0009 | 0.0018 |
| 76 | 2-hydroxy-2-methylbutanoic acid | -1.1354 | 0.0009 | 0.0018 |
| 157 | asparagine | 0.9267 | 0.0009 | 0.0018 |
| 219 | X225867 | 0.9336 | 0.0009 | 0.0018 |
| 369 | X651283 | 0.7282 | 0.0009 | 0.0018 |
| 83 | 6-deoxyglucitol NIST | 0.9814 | 0.001 | 0.002 |
| 171 | lauric acid | 0.8571 | 0.0011 | 0.0023 |
| 6 | valine | 0.789 | 0.0012 | 0.0024 |
| 372 | X339455 | 0.8145 | 0.0012 | 0.0024 |
| 109 | (s)-(+)-mandelic acid | 0.8834 | 0.0014 | 0.0027 |
| 44 | 1-methylinosine NIST | 0.9132 | 0.0015 | 0.003 |
| 124 | alpha ketoglutaric acid | 0.7977 | 0.0015 | 0.003 |
| 152 | palatinitol | 1.1138 | 0.0015 | 0.003 |
| 48 | sorbitol | 0.7378 | 0.0017 | 0.0032 |
| 81 | N-acetylaspartic acid 1 | 0.7557 | 0.0017 | 0.0033 |
| 186 | X203765 | 0.7732 | 0.0018 | 0.0033 |
| 204 | X644906 | 0.7445 | 0.0018 | 0.0033 |
| 254 | X268093 | 0.8434 | 0.0018 | 0.0033 |
| 275 | X294547 | 0.7438 | 0.0017 | 0.0033 |
| 366 | X636861 | 0.8226 | 0.0017 | 0.0033 |
| 136 | galacturonic acid 2 | 0.7691 | 0.0019 | 0.0034 |
| 156 | mannose | 0.8556 | 0.0018 | 0.0034 |
| 203 | X647447 | 0.7324 | 0.0019 | 0.0035 |
| 37 | cystine minor | 0.7763 | 0.002 | 0.0036 |
| 20 | citramalic acid | 0.7618 | 0.002 | 0.0037 |
| 267 | X205670 | 0.8199 | 0.0021 | 0.0038 |
| 108 | 2-deoxyribonic acid | 0.804 | 0.0022 | 0.0039 |
| 333 | X241189 | 0.7556 | 0.0022 | 0.0039 |
| 146 | isoleucine | 0.7673 | 0.0022 | 0.004 |
| 138 | inositol allo- | -0.8265 | 0.0024 | 0.0043 |
| 198 | X223625 | 0.7672 | 0.0024 | 0.0043 |
| 292 | X321716 | 0.9556 | 0.0025 | 0.0043 |
| 207 | X267926 | 0.7126 | 0.0027 | 0.0046 |
| 353 | X480180 | 0.6386 | 0.0026 | 0.0046 |
| 272 | X241141 | 0.7576 | 0.0028 | 0.0048 |
| 4 | palmitic acid | 0.8051 | 0.0028 | 0.0049 |
