# Supplementary material for: Serum and urinary metabolomics and outcomes in cirrhosis
Source: PLoS One. 2019 Sep 27;14(9):e0223061. doi: 10.1371/journal.pone.0223061 (PMC6764675; doi:10.1371/journal.pone.0223061)
Supplement: S13 Table — (DOCX) [file pone.0223061.s022.docx]

| **Table S13: Serum VIP scores for individual named metabolites for each complication arranged by VIP score** | | | | | | | |
| --- | --- | --- | --- | --- | --- | --- | --- |
| **Death** | **VIP** | **Overt HE** | **VIP** | **Transplant** | **VIP** | **Hospitalization** | **VIP** |
| hypoxanthine mix spec with ornithine | 1.691674911 | sophorose | 1.761664709 | N-acetyl-D-tryptophan minor2 | 2.077881 | 2-deoxyerythritol NIST | 1.743146383 |
| oxoproline | 1.6136051 | **leucine** | 1.632356319 | oxoproline | 1.811711 | conduritol betat epoxide minor | 1.735224901 |
| 2-hydroxybutanoic acid | 1.547067628 | oxoproline | 1.600209198 | caprylic acid | 1.571073 | arabitol | 1.735079543 |
| aconitic acid | 1.532266306 | **isoleucine** | 1.582285313 | xylulose NIST | 1.525188 | 3-phenyllactic acid | 1.582691715 |
| 1-deoxyerythritol | 1.479701386 | stearic acid | 1.543379415 | stearic acid | 1.506294 | **valine** | 1.564061841 |
| urea | 1.467688896 | glycine | 1.533397167 | ornithine | 1.436212 | tocopherol alpha | 1.558857206 |
| heptadecanoic acid NIST | 1.467584884 | **valine** | 1.474016441 | malic acid | 1.428341 | threitol 2 | 1.536478704 |
| palmitic acid | 1.465626513 | icosenoic acid | 1.45732143 | asparagine 2TMS minor | 1.41985 | alpha ketoglutaric acid | 1.507600006 |
| linoleic acid | 1.439986096 | heptadecanoic acid NIST | 1.44794942 | 2-deoxyerythritol NIST | 1.415952 | pseudo uridine | 1.490157196 |
| oleic acid | 1.419174514 | 3-hydroxybutanoic acid mix spec | 1.439422872 | dodecanol | 1.40343 | 2-deoxyerythritol | 1.415289639 |
| 3-hydroxybutanoic acid mix spec | 1.416205224 | palmitic acid | 1.439262932 | trans-4-hydroxyproline | 1.394916 | phenylethylamine | 1.406504891 |
| galacturonic acid 2 | 1.415715043 | linolenic acid | 1.415766801 | tyrosine minor | 1.394313 | ethanolamine | 1.404227462 |
| glucose 2 | 1.409341965 | linoleic acid | 1.414421309 | 2-hydroxybutanoic acid | 1.392622 | **leucine** | 1.40017147 |
| 1-methyladenosine | 1.39527319 | xylitol | 1.401049684 | isolinoleic acid NIST | 1.351142 | 2-ketoadipic acid | 1.378520717 |
| **isoleucine** | 1.388507228 | oleic acid | 1.389973739 | N-acetylglutamate | 1.336039 | N-acetyl-D-hexosamine | 1.376185515 |
| **leucine** | 1.3862066 | 2-hydroxybutanoic acid | 1.386819203 | glycine TMS1x | 1.332613 | inulobiose 2 | 1.351429174 |
| phthalic acid | 1.381706001 | glutamine | 1.381953827 | adipic acid | 1.332586 | maltose 1 | 1.341200867 |
| **valine** | 1.367678245 | 1-deoxyerythritol | 1.356001613 | succinic acid | 1.326415 | **isoleucine** | 1.331328379 |
| 1,5-anhydroglucitol | 1.360892224 | methylhexadecanoic acid | 1.344876304 | threitol 2 | 1.323956 | aspartic acid | 1.330337414 |
| glucuronic acid mix spec | 1.360625469 | tocopherol alpha | 1.338038687 | glutamine | 1.296891 | 3-methoxytyrosine NIST | 1.328860561 |
| linolenic acid | 1.341676552 | isoleucine minor | 1.328081768 | isocitric acid | 1.287417 | malic acid | 1.317041479 |
| stearic acid | 1.338979159 | 2-ketoadipic acid | 1.326955936 | 3-phenyllactic acid | 1.28078 | maleimide | 1.313892994 |
| gamma-tocopherol | 1.331238527 | myristic acid | 1.325946376 | isothreonic acid | 1.277746 | azelaic acid | 1.294718911 |
| 6-deoxyglucitol NIST | 1.328719256 | methanolphosphate | 1.303059774 | aconitic acid | 1.268151 | 3-hydroxypropionic acid | 1.279737264 |
| ornithine 3TMS minor | 1.299020716 | inulobiose 2 | 1.300647943 | beta-alanine minor | 1.249804 | pipecolic acid | 1.270270407 |
| 2-hydroxyvaleric acid | 1.298657414 | asparagine 2TMS minor | 1.298345455 | pantothenic acid | 1.248377 | sucrose | 1.263953137 |
| sucrose | 1.293620715 | dihydro-3-coumaric acid | 1.292105489 | 2-hydroxyglutaric acid | 1.247528 | isothreonic acid | 1.241215908 |
| 2-deoxyerythritol NIST | 1.267463854 | creatinine | 1.276814739 | 3-hydroxybutanoic acid mix spec | 1.227887 | tyrosine mz147 missing | 1.23662399 |
| glycine | 1.249739938 | lysine | 1.275413905 | levoglucosan | 1.224286 | threonic acid 2 | 1.231552785 |
| myristic acid | 1.246375427 | arachidonic acid isomer | 1.273424177 | conduritol betat epoxide minor | 1.210125 | dodecanol | 1.225213251 |
| xylitol | 1.244977955 | asparagine dehydrated | 1.258718225 | glycolic acid | 1.197433 | linolenic acid | 1.21755578 |
| tyrosine mz147 missing | 1.244480355 | galacturonic acid | 1.254719905 | histidine | 1.190907 | xylose 2 | 1.21695225 |
| methylhexadecanoic acid | 1.224357967 | galactose | 1.237673497 | glutamine 2TMS | 1.187242 | fucose 1 + rhamnose 2 | 1.213762072 |
| 3-hydroxypropionic acid | 1.22295149 | aspartic acid | 1.231906273 | 1-methylhydantoin TMS | 1.182288 | 1-monopalmitin | 1.212341172 |
| arachidonic acid isomer | 1.221295828 | cellobiotol | 1.216353521 | fumaric acid | 1.179402 | glycolic acid | 1.206448022 |
| galacturonic acid | 1.219044004 | tyrosine mz147 missing | 1.213907809 | citrulline | 1.16973 | 6-deoxyglucitol NIST | 1.201358031 |
| icosenoic acid | 1.217765789 | arachidic acid | 1.205662673 | cholesterol | 1.163393 | erythritol | 1.200906339 |
| isoleucine minor | 1.217438452 | tyrosine minor | 1.205081544 | inositol allo- | 1.151772 | phenylalanine | 1.198641472 |
| inulobiose 2 | 1.214126297 | hypoxanthine mix spec with ornithine | 1.195927309 | shikimic acid | 1.14936 | 3-hydroxybutanoic acid mix spec | 1.197571117 |
| uridine | 1.212585864 | glutamine 2TMS | 1.189272145 | maltose 1 | 1.143354 | pantothenic acid | 1.193614659 |
| pantothenic acid | 1.204120088 | conduritol betat epoxide minor | 1.187429701 | creatinine | 1.133703 | N-acetylglutamate | 1.189876873 |
| quinolinic acid | 1.203227026 | allantoic acid -H2O | 1.180636124 | N-acetylglycine NIST | 1.132207 | stearic acid | 1.188365732 |
| threitol 2 | 1.191542358 | lauric acid | 1.169693377 | glycine | 1.131313 | cysteine | 1.183736036 |
| asparagine dehydrated | 1.187175449 | galacturonic acid 2 | 1.156295815 | oleic acid | 1.13131 | indole-3-lactate | 1.182565321 |
| ornithine | 1.18589739 | aminomalonic acid | 1.151651235 | palmitic acid | 1.125783 | 5-hydroxynorvaline NIST | 1.181743532 |
| lactobionic acid | 1.18487514 | ornithine 4TMS | 1.150598439 | galactonic acid | 1.114645 | ribonic acid | 1.178891091 |
| maleimide | 1.179184746 | fructose 2 | 1.145586178 | serine minor | 1.10976 | glycine | 1.171481232 |
| sophorose | 1.177309907 | phthalic acid | 1.138120139 | 2,3-dihydroxybutanoic acid NIST | 1.109262 | inositol allo- | 1.169906133 |
| ribonic acid | 1.174608197 | lactobionic acid | 1.138021377 | uracil | 1.10873 | isoleucine minor | 1.164329071 |
| glutamine dehydrated | 1.174570753 | 1-hexadecanol | 1.134557262 | phenylethylamine | 1.106844 | trans-4-hydroxyproline | 1.163040799 |
| ornithine 4TMS | 1.171818449 | cytidine-5'-diphosphate deriv. | 1.13374532 | glycerol | 1.106331 | quinic acid | 1.15966306 |
| maltose 1 | 1.166449681 | threonine | 1.132956374 | alpha ketoglutaric acid | 1.104142 | cellobiotol | 1.159369005 |
| creatinine | 1.149666654 | 6-deoxyglucitol NIST | 1.130906802 | alanine 3TMS | 1.103825 | uracil | 1.157497863 |
| lysine | 1.146583683 | pelargonic acid | 1.130566411 | erythritol | 1.100728 | levoglucosan | 1.152780661 |
| beta-mannosylglycerate minor | 1.140185057 | phenylalanine | 1.120843594 | taurine | 1.097399 | proline | 1.151736115 |
| levoglucosan | 1.133379848 | maleimide | 1.11575176 | arabitol | 1.09594 | cyclohexylamine NIST | 1.14949601 |
| histidine | 1.13165137 | pantothenic acid | 1.115199102 | tocopherol alpha | 1.093592 | cytidine-5'-diphosphate deriv. | 1.148560337 |
| 5-hydroxynorvaline NIST | 1.127573626 | cysteine | 1.108835066 | citric acid | 1.092882 | 2-hydroxybutanoic acid | 1.137666078 |
| 2-deoxytetronic acid NIST | 1.125877926 | succinic acid | 1.108423315 | threonine | 1.087062 | creatinine | 1.137456749 |
| nicotinic acid | 1.124662913 | ornithine 3TMS minor | 1.105638679 | hydroxylamine | 1.086098 | 1-methylhydantoin TMS | 1.131459302 |
| 2-hydroxyglutaric acid | 1.122741689 | histidine | 1.104119037 | 2-deoxyerythritol | 1.078973 | pyruvic acid | 1.128024687 |
| threonine | 1.116306854 | taurine | 1.101923488 | 4-hydroxyproline | 1.064078 | trehalose | 1.12416594 |
| dodecanol | 1.115548433 | 2-oxogluconic acid NIST | 1.098572923 | isorhamnose | 1.054654 | aspartate minor | 1.1221834 |
| citrulline | 1.111090585 | tryptophan | 1.092789243 | cellobiotol | 1.048771 | methionine sulfoxide minor1 | 1.119961611 |
| glucose overload | 1.105060624 | dodecanol | 1.091704471 | aminomalonic acid | 1.048728 | inosine | 1.118580283 |
| elaidic acid | 1.103798338 | asparagine | 1.088859321 | linoleic acid | 1.044842 | glycine TMS1x | 1.114256523 |
| 3-phenyllactic acid | 1.100647647 | 3-aminoisobutyric acid 1 | 1.086340193 | arabinose | 1.044489 | 2-hydroxyglutaric acid | 1.111269798 |
| lauric acid | 1.09987344 | indole-3-lactate | 1.085674777 | furoylglycine NIST | 1.04412 | methionine sulfoxide minor2 | 1.106459814 |
| fructose 2 | 1.097662331 | serine | 1.075455006 | pseudo uridine | 1.043942 | 5-hydroxymethyl-2-furoic acid NIST | 1.101055175 |
| proline | 1.096053018 | elaidic acid | 1.073815524 | methylhexadecanoic acid | 1.041085 | methylhexadecanoic acid | 1.099474972 |
| glycerol | 1.095997318 | ribonic acid | 1.073721305 | indole-3-lactate | 1.041064 | isorhamnose | 1.097082056 |
| cellobiotol | 1.093986513 | benzoic acid mix spec | 1.064128859 | glycocyamine major | 1.037455 | nicotinic acid | 1.091041217 |
| 2-ketoisocaproic acid minor | 1.093225162 | glycerol | 1.055640327 | sucrose | 1.035052 | kynurenine | 1.090118436 |
| palmitoleic acid | 1.092853216 | 1-methyladenosine | 1.055140447 | butyrolactam NIST | 1.033642 | citric acid | 1.089169674 |
| N-acetylglutamate | 1.088834726 | 1-monoolein | 1.054035333 | phenylalanine TMS1x | 1.031399 | glutamic acid | 1.089008899 |
| phenylalanine | 1.088219509 | gamma-tocopherol | 1.052380406 | 3-methoxytyrosine NIST | 1.030261 | asparagine dehydrated | 1.087582682 |
| pyruvic acid | 1.088213719 | 5-methoxytryptamine | 1.051344933 | 4-hydroxyphenylacetic acid | 1.029209 | histidine | 1.080817057 |
| erythritol | 1.084693923 | glycerol-alpha-phosphate | 1.050506267 | 2-oxogluconic acid NIST | 1.025206 | hypoxanthine mix spec with ornithine | 1.080201386 |
| cysteine | 1.074628468 | 3-phenyllactic acid | 1.048831247 | 1-deoxyerythritol | 1.023967 | benzylalcohol | 1.078438933 |
| pentadecanoic acid | 1.070444745 | levoglucosan | 1.046598673 | maleimide | 1.023181 | xylitol | 1.077038821 |
| dihydro-3-coumaric acid | 1.069291291 | threitol 2 | 1.045341611 | cystine | 1.022527 | citrulline | 1.074438068 |
| pipecolic acid | 1.061416641 | sucrose | 1.044803021 | icosenoic acid | 1.02218 | glucuronic acid mix spec | 1.073617732 |
| 3,6-anhydrogalactose | 1.05940161 | beta-mannosylglycerate minor | 1.038943366 | N-acetyl-D-mannosamine major | 1.021473 | phthalic acid | 1.070107859 |
| serine minor | 1.055794371 | 1-methylhydantoin TMS | 1.038836551 | inulobiose 2 | 1.011923 | palmitic acid | 1.064670185 |
| galactonic acid | 1.050188658 | erythritol | 1.03811095 | 1-methyladenosine | 1.010565 | cholesterol | 1.063017718 |
| citramalic acid | 1.034170187 | isolinoleic acid NIST | 1.037175974 | 2-deoxytetronic acid NIST | 1.008123 | tryptophan | 1.062202564 |
| methionine sulfoxide major | 1.033935796 | threonic acid 2 | 1.031939171 | galacturonic acid 2 | 1.001683 | succinic acid | 1.058048825 |
| 3-aminoisobutyric acid | 1.031043674 | homoserine | 1.029136912 | valine TMS1x | 1.000627 | methanolphosphate | 1.057184591 |
| glycerol-alpha-phosphate | 1.030071333 | 5-hydroxynorvaline NIST | 1.02615354 | phenylacetic acid | 0.997505 | arachidonic acid isomer | 1.055560406 |
| aspartic acid | 1.027563022 | urea | 1.023873549 | lactobionic acid | 0.995961 | arabinose | 1.054098032 |
| caprylic acid | 1.026669325 | galactonic acid | 1.021992241 | methanolphosphate | 0.995273 | 3,6-anhydrogalactose | 1.046574956 |
| glutamine dehydrated 2TMS minor | 1.019963677 | hydroxylamine | 1.020906476 | asparagine minor 2 | 0.99191 | lysine | 1.045970891 |
| serine | 1.017675107 | N-methylalanine | 1.010982442 | linolenic acid | 0.990525 | ornithine | 1.043387622 |
| 3-aminoisobutyric acid 1 | 1.014299314 | phosphoethanolamine | 1.009727906 | xylose 2 | 0.990162 | quinolinic acid | 1.037509486 |
| cyclohexylamine NIST | 1.011359289 | 2-ketoisocaproic acid minor | 1.008429203 | ornithine 4TMS | 0.989008 | behenic acid | 1.034763916 |
| threonic acid 1 | 1.008257468 | 2-deoxyerythritol NIST | 1.008215989 | fucose 1 + rhamnose 2 | 0.9888 | taurine | 1.031520837 |
| fucose 1 + rhamnose 2 | 1.006510112 | cystine | 1.002288929 | phenylalanine | 0.987468 | thymine | 1.030730619 |
| benzoic acid mix spec | 1.002515512 | phenylalanine TMS1x | 1.002178064 | threonine minor | 0.986395 | linoleic acid | 1.029781725 |
| trehalose | 0.99895712 | pipecolic acid | 1.001320587 | ribonic acid | 0.983198 | 3-aminoisobutyric acid 1 | 1.025946943 |
| tryptophan | 0.998665092 | mannitol mix spec with histidine | 0.999609105 | tryptophan | 0.982448 | 2-oxogluconic acid NIST | 1.023463088 |
| 4-hydroxyproline | 0.995787599 | maltose 1 | 0.999060586 | tartaric acid | 0.982209 | 2-aminoadipic acid | 1.023045368 |
| 3,4-dihydroxyphenylacetic acid | 0.995361478 | proline | 0.995484666 | shikimic acid.1 | 0.981437 | oleic acid | 1.02145785 |
| valine TMS1x | 0.995245121 | sorbitol | 0.995393244 | hydroxycarbamate NIST | 0.980899 | 2-ketoisocaproic acid minor | 1.017942023 |
| taurine | 0.99494571 | quinolinic acid | 0.994839115 | 3-aminoisobutyric acid | 0.980187 | ornithine 4TMS | 1.016751126 |
| asparagine 2TMS minor | 0.989914193 | oxalic acid | 0.989181898 | myristic acid | 0.979191 | 5-aminovaleric acid lactame | 1.015747812 |
| methionine sulfoxide minor2 | 0.989570676 | hydroxycarbamate NIST | 0.988273502 | vanillic acid | 0.978823 | galacturonic acid | 1.014569882 |
| arabitol | 0.988787003 | 3-hydroxypropionic acid | 0.980906783 | arachidic acid | 0.978071 | serine | 1.013437851 |
| cytidine-5'-diphosphate deriv. | 0.988113907 | aspartate minor | 0.976146757 | methionine sulfoxide major | 0.976211 | alanine | 1.0099951 |
| pseudo uridine | 0.985825908 | glutamic acid | 0.973650915 | 5-hydroxynorvaline NIST | 0.975595 | galactonic acid | 1.006556464 |
| pelargonic acid | 0.98213276 | pyruvic acid | 0.965393642 | glycerol-alpha-phosphate | 0.974259 | threonine | 1.002028542 |
| xylulose NIST | 0.981220135 | erythrose | 0.964603613 | nicotinic acid | 0.973775 | myristic acid | 0.996771527 |
| 2,3-dihydroxybutanoic acid NIST | 0.976208233 | fructose 1 | 0.961429539 | glucose overload | 0.971812 | isocitric acid | 0.995631297 |
| 2-oxogluconic acid NIST | 0.972816715 | threonic acid 1 | 0.957929823 | 3,6-anhydrogalactose | 0.966816 | xylulose NIST | 0.994053719 |
| tyrosine minor | 0.969951199 | kynurenine | 0.957630085 | methionine | 0.966343 | 2-deoxytetronic acid NIST | 0.993569427 |
| arabinose | 0.968228487 | xanthine | 0.954062387 | galactose | 0.96608 | glycerol-3-galactoside | 0.990145288 |
| xylose 2 | 0.968001199 | 3-aminoisobutyric acid | 0.953006932 | pyruvic acid | 0.964161 | fructose 1 | 0.986570009 |
| methionine sulfoxide minor1 | 0.965994567 | alanine | 0.951956238 | gluconic acid | 0.959759 | 1-methylinosine NIST | 0.98629368 |
| aminomalonic acid | 0.965753667 | adipic acid | 0.949319449 | N-methylalanine | 0.958916 | fucose | 0.985515766 |
| glutamine 2TMS | 0.962894318 | serine minor | 0.948665764 | fucose | 0.958206 | gamma-tocopherol | 0.980996427 |
| indole-3-lactate | 0.960239043 | 3,6-anhydrogalactose | 0.948498526 | indole-3-acetate | 0.956627 | cystine | 0.980942767 |
| 2-ketoadipic acid | 0.957759691 | glucuronic acid mix spec | 0.940161009 | 3-hydroxypyridine | 0.955891 | homoserine | 0.979523504 |
| tocopherol alpha | 0.956583028 | asparagine minor 2 | 0.938749984 | ornithine 3TMS minor | 0.952668 | fumaric acid | 0.972615793 |
| fucose | 0.953215135 | capric acid | 0.938248522 | cyclohexylamine NIST | 0.951007 | glycocyamine major | 0.972367697 |
| glutamine | 0.951539099 | N-acetyl-D-tryptophan minor2 | 0.937785194 | lysine | 0.945925 | caprylic acid | 0.971922628 |
| hydroxylamine | 0.951058412 | palmitoleic acid | 0.937640029 | asparagine | 0.941941 | homovanillic and 4-hydroxymandelic acid - mixed spectrum | 0.969356754 |
| butyrolactam NIST | 0.944773753 | threonine minor | 0.935446607 | phosphoethanolamine | 0.940123 | lactobionic acid | 0.962646911 |
| capric acid | 0.943587611 | glucose 2 | 0.929799173 | proline | 0.939782 | lauric acid | 0.95521014 |
| erythrose | 0.933516825 | arabitol | 0.929091635 | glutamic acid | 0.939482 | benzoic acid mix spec | 0.954796046 |
| isothreonic acid | 0.932648995 | valine TMS1x | 0.925736479 | hydrocinnamic acid | 0.936628 | N-acetyl-D-mannosamine major | 0.954411209 |
| xanthine | 0.932100184 | cholesterol | 0.917644901 | cystine minor | 0.933709 | glycerol-alpha-phosphate | 0.954003923 |
| glutamate TMS2x | 0.93046322 | nicotinic acid | 0.917370823 | homovanillic and 4-hydroxymandelic acid - mixed spectrum | 0.933365 | glycerol | 0.953245149 |
| glycolic acid | 0.92868149 | butyrolactam NIST | 0.917258904 | uric acid (mix spec with myo-inositol) | 0.932884 | N-acetylglycine NIST | 0.939036879 |
| cholesterol | 0.926277815 | citramalic acid | 0.916922811 | leucine | 0.92966 | phenylalanine TMS1x | 0.938800624 |
| methanolphosphate | 0.923495798 | fucose 1 + rhamnose 2 | 0.912979465 | tyrosine mz147 missing | 0.928843 | 4-hydroxyproline | 0.936529027 |
| biuret | 0.923343611 | biuret | 0.90942676 | quinolinic acid | 0.928275 | isolinoleic acid NIST | 0.934411903 |
| fructose 1 | 0.920561109 | methionine | 0.909118303 | elaidic acid | 0.927368 | shikimic acid.1 | 0.931429028 |
| 5-methoxytryptamine | 0.914984114 | N-acetylglutamate | 0.906554168 | gamma-tocopherol | 0.922061 | asparagine | 0.928774953 |
| sorbitol | 0.912361956 | citrulline | 0.90567703 | tagatose 1 | 0.917434 | tyrosine minor | 0.923947898 |
| homoserine | 0.910012325 | glycine TMS1x | 0.898321125 | serine | 0.916771 | shikimic acid | 0.922827714 |
| kynurenine | 0.907664027 | methionine sulfoxide minor2 | 0.895651772 | ethanolamine | 0.911033 | 3-hydroxypyridine | 0.916278306 |
| succinic acid | 0.905062601 | glutamine dehydrated 2TMS minor | 0.895107236 | homoserine | 0.907266 | glutamine | 0.915450687 |
| threonine minor | 0.899898274 | pseudo uridine | 0.89292459 | dihydro-3-coumaric acid | 0.907101 | gluconic acid | 0.912739408 |
| glutamic acid | 0.897210665 | 4-hydroxyproline | 0.888886425 | 2-ketoisocaproic acid minor | 0.902807 | glutamine 2TMS | 0.909715918 |
| phosphoric acid.1 | 0.896868358 | 1-monopalmitin | 0.886366708 | pipecolic acid | 0.901502 | saccharic acid | 0.909276079 |
| ribitol | 0.896608964 | lactic acid | 0.885702811 | isoleucine | 0.895079 | N-methylalanine | 0.908924488 |
| asparagine | 0.896559291 | glycolic acid | 0.884817896 | methionine sulfoxide minor2 | 0.893649 | aminomalonic acid | 0.906734896 |
| quinic acid | 0.895867561 | tagatose 1 | 0.884468262 | sorbitol | 0.893525 | paracetamol | 0.903358016 |
| mannitol mix spec with histidine | 0.892697237 | shikimic acid | 0.884146842 | glucuronic acid mix spec | 0.884584 | biuret | 0.90233218 |
| N-methylalanine | 0.882233676 | fucose | 0.883554142 | methionine sulfoxide minor1 | 0.883143 | cystine minor | 0.900168513 |
| shikimic acid | 0.880834985 | alanine 3TMS | 0.882909874 | pelargonic acid | 0.883131 | cysteine-glycine | 0.900014962 |
| cystine | 0.877836291 | idonic acid NIST | 0.88270322 | 1-methylinosine NIST | 0.880289 | threonic acid 1 | 0.895276104 |
| galactose | 0.87662212 | 1,5-anhydroglucitol | 0.880521222 | arachidonic acid isomer | 0.877168 | hydroxylamine | 0.894291903 |
| behenic acid | 0.874017938 | cysteine-glycine | 0.879903634 | biuret | 0.876048 | methionine sulfoxide major | 0.89121242 |
| indole-3-acetate | 0.871923857 | beta-alanine | 0.877955672 | cysteine | 0.875768 | butyrolactam NIST | 0.891056685 |
| glutaric acid | 0.8706093 | cyclohexylamine NIST | 0.874571928 | valine | 0.871968 | asparagine minor 2 | 0.888490391 |
| isorhamnose | 0.866838261 | caprylic acid | 0.873165715 | citramalic acid | 0.870158 | icosenoic acid | 0.883292688 |
| glyceric acid | 0.866671927 | pentadecanoic acid | 0.873040016 | saccharic acid | 0.869863 | glutamine dehydrated | 0.883017501 |
| beta-alanine | 0.864502353 | gluconic acid | 0.872664414 | benzoic acid mix spec | 0.869604 | phosphoethanolamine | 0.879145508 |
| idonic acid NIST | 0.863254914 | 2-hydroxyvaleric acid | 0.872218184 | cysteine-glycine | 0.869199 | valine TMS1x | 0.876814012 |
| lactic acid | 0.859823316 | 2,3-dihydroxybutanoic acid NIST | 0.866503106 | thymine | 0.862234 | 1-methyladenosine | 0.875694688 |
| isolinoleic acid NIST | 0.852549976 | hippuric acid 1TMS | 0.866075832 | glutamine dehydrated 2TMS minor | 0.858562 | pelargonic acid | 0.875560865 |
| uracil | 0.850760393 | phosphoric acid.1 | 0.865905233 | idonic acid NIST | 0.855242 | phosphoric acid | 0.875199245 |
| mannose | 0.850425592 | 3-hydroxypyridine | 0.860377061 | heptadecanoic acid NIST | 0.854795 | glucose 2 | 0.871778577 |
| hydroxycarbamate NIST | 0.850133795 | methionine sulfoxide minor1 | 0.84383235 | benzylalcohol | 0.852543 | pentadecanoic acid | 0.867792967 |
| 1-methylhydantoin TMS | 0.842518592 | shikimic acid.1 | 0.843208746 | aspartate minor | 0.849323 | 3-aminoisobutyric acid | 0.862126226 |
| 3-methoxytyrosine NIST | 0.842199308 | glutamate TMS2x | 0.836316505 | lauric acid | 0.848801 | N-acetyl-D-tryptophan minor2 | 0.861899425 |
| naproxen | 0.840820487 | mannose | 0.830664563 | 3,4-dihydroxyphenylacetic acid | 0.848435 | guanosine | 0.861516834 |
| phenylalanine TMS1x | 0.834939174 | parabanic acid NIST | 0.829301968 | 2-ketoadipic acid | 0.848306 | 1-hexadecanol | 0.860131346 |
| alanine | 0.833078598 | glyceric acid | 0.821165837 | 5-hydroxymethyl-2-furoic acid NIST | 0.844292 | serine minor | 0.857795756 |
| methionine | 0.831729022 | isothreonic acid | 0.818367394 | cytidine-5'-diphosphate deriv. | 0.842858 | octadecanol | 0.849786284 |
| fumaric acid | 0.828942389 | 3-methoxytyrosine NIST | 0.817483361 | 1-monopalmitin | 0.841765 | erythrose | 0.849167897 |
| shikimic acid.1 | 0.825882209 | ornithine | 0.811706842 | aspartic acid | 0.840669 | propane-1,3-diol NIST | 0.847242717 |
| N-acetyl-D-mannosamine major | 0.820012731 | arabinose | 0.80769185 | 2-deoxyribonic acid | 0.837473 | galactose | 0.843766032 |
| cysteine-glycine | 0.816809724 | glucose 1 | 0.804217446 | sophorose | 0.832686 | methionine | 0.843679063 |
| tagatose 1 | 0.812912425 | methionine sulfoxide major | 0.803313722 | erythrose | 0.832167 | 4-hydroxyphenylacetic acid | 0.839421969 |
| trans-4-hydroxyproline | 0.811618229 | uracil | 0.796893673 | phosphoric acid.1 | 0.83104 | indole-3-acetate | 0.839372669 |
| phosphoethanolamine | 0.811592005 | 2-hydroxyglutaric acid | 0.795875327 | mannose | 0.825329 | hydrocinnamic acid | 0.828974658 |
| alanine 3TMS | 0.808098643 | uridine | 0.791255814 | hippuric acid 1TMS | 0.825124 | glutamine dehydrated 2TMS minor | 0.826127087 |
| 1-monopalmitin | 0.803337805 | behenic acid | 0.790051212 | glutamine dehydrated | 0.820653 | glutamate TMS2x | 0.824330493 |
| ethanolamine | 0.802819332 | octadecanol | 0.786325233 | 3-hydroxypropionic acid | 0.816857 | 2,3-dihydroxybutanoic acid NIST | 0.821693602 |
| 2-deoxyribonic acid | 0.802297376 | pyrophosphate | 0.784416914 | xylitol | 0.816292 | alanine 3TMS | 0.82147007 |
| glycine TMS1x | 0.795491384 | dodecane | 0.784337466 | threonic acid 1 | 0.815462 | dihydro-3-coumaric acid | 0.818042915 |
| hippuric acid 1TMS | 0.787500487 | 1-monostearin | 0.771646796 | beta-mannosylglycerate minor | 0.815362 | glucose overload | 0.817682532 |
| dodecane | 0.785601876 | tartaric acid | 0.766073059 | glutaric acid | 0.809944 | sorbitol | 0.815909368 |
| arachidic acid | 0.78173422 | 3,4-dihydroxyphenylacetic acid | 0.764017395 | mannitol mix spec with histidine | 0.809779 | galacturonic acid 2 | 0.811045808 |
| glucose 1 | 0.780596932 | trehalose | 0.763904848 | glyceric acid | 0.809559 | capric acid | 0.805021841 |
| 1-monostearin | 0.773100636 | 2-deoxyerythritol | 0.760449004 | octadecanol | 0.808943 | lactic acid | 0.800797209 |
| uric acid (mix spec with myo-inositol) | 0.767481037 | N-acetyl-D-mannosamine 3 | 0.760048161 | allantoic acid -H2O | 0.803292 | 2-deoxyribonic acid | 0.800029395 |
| 5-hydroxymethyl-2-furoic acid NIST | 0.766259164 | 2-deoxyribonic acid | 0.758185386 | parabanic acid NIST | 0.802873 | ornithine 3TMS minor | 0.798157029 |
| beta-alanine minor | 0.76206851 | inositol allo- | 0.75761571 | pyrophosphate | 0.801307 | elaidic acid | 0.797746955 |
| N-acetylglycine NIST | 0.760802947 | xylose 2 | 0.754952368 | asparagine dehydrated | 0.797835 | mannose | 0.796999432 |
| aspartate minor | 0.755068326 | trans-4-hydroxyproline | 0.740831796 | 5-hydroxyindole-3-acetic acid NIST | 0.794005 | dodecane | 0.795703785 |
| pyrophosphate | 0.753267035 | beta-sitosterol | 0.73826209 | palmitoleic acid | 0.789781 | asparagine 2TMS minor | 0.794887705 |
| asparagine minor 2 | 0.745959275 | benzylalcohol | 0.736059072 | beta-sitosterol | 0.789659 | oxalic acid | 0.793441659 |
| conduritol betat epoxide minor | 0.745883039 | 4-hydroxyhippuric acid NIST | 0.729319839 | lactic acid | 0.786577 | phosphoric acid.1 | 0.791923998 |
| 3-hydroxypyridine | 0.736272337 | glucose overload | 0.72911381 | oxalic acid | 0.782087 | oxoproline | 0.79010916 |
| 2-deoxyerythritol | 0.735669899 | 5-hydroxymethyl-2-furoic acid NIST | 0.720337847 | trehalose | 0.780813 | glyceric acid | 0.789662874 |
| phenylethylamine | 0.734636609 | 1-methylinosine NIST | 0.718142609 | glucose 2 | 0.779424 | glutaric acid | 0.788259637 |
| furoylglycine NIST | 0.728447486 | phenylethylamine | 0.717853134 | 3-aminoisobutyric acid 1 | 0.778934 | hippuric acid 1TMS | 0.7854199 |
| parabanic acid NIST | 0.725615344 | furoylglycine NIST | 0.71641008 | 1-monostearin | 0.775108 | idonic acid NIST | 0.774864694 |
| citric acid | 0.724667416 | alpha ketoglutaric acid | 0.708002542 | isoleucine minor | 0.773102 | threonine minor | 0.773921605 |
| N-acetyl-D-hexosamine | 0.721079379 | indole-3-acetate | 0.705040928 | 2-aminoadipic acid | 0.773039 | heptadecanoic acid NIST | 0.769288704 |
| alpha ketoglutaric acid | 0.71896124 | fumaric acid | 0.703497614 | 1-monoolein | 0.764092 | hydroxycarbamate NIST | 0.769183324 |
| homovanillic and 4-hydroxymandelic acid - mixed spectrum | 0.716829974 | 2-deoxytetronic acid NIST | 0.698107253 | 5-aminovaleric acid lactame | 0.757862 | furoylglycine NIST | 0.768412279 |
| glycocyamine major | 0.714230429 | glycerol-3-galactoside | 0.69143758 | pentadecanoic acid | 0.753251 | beta-alanine | 0.768244102 |
| N-acetyl-D-mannosamine 3 | 0.702335794 | xylulose NIST | 0.690763566 | alanine | 0.748129 | citramalic acid | 0.768092761 |
| propane-1,3-diol NIST | 0.700901981 | threose meox2 | 0.683472242 | threonic acid 2 | 0.741863 | mannitol mix spec with histidine | 0.764267688 |
| gluconic acid | 0.700699004 | cholic acid | 0.6807068 | fructose 1 | 0.741821 | allantoic acid -H2O | 0.76132695 |
| adipic acid | 0.69992987 | propane-1,3-diol NIST | 0.673959542 | propane-1,3-diol NIST | 0.726157 | phenylacetic acid | 0.760155613 |
| inosine | 0.697017186 | 5-hydroxyindole-3-acetic acid NIST | 0.670724983 | N-acetyl-D-hexosamine | 0.719871 | beta-mannosylglycerate minor | 0.754155893 |
| glycerol-3-galactoside | 0.696209188 | cystine minor | 0.668115408 | galacturonic acid | 0.717389 | 1-monostearin | 0.746443723 |
| threose meox2 | 0.694423054 | quinic acid | 0.667567516 | behenic acid | 0.713105 | palmitoleic acid | 0.743175785 |
| cystine minor | 0.684497782 | citric acid | 0.652425612 | phthalic acid | 0.711426 | fructose 2 | 0.739146552 |
| beta-sitosterol | 0.683575009 | glycocyamine major | 0.651146158 | glucose 1 | 0.707899 | beta-alanine minor | 0.737739856 |
| octadecanol | 0.68227883 | 5-aminovaleric acid lactame | 0.649910355 | dodecane | 0.705849 | N-acetyl-D-mannosamine 3 | 0.737334579 |
| oxalic acid | 0.680717923 | beta-alanine minor | 0.646777835 | 2-hydroxyvaleric acid | 0.705825 | pyrophosphate | 0.727546009 |
| 5-aminovaleric acid lactame | 0.678919614 | thymine | 0.637395488 | phosphoric acid | 0.705428 | ribitol | 0.726403246 |
| 1-methylinosine NIST | 0.672820691 | glutamine dehydrated | 0.634029339 | capric acid | 0.701645 | arachidic acid | 0.723492706 |
| malic acid | 0.672064856 | N-acetyl-D-mannosamine major | 0.626076123 | 6-deoxyglucitol NIST | 0.693832 | glucose 1 | 0.718854348 |
| inositol allo- | 0.666693139 | N-acetylglycine NIST | 0.6224768 | kynurenine | 0.69352 | 1-monoolein | 0.713977505 |
| benzylalcohol | 0.654819517 | vanillic acid | 0.617220265 | beta-alanine | 0.683914 | 1-deoxyerythritol | 0.704512775 |
| 5-hydroxyindole-3-acetic acid NIST | 0.653340806 | malic acid | 0.611594047 | urea | 0.681525 | aconitic acid | 0.696890394 |
| N-acetyl-D-tryptophan minor2 | 0.651459302 | isorhamnose | 0.611164362 | 2-hydroxyhippuric acid | 0.676176 | xanthine | 0.694198403 |
| 4-hydroxyphenylacetic acid | 0.648754696 | homovanillic and 4-hydroxymandelic acid - mixed spectrum | 0.605332283 | uridine | 0.671223 | naproxen | 0.685501214 |
| isocitric acid | 0.637220606 | isocitric acid | 0.583595376 | glutamate TMS2x | 0.665327 | 2-hydroxyhippuric acid | 0.680585837 |
| 1-monoolein | 0.626095512 | ribose | 0.578554791 | 5-methoxytryptamine | 0.664959 | 2-hydroxyvaleric acid | 0.676403102 |
| salicylic acid | 0.618419168 | 4-hydroxyphenylacetic acid | 0.570027097 | N-acetyl-D-mannosamine 3 | 0.656673 | 5-methoxytryptamine | 0.658300528 |
| cholic acid | 0.61770909 | phosphoric acid | 0.569008574 | fructose 2 | 0.634134 | parabanic acid NIST | 0.645255308 |
| phenylacetic acid | 0.61172227 | ethanolamine | 0.567343708 | ribose | 0.631317 | 3,4-dihydroxyphenylacetic acid | 0.63800701 |
| 2-hydroxyhippuric acid | 0.606383985 | paracetamol | 0.5656711 | 1-hexadecanol | 0.620484 | adipic acid | 0.636565348 |
| 1-hexadecanol | 0.587650856 | saccharic acid | 0.561987939 | azelaic acid | 0.612436 | uridine | 0.636250998 |
| vanillic acid | 0.575948437 | 2-aminoadipic acid | 0.560060266 | glycerol-3-galactoside | 0.598885 | ribose | 0.627636435 |
| saccharic acid | 0.566625712 | ribitol | 0.559946352 | xanthine | 0.568268 | vanillic acid | 0.617147281 |
| hydrocinnamic acid | 0.562026724 | inosine | 0.557809606 | threose meox2 | 0.568003 | 5-hydroxyindole-3-acetic acid NIST | 0.613781454 |
| allantoic acid -H2O | 0.560808315 | 2-hydroxyhippuric acid | 0.538842134 | guanosine | 0.566528 | urea | 0.570995974 |
| thymine | 0.556365698 | naproxen | 0.526799737 | paracetamol | 0.559725 | cholic acid | 0.560662065 |
| 2-aminoadipic acid | 0.551460153 | aconitic acid | 0.520756533 | hypoxanthine mix spec with ornithine | 0.545535 | tagatose 1 | 0.554747917 |
| 4-hydroxyhippuric acid NIST | 0.517244157 | N-acetyl-D-hexosamine | 0.511891595 | 1,5-anhydroglucitol | 0.534859 | uric acid (mix spec with myo-inositol) | 0.553580595 |
| ribose | 0.507206899 | phenylacetic acid | 0.504171246 | inosine | 0.519224 | threose meox2 | 0.540317644 |
| paracetamol | 0.484312227 | glutaric acid | 0.473230726 | 4-hydroxyhippuric acid NIST | 0.479671 | beta-sitosterol | 0.525445855 |
| phosphoric acid | 0.465009633 | hydrocinnamic acid | 0.422296132 | quinic acid | 0.438397 | tartaric acid | 0.503818819 |
| guanosine | 0.461225303 | salicylic acid | 0.360834718 | ribitol | 0.420485 | sophorose | 0.452928151 |
| tartaric acid | 0.385457049 | guanosine | 0.331771238 | cholic acid | 0.407479 | 1,5-anhydroglucitol | 0.445692697 |
| threonic acid 2 | 0.347659521 | azelaic acid | 0.324974151 | salicylic acid | 0.390784 | salicylic acid | 0.391368701 |
| azelaic acid | 0.235309123 | uric acid (mix spec with myo-inositol) | 0.308361941 | naproxen | 0.384533 | 4-hydroxyhippuric acid NIST | 0.362632818 |
