# Supplementary material for: Serum and urinary metabolomics and outcomes in cirrhosis
Source: PLoS One. 2019 Sep 27;14(9):e0223061. doi: 10.1371/journal.pone.0223061 (PMC6764675; doi:10.1371/journal.pone.0223061)
Supplement: S14 Table — (DOCX) [file pone.0223061.s023.docx]

| **Table S14: Urine VIP scores for individual named metabolites for each complication arranged by VIP score** | | | | | | | |
| --- | --- | --- | --- | --- | --- | --- | --- |
| **Death** | **VIP** | **Overt HE** | **VIP** | **Transplant** | **VIP** | **Hospitalizations** | **VIP** |
| 3-aminoisobutyric acid | 1.643070115 | xylitol | 1.872164501 | phosphoric acid.1 | 1.725698799 | glycolic acid | 1.827212618 |
| 2,3-dihydroxybutanoic acid NIST | 1.614889928 | 5-methoxytryptamine | 1.867140102 | 2-hydroxyvaleric acid | 1.701237906 | 3-hydroxypropionic acid | 1.7560861 |
| 2-hydroxy-2-methylbutanoic acid | 1.610992622 | citric acid | 1.805605851 | 2-deoxytetronic acid NIST | 1.692077564 | 2-hydroxyvaleric acid | 1.584754621 |
| 3-ureidopropionate | 1.605150109 | creatinine | 1.741549209 | butane-2,3-diol (NIST) | 1.650237473 | butane-2,3-diol (NIST) | 1.573148827 |
| creatinine | 1.596144716 | 2-hydroxy-2-methylbutanoic acid | 1.738767214 | serine minor | 1.630710133 | creatinine | 1.55118776 |
| 2-hydroxyvaleric acid | 1.54878599 | 2-hydroxyvaleric acid | 1.52753461 | leucine | 1.625056096 | 2,3-dihydroxybutanoic acid NIST | 1.545142682 |
| 5-methoxytryptamine | 1.528630586 | glutamine | 1.505993268 | adipic acid | 1.624222805 | fructose 2 | 1.542568903 |
| uracil | 1.482652188 | glycolic acid | 1.45153304 | creatinine | 1.61593923 | glycine | 1.530902016 |
| glycine TMS1x | 1.478569024 | dodecane | 1.414469964 | oxalic acid | 1.560206429 | glycine TMS1x | 1.491975324 |
| proline | 1.451424687 | azelaic acid | 1.414292736 | 2-hydroxy-2-methylbutanoic acid | 1.498640399 | azelaic acid | 1.411987043 |
| glycolic acid | 1.443983922 | histidine | 1.403092474 | glycine TMS1x | 1.46031286 | fructose 1 | 1.409805348 |
| xylitol | 1.399886017 | threitol 2 | 1.40223294 | indole-3-lactate | 1.460261311 | 2-hydroxy-2-methylbutanoic acid | 1.397753125 |
| 3-hydroxypropionic acid | 1.397233101 | UDP-glucuronic acid | 1.398986905 | 3-hydroxybutanoic acid mix spec | 1.443439445 | xylulose NIST | 1.391645123 |
| butane-2,3-diol (NIST) | 1.391963441 | N-methylalanine | 1.386943204 | isorhamnose | 1.420245818 | benzoic acid mix spec | 1.38144168 |
| pelargonic acid | 1.370941662 | glycine TMS1x | 1.382226302 | benzoic acid mix spec | 1.408113448 | cyclohexylamine NIST | 1.342356615 |
| benzoic acid mix spec | 1.352670887 | 2,3-dihydroxybutanoic acid NIST | 1.380952853 | 3-ureidopropionate | 1.407285453 | threitol 2 | 1.32700058 |
| 2-deoxyerythritol NIST | 1.342240486 | butane-2,3-diol (NIST) | 1.367090966 | galacturonic acid | 1.386165728 | dodecane | 1.304573934 |
| citric acid | 1.340997281 | shikimic acid | 1.349692331 | glycolic acid | 1.381902268 | uracil | 1.285124236 |
| shikimic acid | 1.332763509 | inulotriose 1 | 1.34653941 | dodecane | 1.315481375 | stearic acid | 1.281632065 |
| fructose 1 | 1.322880587 | benzoic acid mix spec | 1.343034592 | proline | 1.309401844 | histidine | 1.257383399 |
| 5-hydroxymethyl-2-furoic acid NIST | 1.318307827 | oleic acid | 1.329231889 | palmitic acid | 1.293881775 | glutamic acid | 1.244119679 |
| glycine | 1.316429198 | pelargonic acid | 1.324870728 | cyclohexylamine NIST | 1.291276952 | alanine | 1.21756936 |
| threitol 2 | 1.300298179 | 5-hydroxymethyl-2-furoic acid NIST | 1.322340278 | 2-hydroxyhippuric acid | 1.282603474 | lysine | 1.215900452 |
| inulotriose 1 | 1.298822839 | methionine | 1.313365419 | threitol 2 | 1.258926856 | butyrolactam NIST | 1.204686675 |
| 3-hydroxypyridine | 1.294602045 | 3-hydroxypropionic acid | 1.294859591 | valine | 1.254448658 | xylitol | 1.195380495 |
| ethanolamine | 1.289697637 | glycine | 1.291035868 | cholesterol | 1.250806762 | 1-methylinosine NIST | 1.19303807 |
| succinic acid | 1.275114141 | inositol allo- | 1.287595078 | fructose 1 | 1.242569425 | oleic acid | 1.192078904 |
| histidine | 1.268622049 | glutamic acid | 1.280902653 | methionine | 1.227120347 | phosphoric acid.1 | 1.191737034 |
| N-methylalanine | 1.253537739 | levoglucosan | 1.273998362 | N-methylalanine | 1.216667203 | inositol allo- | 1.178333157 |
| glucose 2 | 1.247157093 | proline | 1.251723264 | glycerol-3-galactoside | 1.213303976 | cysteine | 1.177505388 |
| serine 4TMS | 1.240844133 | palatinitol | 1.251498758 | citric acid | 1.190171662 | cholesterol | 1.175242222 |
| lyxose minor | 1.239851832 | serine | 1.243573886 | ethanolamine | 1.189983298 | glyceric acid | 1.149741049 |
| glutamic acid | 1.218545894 | glycerol | 1.236278381 | adenosine | 1.188483621 | aspartic acid | 1.145356442 |
| xylulose NIST | 1.218174205 | 2-deoxyerythritol NIST | 1.225663238 | 5-methoxytryptamine | 1.17668657 | leucine | 1.139100024 |
| dodecane | 1.217034051 | phosphoric acid.1 | 1.215215378 | xylulose NIST | 1.175654095 | valine | 1.135457006 |
| oxalic acid | 1.198505734 | succinic acid | 1.20070614 | 3-aminoisobutyric acid | 1.174255063 | methionine | 1.132005562 |
| glyceric acid | 1.195775329 | beta-alanine | 1.191819991 | isoleucine | 1.165970631 | palmitic acid | 1.129830499 |
| 2-deoxytetronic acid NIST | 1.194197927 | 3-aminoisobutyric acid | 1.187503694 | hypoxanthine mix spec with ornithine | 1.14888831 | 5-aminovaleric acid lactame | 1.128551607 |
| valine | 1.19287686 | cellobiotol | 1.181113194 | 2-deoxyerythritol | 1.137947782 | proline | 1.126178749 |
| palatinitol | 1.190519098 | arabinose | 1.180362761 | dehydroascorbic acid | 1.134110153 | indole-3-lactate | 1.125345781 |
| xanthine | 1.177359116 | lysine | 1.174012938 | glycerol | 1.121219354 | N-methylalanine | 1.124667884 |
| isoleucine | 1.17163608 | valine | 1.157215153 | 3-hydroxypropionic acid | 1.118028651 | N-acetyl-D-hexosamine | 1.122320511 |
| serine | 1.167340997 | caffeic acid | 1.151698057 | glycine | 1.109505358 | ethanolamine | 1.118669529 |
| levoglucosan | 1.156764777 | tryptophan | 1.150911709 | sucrose | 1.108546208 | beta-alanine | 1.103084497 |
| methionine | 1.149596902 | 4-hydroxyhippuric acid NIST | 1.146091316 | thymine | 1.099282672 | 3-hydroxypyridine | 1.095181936 |
| hypoxanthine mix spec with ornithine | 1.146693162 | ethanolamine | 1.134730567 | xanthine | 1.09099352 | erythritol | 1.093149036 |
| dehydroascorbic acid | 1.138156634 | leucine | 1.131020228 | UDP-glucuronic acid | 1.077512296 | 4-hydroxyhippuric acid NIST | 1.083035801 |
| tryptophan | 1.131796162 | xylulose NIST | 1.122493664 | glutamic acid | 1.074221804 | galacturonic acid | 1.071247664 |
| ribitol | 1.120727753 | indole-3-acetate | 1.115189561 | beta-alanine | 1.071864298 | isoleucine | 1.064136563 |
| tyrosine mz147 missing | 1.119557874 | stearic acid | 1.109352419 | quinic acid | 1.066691033 | 3-aminoisobutyric acid | 1.063105297 |
| furoylglycine NIST | 1.103361958 | erythritol | 1.101666057 | glutamine | 1.059350163 | mannose | 1.055396249 |
| erythritol | 1.097933954 | galacturonic acid | 1.087889908 | uracil | 1.057247609 | glucose 1 | 1.055018038 |
| propane-1,3-diol NIST | 1.084653582 | xanthine | 1.074537146 | furoylglycine NIST | 1.051523403 | pelargonic acid | 1.054984446 |
| oleic acid | 1.079930508 | 2-deoxytetronic acid NIST | 1.071836993 | fructose 2 | 1.050483308 | quinic acid | 1.054195605 |
| valine TMS1x | 1.077036683 | 5-aminovaleric acid lactame | 1.06381631 | 5'-deoxy-5'-methylthioadenosine | 1.042779822 | serine | 1.050204121 |
| oxoproline | 1.07307549 | mevalonic acid NIST | 1.059789151 | inulotriose 1 | 1.042727173 | lyxose minor | 1.041804011 |
| erythronic acid lactone.1 | 1.072961761 | propane-1,3-diol NIST | 1.058793102 | oxoproline | 1.039820082 | oxoproline | 1.033504757 |
| N-acetylaspartic acid 1 | 1.072494667 | lyxose minor | 1.05168362 | phosphoric acid | 1.034044087 | hypoxanthine mix spec with ornithine | 1.03316724 |
| leucine | 1.071119863 | adenosine | 1.042422404 | oleic acid | 1.02936118 | 5-hydroxymethyl-2-furoic acid NIST | 1.030615966 |
| inositol allo- | 1.061155542 | phosphoric acid | 1.041934467 | (s)-(+)-mandelic acid | 1.026369482 | arabinose | 1.019547957 |
| tartaric acid | 1.060937638 | fucose 1 + rhamnose 2 | 1.026283046 | valine TMS1x | 1.023552269 | capric acid | 1.015395375 |
| mannose | 1.04118438 | thymine | 1.017548858 | xylitol | 1.011762437 | 3-hydroxy-3-methylglutaric acid | 1.015290115 |
| cystine | 1.034497751 | cystine | 1.017393042 | 2,3-dihydroxybutanoic acid NIST | 1.00715157 | gluconic acid lactone | 1.009114431 |
| azelaic acid | 1.029719696 | butyrolactam NIST | 1.017163334 | stearic acid | 1.006388146 | glycerol | 1.006711163 |
| butyrolactam NIST | 1.023495116 | cysteine | 1.015323676 | erythritol | 1.004318202 | 3-ureidopropionate | 0.993330189 |
| cyclohexylamine NIST | 1.020852673 | uracil | 1.011488157 | serine | 1.003938426 | hippuric acid 1TMS | 0.9900487 |
| thymine | 1.018812005 | gluconic acid | 1.008860495 | threonine | 0.997408731 | cystine | 0.986583539 |
| 1-methylinosine NIST | 1.013596073 | hypoxanthine mix spec with ornithine | 1.003419199 | arabitol | 0.989521262 | glycocyamine major | 0.98522364 |
| palmitic acid | 0.997624499 | fructose 1 | 1.002451211 | lysine | 0.980866189 | phenylalanine | 0.982779061 |
| pyrogallol | 0.989960279 | arabitol | 0.997153262 | gluconic acid | 0.979923951 | 5-methoxytryptamine | 0.978297801 |
| talose | 0.989850426 | cyclohexylamine NIST | 0.995172453 | cysteine | 0.9764577 | tryptophan | 0.962982707 |
| lysine | 0.982782116 | phenylalanine | 0.989943272 | 3-hydroxypyridine | 0.967833049 | 4-hydroxyphenylacetic acid | 0.962837081 |
| adenosine | 0.979205089 | 1-methylinosine NIST | 0.989067829 | 5-hydroxymethyl-2-furoic acid NIST | 0.965468224 | thymine | 0.960496036 |
| alanine | 0.979136443 | 3-hydroxypyridine | 0.98818222 | phenylalanine | 0.964270186 | glutamine | 0.958332395 |
| N-acetyl-D-hexosamine | 0.970898638 | talose | 0.982128642 | glycocyamine major | 0.960257124 | citrulline | 0.950142851 |
| glycocyamine major | 0.968109265 | fructose 2 | 0.97759969 | levoglucosan | 0.95592037 | citric acid | 0.948599584 |
| stearic acid | 0.966962006 | 1,2-anhydro-myo-inositol NIST | 0.967784808 | methylcitrate | 0.951779442 | valine TMS1x | 0.946986153 |
| beta-alanine | 0.965150073 | palmitic acid | 0.961514255 | hexuronic acid | 0.94860956 | succinic acid | 0.946004673 |
| (s)-(+)-mandelic acid | 0.964509537 | fucose | 0.946479659 | 2-hydroxyadipic acid | 0.94687932 | 2-hydroxyhippuric acid | 0.944907576 |
| hydroxylamine | 0.960317484 | 2-deoxyribonic acid | 0.942105042 | galacturonic acid 2 | 0.944447492 | arachidic acid | 0.939498597 |
| galacturonic acid | 0.957081386 | valine TMS1x | 0.939820375 | butyrolactam NIST | 0.943779946 | 1,2-anhydro-myo-inositol NIST | 0.938300266 |
| 1,2-anhydro-myo-inositol NIST | 0.952399489 | serine 4TMS | 0.939321281 | 4-hydroxyhippuric acid NIST | 0.942597505 | glucose 2 | 0.936020891 |
| phenylalanine | 0.946791476 | citrulline | 0.935455967 | N-acetyl-D-hexosamine | 0.928738264 | propane-1,3-diol NIST | 0.934359402 |
| fructose 2 | 0.945175086 | methylcitrate | 0.928750034 | erythronic acid lactone | 0.923558418 | quinolinic acid | 0.928052697 |
| mevalonic acid NIST | 0.944959287 | glycocyamine major | 0.926408631 | palatinitol | 0.920372286 | methylhexadecanoic acid | 0.92514156 |
| arabinose | 0.941128491 | 4-hydroxyphenylacetic acid | 0.917755432 | alpha ketoglutaric acid | 0.913143384 | talose | 0.924898991 |
| phosphoethanolamine | 0.940391617 | dehydroascorbic acid | 0.916880643 | N-acetylaspartic acid 1 | 0.908564891 | serine 4TMS | 0.924032004 |
| alanine 3TMS | 0.938291336 | furoylglycine NIST | 0.904046911 | malic acid | 0.905633698 | shikimic acid | 0.921123082 |
| serine minor | 0.932969583 | ascorbic acid | 0.887344542 | fumaric acid | 0.904990355 | 3,4-dihydroxyphenylacetic acid | 0.918122289 |
| UDP-glucuronic acid | 0.932311813 | citramalic acid | 0.886205877 | salicylic acid | 0.895585008 | 2-deoxytetronic acid NIST | 0.917801943 |
| citrulline | 0.925847432 | aspartic acid | 0.880510135 | isocitric acid | 0.894075839 | 2-deoxyerythritol NIST | 0.916697867 |
| glutamine | 0.925464284 | isoleucine | 0.878605034 | saccharic acid | 0.892084043 | furoylglycine NIST | 0.911639788 |
| fucose 1 + rhamnose 2 | 0.924215318 | asparagine dehydrated | 0.870818997 | cystine | 0.891279502 | 1-methyladenosine | 0.900821531 |
| arabitol | 0.924098087 | alanine | 0.870544583 | glyceric acid | 0.880658665 | lactic acid | 0.898636677 |
| erythronic acid lactone | 0.916840927 | erythronic acid lactone | 0.869462101 | tartaric acid | 0.879372382 | erythronic acid lactone.1 | 0.898333375 |
| glutaric acid | 0.903540043 | quinolinic acid | 0.864446972 | 1-methylinosine NIST | 0.873383812 | (s)-(+)-mandelic acid | 0.896029309 |
| saccharic acid | 0.902039624 | asparagine | 0.860107812 | glutaric acid | 0.871520968 | pyrogallol | 0.895163123 |
| indole-3-acetate | 0.90160663 | isorhamnose | 0.858222631 | sorbitol | 0.86835102 | fucose 1 + rhamnose 2 | 0.893457899 |
| glycerol | 0.899738308 | sorbitol | 0.856016031 | myristic acid | 0.866866394 | arabitol | 0.893227021 |
| 2-hydroxyglutaric acid | 0.891519992 | glucose 2 | 0.855622626 | idonic acid NIST | 0.866468807 | alpha ketoglutaric acid | 0.892096526 |
| homovanillic and 4-hydroxymandelic acid - mixed spectrum | 0.883042203 | sucrose | 0.850413804 | 2-hydroxyglutaric acid | 0.8639808 | asparagine | 0.874870622 |
| cholesterol | 0.878917715 | parabanic acid NIST | 0.84332296 | talose | 0.861013039 | glucuronic acid mix spec | 0.868159744 |
| sucrose | 0.873626735 | malic acid | 0.842478842 | tryptophan | 0.858916878 | threonine | 0.862962579 |
| isocitric acid | 0.868123292 | mannose | 0.83475507 | succinic acid | 0.855140767 | methylcitrate | 0.861772182 |
| 4-hydroxyphenylacetic acid | 0.866268288 | tyrosine mz147 missing | 0.831869852 | uric acid (mix spec with myo-inositol) | 0.848360312 | isorhamnose | 0.859443105 |
| aspartic acid | 0.865275405 | ribitol | 0.830996809 | inositol allo- | 0.847236161 | lauric acid | 0.857888363 |
| arachidic acid | 0.865232816 | N-acetyl-D-mannosamine major | 0.829681825 | 3-hydroxy-3-indoleacetic acid | 0.843454776 | sorbitol | 0.855397953 |
| alpha ketoglutaric acid | 0.860505129 | N-acetylaspartic acid 1 | 0.828931616 | indole-3-acetate | 0.843141551 | ribitol | 0.846151391 |
| phosphoric acid.1 | 0.85729038 | threonine | 0.828240226 | aspartic acid | 0.841470424 | xanthine | 0.8459588 |
| asparagine dehydrated | 0.856245602 | cholesterol | 0.823215722 | hippuric acid 1TMS | 0.841453495 | 2-hydroxyadipic acid | 0.845386532 |
| lauric acid | 0.855902561 | pyrogallol | 0.816731896 | quinolinic acid | 0.837869181 | urocanic acid | 0.842081681 |
| 2-deoxyribonic acid | 0.855583415 | homovanillic and 4-hydroxymandelic acid - mixed spectrum | 0.815001721 | fucose 1 + rhamnose 2 | 0.834180464 | adenosine | 0.841573606 |
| N-acetyl-D-mannosamine major | 0.852830124 | myristic acid | 0.813398733 | methylhexadecanoic acid | 0.833688824 | palatinitol | 0.840728821 |
| 3,4-dihydroxyphenylacetic acid | 0.848892325 | oxoproline | 0.805379329 | propane-1,3-diol NIST | 0.831006833 | mevalonic acid NIST | 0.838723871 |
| asparagine | 0.841233289 | 3,4-dihydroxyphenylacetic acid | 0.800777442 | 5-hydroxy-3-indoleacetic acid | 0.826573015 | inulotriose 1 | 0.838017009 |
| pyrophosphate | 0.839403445 | capric acid | 0.796561239 | histidine | 0.809500542 | serine minor | 0.834588954 |
| sorbitol | 0.83617335 | methylhexadecanoic acid | 0.79536533 | 3,4-dihydroxyphenylacetic acid | 0.799341984 | fumaric acid | 0.827833915 |
| 2-deoxyerythritol | 0.83479594 | alanine 3TMS | 0.790515451 | citramalic acid | 0.79740062 | isothreonic acid | 0.826913566 |
| 2-hydroxyadipic acid | 0.827848815 | glutaric acid | 0.780415046 | pelargonic acid | 0.790762663 | gluconic acid | 0.824587352 |
| cysteine | 0.827287509 | galacturonic acid 2 | 0.777558292 | 4-hydroxyphenylacetic acid | 0.790509966 | caffeic acid | 0.822007876 |
| fumaric acid | 0.826365424 | galactinol major 2 | 0.753573494 | N-acetyl-D-mannosamine major | 0.781713596 | N-acetyl-D-mannosamine 3 | 0.819454984 |
| N-acetyl-D-mannosamine 3 | 0.819793995 | adipic acid | 0.750653844 | pyrogallol | 0.780159457 | ascorbic acid | 0.817218369 |
| methylcitrate | 0.814954012 | urocanic acid | 0.748789053 | shikimic acid | 0.778914637 | threonic acid 2 | 0.817090574 |
| quinolinic acid | 0.813822162 | indole-3-lactate | 0.745253399 | glycerol-alpha-phosphate | 0.775601683 | 2-deoxyribonic acid | 0.805198583 |
| urea | 0.808756736 | hydroxylamine | 0.743122401 | octadecanol | 0.766025689 | N-acetylaspartic acid 1 | 0.797486589 |
| cellobiotol | 0.799206098 | lauric acid | 0.741975969 | ribose | 0.764020165 | galacturonic acid 2 | 0.797215332 |
| malic acid | 0.798378086 | phosphoethanolamine | 0.737741383 | hydroxylamine | 0.762848156 | alanine 3TMS | 0.793456036 |
| 3-hydroxy-3-methylglutaric acid | 0.792648831 | arachidic acid | 0.731186887 | threonic acid 2 | 0.756679051 | 5'-deoxy-5'-methylthioadenosine | 0.786441247 |
| indole-3-lactate | 0.790728967 | N-acetyl-D-mannosamine 3 | 0.724652263 | gluconic acid lactone | 0.752134214 | myristic acid | 0.785118945 |
| threonine | 0.790280084 | fumaric acid | 0.72306434 | lactic acid | 0.750348496 | hydroxylamine | 0.784916708 |
| myristic acid | 0.789942501 | oxalic acid | 0.719779613 | lyxose minor | 0.749487007 | pyrophosphate | 0.783625486 |
| 4-hydroxyhippuric acid NIST | 0.789133785 | lactic acid | 0.713502835 | asparagine dehydrated | 0.744056236 | tyrosine mz147 missing | 0.778646641 |
| galacturonic acid 2 | 0.787268299 | 3-hydroxybutanoic acid mix spec | 0.713377584 | glucuronic acid mix spec | 0.742982387 | hexuronic acid | 0.777438992 |
| glucose 1 | 0.783233124 | alpha ketoglutaric acid | 0.71166758 | citrulline | 0.742785553 | isocitric acid | 0.776982254 |
| methylhexadecanoic acid | 0.776539602 | tartaric acid | 0.707919922 | phosphoethanolamine | 0.740700801 | homovanillic and 4-hydroxymandelic acid - mixed spectrum | 0.769615938 |
| capric acid | 0.772950883 | ribose | 0.705680212 | cellobiotol | 0.736798792 | asparagine dehydrated | 0.766579655 |
| fucose | 0.764366309 | pyrophosphate | 0.703079217 | capric acid | 0.734792709 | 3-hydroxy-3-indoleacetic acid | 0.766515321 |
| conduritol betat epoxide minor | 0.762191672 | glyceric acid | 0.698279889 | alanine | 0.732282117 | oxalic acid | 0.764466964 |
| parabanic acid NIST | 0.760459752 | 2-hydroxyadipic acid | 0.697265156 | glucose 1 | 0.730208211 | glutaric acid | 0.764235036 |
| uric acid (mix spec with myo-inositol) | 0.759939313 | saccharic acid | 0.69422893 | ribitol | 0.728091989 | 5-hydroxy-3-indoleacetic acid | 0.760237518 |
| ribose | 0.756416243 | 3-ureidopropionate | 0.692368223 | glucose 2 | 0.723919497 | sucrose | 0.751654996 |
| isothreonic acid | 0.738649579 | hexuronic acid | 0.689680862 | tyrosine mz147 missing | 0.71982111 | citramalic acid | 0.751089734 |
| phosphoric acid | 0.733427201 | N-acetyl-D-hexosamine | 0.685382088 | 2-deoxyribonic acid | 0.71313523 | urea | 0.749738835 |
| gluconic acid lactone | 0.730156881 | 3-hydroxy-3-methylglutaric acid | 0.664762539 | 3-hydroxy-3-methylglutaric acid | 0.69692553 | parabanic acid NIST | 0.74028788 |
| mannitol mix spec with histidine | 0.725501216 | glycerol-alpha-phosphate | 0.662512973 | serine 4TMS | 0.696295599 | phosphoethanolamine | 0.739995336 |
| caffeic acid | 0.725429575 | idonic acid NIST | 0.6616138 | 1,2-anhydro-myo-inositol NIST | 0.694333106 | glycerol-alpha-phosphate | 0.738952563 |
| idonic acid NIST | 0.723572343 | serine minor | 0.659458222 | lauric acid | 0.693448428 | 2-deoxyerythritol | 0.737277296 |
| glycerol-alpha-phosphate | 0.717144365 | glucuronic acid mix spec | 0.658682525 | parabanic acid NIST | 0.692419684 | galactinol major 2 | 0.73578706 |
| threonic acid 2 | 0.709078341 | uric acid (mix spec with myo-inositol) | 0.64932701 | asparagine | 0.689099663 | UDP-glucuronic acid | 0.733571207 |
| gluconic acid | 0.707718741 | glucose 1 | 0.642010631 | azelaic acid | 0.671710735 | erythronic acid lactone | 0.731850223 |
| isorhamnose | 0.707563837 | hippuric acid 1TMS | 0.628404471 | cystine minor | 0.662442327 | malic acid | 0.726055125 |
| octadecanol | 0.702836385 | isocitric acid | 0.627594317 | ascorbic acid | 0.659901913 | ribose | 0.716907722 |
| glucuronic acid mix spec | 0.701344065 | mannitol mix spec with histidine | 0.614675173 | 5-aminovaleric acid lactame | 0.656511048 | saccharic acid | 0.714913304 |
| hexuronic acid | 0.698525027 | 1-methyladenosine | 0.61359427 | alanine 3TMS | 0.646292433 | fucose | 0.710300549 |
| 5-aminovaleric acid lactame | 0.680677031 | erythronic acid lactone.1 | 0.612523591 | urea | 0.621746615 | idonic acid NIST | 0.700454192 |
| 1-methyladenosine | 0.677069814 | 5'-deoxy-5'-methylthioadenosine | 0.600487238 | caffeic acid | 0.620094109 | tartaric acid | 0.693689438 |
| hippuric acid 1TMS | 0.666125362 | (s)-(+)-mandelic acid | 0.597015125 | 1-methyladenosine | 0.607033689 | phosphoric acid | 0.692537675 |
| galactonic acid | 0.661598856 | gluconic acid lactone | 0.589269972 | galactinol major 2 | 0.605469045 | N-acetyl-D-mannosamine major | 0.691519483 |
| galactinol major 2 | 0.659814747 | threonic acid 2 | 0.586520832 | mannitol mix spec with histidine | 0.59628192 | 6-deoxyglucitol NIST | 0.684293998 |
| 5-hydroxy-3-indoleacetic acid | 0.630788269 | 2-hydroxyglutaric acid | 0.58204975 | isothreonic acid | 0.588491772 | cystine minor | 0.683360683 |
| ascorbic acid | 0.630499386 | 6-deoxyglucitol NIST | 0.558087742 | arabinose | 0.585835945 | conduritol betat epoxide minor | 0.657510038 |
| 5'-deoxy-5'-methylthioadenosine | 0.628841868 | cystine minor | 0.557391723 | pyrophosphate | 0.585129159 | 2-hydroxyglutaric acid | 0.656383369 |
| 3-hydroxybutanoic acid mix spec | 0.623950776 | glycerol-3-galactoside | 0.554649747 | erythronic acid lactone.1 | 0.57252609 | levoglucosan | 0.612095842 |
| urocanic acid | 0.617753342 | isothreonic acid | 0.552189767 | arachidic acid | 0.563622538 | octadecanol | 0.610193192 |
| glycerol-3-galactoside | 0.615446755 | galactonic acid | 0.545001537 | 2-deoxyerythritol NIST | 0.542120434 | indole-3-acetate | 0.604132818 |
| 3-hydroxy-3-indoleacetic acid | 0.611417657 | quinic acid | 0.523703127 | 6-deoxyglucitol NIST | 0.527239112 | adipic acid | 0.598572529 |
| cystine minor | 0.607102873 | octadecanol | 0.510378777 | mannose | 0.497186911 | glycerol-3-galactoside | 0.596042757 |
| 6-deoxyglucitol NIST | 0.588221404 | 3-hydroxy-3-indoleacetic acid | 0.508787298 | conduritol betat epoxide minor | 0.489243351 | cellobiotol | 0.583228117 |
| lactic acid | 0.585154999 | 2-hydroxyhippuric acid | 0.501251897 | mevalonic acid NIST | 0.47290143 | dehydroascorbic acid | 0.567065583 |
| citramalic acid | 0.580297296 | 5-hydroxy-3-indoleacetic acid | 0.498202757 | galactonic acid | 0.463216296 | galactonic acid | 0.5560033 |
| adipic acid | 0.562008101 | conduritol betat epoxide minor | 0.487960961 | fucose | 0.458849926 | 3-hydroxybutanoic acid mix spec | 0.520630238 |
| 2-hydroxyhippuric acid | 0.56103032 | salicylic acid | 0.452119391 | homovanillic and 4-hydroxymandelic acid - mixed spectrum | 0.444616258 | salicylic acid | 0.494748617 |
| salicylic acid | 0.556593395 | 2-deoxyerythritol | 0.42099689 | N-acetyl-D-mannosamine 3 | 0.400291529 | mannitol mix spec with histidine | 0.454271804 |
| quinic acid | 0.454998062 | urea | 0.420838691 | urocanic acid | 0.374934709 | uric acid (mix spec with myo-inositol) | 0.366300728 |
| Death | VIP | Overt HE | VIP | Transplant | VIP | Hospitalizations | VIP |
| 3-aminoisobutyric acid | 1.643070115 | xylitol | 1.872164501 | phosphoric acid.1 | 1.725698799 | glycolic acid | 1.827212618 |
| 2,3-dihydroxybutanoic acid NIST | 1.614889928 | 5-methoxytryptamine | 1.867140102 | 2-hydroxyvaleric acid | 1.701237906 | 3-hydroxypropionic acid | 1.7560861 |
| 2-hydroxy-2-methylbutanoic acid | 1.610992622 | citric acid | 1.805605851 | 2-deoxytetronic acid NIST | 1.692077564 | 2-hydroxyvaleric acid | 1.584754621 |
| 3-ureidopropionate | 1.605150109 | creatinine | 1.741549209 | butane-2,3-diol (NIST) | 1.650237473 | butane-2,3-diol (NIST) | 1.573148827 |
| creatinine | 1.596144716 | 2-hydroxy-2-methylbutanoic acid | 1.738767214 | serine minor | 1.630710133 | creatinine | 1.55118776 |
| 2-hydroxyvaleric acid | 1.54878599 | 2-hydroxyvaleric acid | 1.52753461 | leucine | 1.625056096 | 2,3-dihydroxybutanoic acid NIST | 1.545142682 |
| 5-methoxytryptamine | 1.528630586 | glutamine | 1.505993268 | adipic acid | 1.624222805 | fructose 2 | 1.542568903 |
| uracil | 1.482652188 | glycolic acid | 1.45153304 | creatinine | 1.61593923 | glycine | 1.530902016 |
| glycine TMS1x | 1.478569024 | dodecane | 1.414469964 | oxalic acid | 1.560206429 | glycine TMS1x | 1.491975324 |
| proline | 1.451424687 | azelaic acid | 1.414292736 | 2-hydroxy-2-methylbutanoic acid | 1.498640399 | azelaic acid | 1.411987043 |
| glycolic acid | 1.443983922 | histidine | 1.403092474 | glycine TMS1x | 1.46031286 | fructose 1 | 1.409805348 |
| xylitol | 1.399886017 | threitol 2 | 1.40223294 | indole-3-lactate | 1.460261311 | 2-hydroxy-2-methylbutanoic acid | 1.397753125 |
| 3-hydroxypropionic acid | 1.397233101 | UDP-glucuronic acid | 1.398986905 | 3-hydroxybutanoic acid mix spec | 1.443439445 | xylulose NIST | 1.391645123 |
| butane-2,3-diol (NIST) | 1.391963441 | N-methylalanine | 1.386943204 | isorhamnose | 1.420245818 | benzoic acid mix spec | 1.38144168 |
| pelargonic acid | 1.370941662 | glycine TMS1x | 1.382226302 | benzoic acid mix spec | 1.408113448 | cyclohexylamine NIST | 1.342356615 |
| benzoic acid mix spec | 1.352670887 | 2,3-dihydroxybutanoic acid NIST | 1.380952853 | 3-ureidopropionate | 1.407285453 | threitol 2 | 1.32700058 |
| 2-deoxyerythritol NIST | 1.342240486 | butane-2,3-diol (NIST) | 1.367090966 | galacturonic acid | 1.386165728 | dodecane | 1.304573934 |
| citric acid | 1.340997281 | shikimic acid | 1.349692331 | glycolic acid | 1.381902268 | uracil | 1.285124236 |
| shikimic acid | 1.332763509 | inulotriose 1 | 1.34653941 | dodecane | 1.315481375 | stearic acid | 1.281632065 |
| fructose 1 | 1.322880587 | benzoic acid mix spec | 1.343034592 | proline | 1.309401844 | histidine | 1.257383399 |
| 5-hydroxymethyl-2-furoic acid NIST | 1.318307827 | oleic acid | 1.329231889 | palmitic acid | 1.293881775 | glutamic acid | 1.244119679 |
| glycine | 1.316429198 | pelargonic acid | 1.324870728 | cyclohexylamine NIST | 1.291276952 | alanine | 1.21756936 |
| threitol 2 | 1.300298179 | 5-hydroxymethyl-2-furoic acid NIST | 1.322340278 | 2-hydroxyhippuric acid | 1.282603474 | lysine | 1.215900452 |
| inulotriose 1 | 1.298822839 | methionine | 1.313365419 | threitol 2 | 1.258926856 | butyrolactam NIST | 1.204686675 |
| 3-hydroxypyridine | 1.294602045 | 3-hydroxypropionic acid | 1.294859591 | valine | 1.254448658 | xylitol | 1.195380495 |
| ethanolamine | 1.289697637 | glycine | 1.291035868 | cholesterol | 1.250806762 | 1-methylinosine NIST | 1.19303807 |
| succinic acid | 1.275114141 | inositol allo- | 1.287595078 | fructose 1 | 1.242569425 | oleic acid | 1.192078904 |
| histidine | 1.268622049 | glutamic acid | 1.280902653 | methionine | 1.227120347 | phosphoric acid.1 | 1.191737034 |
| N-methylalanine | 1.253537739 | levoglucosan | 1.273998362 | N-methylalanine | 1.216667203 | inositol allo- | 1.178333157 |
| glucose 2 | 1.247157093 | proline | 1.251723264 | glycerol-3-galactoside | 1.213303976 | cysteine | 1.177505388 |
| serine 4TMS | 1.240844133 | palatinitol | 1.251498758 | citric acid | 1.190171662 | cholesterol | 1.175242222 |
| lyxose minor | 1.239851832 | serine | 1.243573886 | ethanolamine | 1.189983298 | glyceric acid | 1.149741049 |
| glutamic acid | 1.218545894 | glycerol | 1.236278381 | adenosine | 1.188483621 | aspartic acid | 1.145356442 |
| xylulose NIST | 1.218174205 | 2-deoxyerythritol NIST | 1.225663238 | 5-methoxytryptamine | 1.17668657 | leucine | 1.139100024 |
| dodecane | 1.217034051 | phosphoric acid.1 | 1.215215378 | xylulose NIST | 1.175654095 | valine | 1.135457006 |
| oxalic acid | 1.198505734 | succinic acid | 1.20070614 | 3-aminoisobutyric acid | 1.174255063 | methionine | 1.132005562 |
| glyceric acid | 1.195775329 | beta-alanine | 1.191819991 | isoleucine | 1.165970631 | palmitic acid | 1.129830499 |
| 2-deoxytetronic acid NIST | 1.194197927 | 3-aminoisobutyric acid | 1.187503694 | hypoxanthine mix spec with ornithine | 1.14888831 | 5-aminovaleric acid lactame | 1.128551607 |
| valine | 1.19287686 | cellobiotol | 1.181113194 | 2-deoxyerythritol | 1.137947782 | proline | 1.126178749 |
| palatinitol | 1.190519098 | arabinose | 1.180362761 | dehydroascorbic acid | 1.134110153 | indole-3-lactate | 1.125345781 |
| xanthine | 1.177359116 | lysine | 1.174012938 | glycerol | 1.121219354 | N-methylalanine | 1.124667884 |
| isoleucine | 1.17163608 | valine | 1.157215153 | 3-hydroxypropionic acid | 1.118028651 | N-acetyl-D-hexosamine | 1.122320511 |
| serine | 1.167340997 | caffeic acid | 1.151698057 | glycine | 1.109505358 | ethanolamine | 1.118669529 |
| levoglucosan | 1.156764777 | tryptophan | 1.150911709 | sucrose | 1.108546208 | beta-alanine | 1.103084497 |
| methionine | 1.149596902 | 4-hydroxyhippuric acid NIST | 1.146091316 | thymine | 1.099282672 | 3-hydroxypyridine | 1.095181936 |
| hypoxanthine mix spec with ornithine | 1.146693162 | ethanolamine | 1.134730567 | xanthine | 1.09099352 | erythritol | 1.093149036 |
| dehydroascorbic acid | 1.138156634 | leucine | 1.131020228 | UDP-glucuronic acid | 1.077512296 | 4-hydroxyhippuric acid NIST | 1.083035801 |
| tryptophan | 1.131796162 | xylulose NIST | 1.122493664 | glutamic acid | 1.074221804 | galacturonic acid | 1.071247664 |
| ribitol | 1.120727753 | indole-3-acetate | 1.115189561 | beta-alanine | 1.071864298 | isoleucine | 1.064136563 |
| tyrosine mz147 missing | 1.119557874 | stearic acid | 1.109352419 | quinic acid | 1.066691033 | 3-aminoisobutyric acid | 1.063105297 |
| furoylglycine NIST | 1.103361958 | erythritol | 1.101666057 | glutamine | 1.059350163 | mannose | 1.055396249 |
| erythritol | 1.097933954 | galacturonic acid | 1.087889908 | uracil | 1.057247609 | glucose 1 | 1.055018038 |
| propane-1,3-diol NIST | 1.084653582 | xanthine | 1.074537146 | furoylglycine NIST | 1.051523403 | pelargonic acid | 1.054984446 |
| oleic acid | 1.079930508 | 2-deoxytetronic acid NIST | 1.071836993 | fructose 2 | 1.050483308 | quinic acid | 1.054195605 |
| valine TMS1x | 1.077036683 | 5-aminovaleric acid lactame | 1.06381631 | 5'-deoxy-5'-methylthioadenosine | 1.042779822 | serine | 1.050204121 |
| oxoproline | 1.07307549 | mevalonic acid NIST | 1.059789151 | inulotriose 1 | 1.042727173 | lyxose minor | 1.041804011 |
| erythronic acid lactone.1 | 1.072961761 | propane-1,3-diol NIST | 1.058793102 | oxoproline | 1.039820082 | oxoproline | 1.033504757 |
| N-acetylaspartic acid 1 | 1.072494667 | lyxose minor | 1.05168362 | phosphoric acid | 1.034044087 | hypoxanthine mix spec with ornithine | 1.03316724 |
| leucine | 1.071119863 | adenosine | 1.042422404 | oleic acid | 1.02936118 | 5-hydroxymethyl-2-furoic acid NIST | 1.030615966 |
| inositol allo- | 1.061155542 | phosphoric acid | 1.041934467 | (s)-(+)-mandelic acid | 1.026369482 | arabinose | 1.019547957 |
| tartaric acid | 1.060937638 | fucose 1 + rhamnose 2 | 1.026283046 | valine TMS1x | 1.023552269 | capric acid | 1.015395375 |
| mannose | 1.04118438 | thymine | 1.017548858 | xylitol | 1.011762437 | 3-hydroxy-3-methylglutaric acid | 1.015290115 |
| cystine | 1.034497751 | cystine | 1.017393042 | 2,3-dihydroxybutanoic acid NIST | 1.00715157 | gluconic acid lactone | 1.009114431 |
| azelaic acid | 1.029719696 | butyrolactam NIST | 1.017163334 | stearic acid | 1.006388146 | glycerol | 1.006711163 |
| butyrolactam NIST | 1.023495116 | cysteine | 1.015323676 | erythritol | 1.004318202 | 3-ureidopropionate | 0.993330189 |
| cyclohexylamine NIST | 1.020852673 | uracil | 1.011488157 | serine | 1.003938426 | hippuric acid 1TMS | 0.9900487 |
| thymine | 1.018812005 | gluconic acid | 1.008860495 | threonine | 0.997408731 | cystine | 0.986583539 |
| 1-methylinosine NIST | 1.013596073 | hypoxanthine mix spec with ornithine | 1.003419199 | arabitol | 0.989521262 | glycocyamine major | 0.98522364 |
| palmitic acid | 0.997624499 | fructose 1 | 1.002451211 | lysine | 0.980866189 | phenylalanine | 0.982779061 |
| pyrogallol | 0.989960279 | arabitol | 0.997153262 | gluconic acid | 0.979923951 | 5-methoxytryptamine | 0.978297801 |
